# Hepatic steatosis and pyroptosis are induced by the hepatitis B virus X protein via B56α-METTL3 interaction-mediated m6A modification of the *NLRP3* mRNA

**DOI:** 10.1038/s41419-025-08019-8

**Published:** 2025-10-06

**Authors:** Ze-Bang Du, Tun Han, Yu-Xin Cai, Yu-Shi Shen, Jia-Shen Wu, Xiong Li, Hang-Tian Zhong, Bai-Heng Wu, Lei Zhang, Liang-Yu Wen, Xiao-Ming Luo, Zhong-Ning Lin, Yu-Chun Lin

**Affiliations:** 1 State Key Laboratory of Vaccines for Infectious Diseases, Xiang An Biomedicine Laboratory, Xiang’an Hospital of Xiamen University, National Innovation Platform for Industry-Education Integration in Vaccine Research, School of Public Health, Xiamen University, Xiamen, China; 2 Department of Preventive Medicine, School of Public Health, Chengdu Medical College, Chengdu, China

**Keywords:** Chronic inflammation, RNA modification

## Abstract

Metabolic dysfunction-associated steatohepatitis (MASH) is one of the fastest-growing chronic liver diseases and is characterized by excessive steatosis, inflammation, and progressive liver injury. The hepatitis B virus (HBV) X protein (HBx) is a major viral factor that contributes to the onset and progression of MASH. Emerging evidence highlights the role of epigenetic modifications, particularly N6-methyladenosine (m6A), as prevalent modifications of mRNAs that play crucial roles in MASH pathogenesis by regulating mRNA stability, translation, processing, and nuclear export. However, the epigenetic mechanisms by which m6A modification contributes to HBx-related MASH remain poorly defined. In this study, we observed that NOD-like receptor protein 3 (NLRP3)-dependent pyroptosis and intracellular lipid accumulation are markedly elevated in the livers of HBx-transgenic (HBx-Tg) mice in vivo and in HBx-expressing hepatocytes in vitro, exacerbating liver injury and driving MASH progression. Integrated metabolomic and transcriptomic analyses of HBx-Tg mice revealed distinct gene expression alterations, suggesting a key role for m6A modification in mediating hepatic inflammation and lipotoxicity. Mechanistically, we identified methyltransferase-like 3 (METTL3) as a critical positive regulator of this process. HBx upregulated METTL3 expression and the m6A level of *NLRP3* mRNA in HBx-expressing hepatocytes, whereas METTL3 knockdown or catalytic inactivation suppressed NLRP3-dependent pyroptosis. Further investigation revealed that METTL3 enhances *NLRP3* mRNA stability via m6A modification at A2748 site in the coding sequence. Moreover, the protein phosphatase 2A (PP2A) B56α subunit was found to interact with the METTL3 methyltransferase domain (MTD), facilitating its enzymatic activity and further increasing *NLRP3* m6A methylation, thereby promoting pyroptosis and lipid accumulation in HBx-expressing hepatocytes. Importantly, treatment with STM2457, a selective inhibitor targeting the METTL3 MTD, significantly attenuated hepatic inflammation, steatohepatitis, and lipotoxicity. Taken together, our findings advance the understanding of HBx-induced hepatic lipid accumulation, steatosis, inflammasome formation, and pyroptosis, and indicate that targeting METTL3 with STM2457 intervention is a promising approach for MASH treatment.

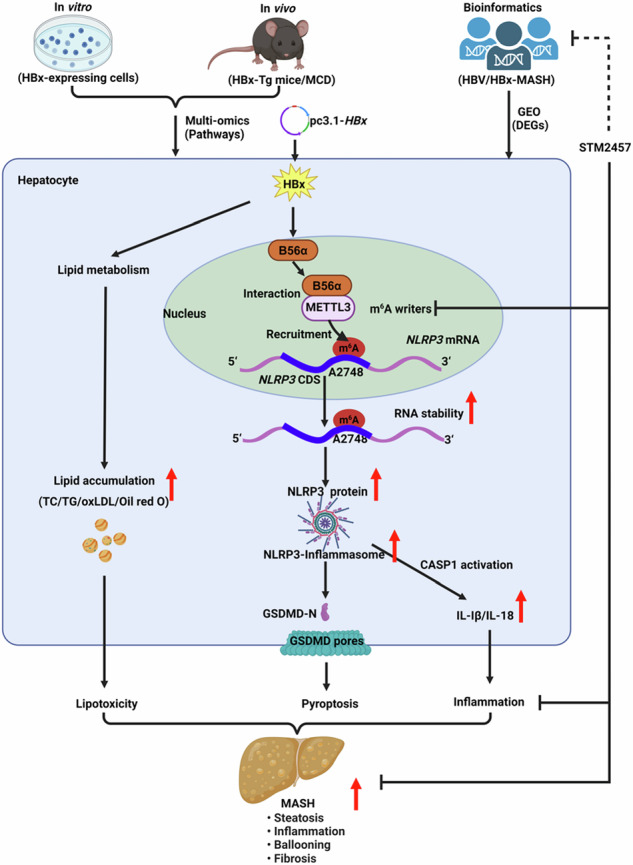

## Introduction

Metabolic dysfunction-associated steatotic liver disease (MASLD), formerly known as nonalcoholic fatty liver disease (NAFLD), is a prevalent chronic liver disorder characterized by lipid accumulation in hepatocytes. It affects up to 32% of the global population and has become a major public health issue [1, 2]. MASLD spans a spectrum from relatively benign metabolic dysfunction-associated fatty liver (MAFL) to more severe metabolic dysfunction-associated steatohepatitis (MASH), which can progress to cirrhosis and hepatocellular carcinoma [3, 4]. The etiology of MASH is complex and involves environmental exposure, genetic susceptibility, intestinal microbiota, and metabolic factors [5, 6]. Recent research has also highlighted the contribution of hepatitis B virus (HBV) infection to MASH pathogenesis [7], with HBV increasingly considered a metabolic virus. Chronic HBV infection may influence metabolic changes leading to MASLD development. HBV-induced inflammatory responses and molecular pathways may aggravate hepatic steatosis [8]. In particular, the HBV X protein (HBx) has been shown to upregulate lipid metabolism genes, stimulate hepatic lipid accumulation, and trigger inflammatory responses, thereby exacerbating hepatic steatosis [9–11]. These observational and experimental data support a role for HBV/HBx in enhancing hepatic inflammation and lipid accumulation, consequently contributing to liver injury and MASH. Similarly, our previous work revealed that HBx expression disrupts hepatic lipid homeostasis and intensifies liver injury in HBx-transgenic (HBx-Tg) mice [12]. However, the complete impact of liver inflammation in HBx-Tg mice and the molecular mechanisms underlying HBV-induced hepatic lipid accumulation remain insufficiently understood, necessitating further investigation into the pathogenesis of HBx-related MASH.

N6-methyladenosine (m6A) is a predominant and conserved post-transcriptional modification of eukaryotic mRNAs and is among the more than 100 distinct chemical modifications found in vivo [13]. The homeostasis of m6A modification is reversible and dynamically regulated by the balance among methyltransferases (writers), demethylases (erasers), and reading proteins (readers). The methyltransferase complex—comprising methyltransferase-like 3 (METTL3), METTL14, and Wilms tumor 1-associated protein (WTAP)—catalyzes the addition of m6A, while demethylases remove it, and reader proteins mediate downstream regulatory effects [14]. Increasing evidence has demonstrated that m6A is involved in a variety of human liver diseases. In particular, recent studies have shown that m6A significantly influences the onset and progression of MASH by regulating lipid metabolism [15, 16], hepatocyte inflammation [17], and insulin resistance, suggesting its potential as a therapeutic target. Therefore, investigating the mechanisms and aberrant m6A modifications in MASH is crucial for developing novel therapeutic targets and prognostic markers. The interaction of the HBx protein with METTL3/14 proteins stimulates their nuclear import and recruits them to transcription initiation sites to affect the internal m6A modification of *PTEN* mRNA during HBV infection [18, 19]. However, the precise mechanism by which METTL3-mediated m6A methylation contributes to HBx-related MASH remains unclear.

Inflammasomes are widely recognized as critical mediators of the pro-inflammatory response in MASH. Although multiple inflammasome types exist, most studies to date have focused on the NOD-like receptor protein 3 (NLRP3) inflammasome in MASH pathogenesis [20–22]. Activation of the NLRP3 inflammasome triggers conversion of pro-caspase-1 to cleaved caspase-1 and cleavage of the N- and C-termini of gasdermin D (GSDMD), thereby initiating the canonical pyroptosis pathway. Previous studies have demonstrated that HBx activates the NLRP3 inflammasome and promotes pyroptosis in hepatocytes [23]. Moreover, under oxidative stress, HBx has been reported to activate NLRP3 via mitochondrial reactive oxygen species (ROS), leading to IL-1β and IL-18 release from hepatocytes [24]. However, the epigenetic mechanisms by which HBx regulates the post-transcriptional modification of *NLRP3* mRNA in MASH remain unclear. We hypothesized that m6A modification of the *NLRP3* mRNA may mediate hepatocyte pyroptosis and contribute to MASH progression.

This study investigated the role of METTL3-mediated m6A modification of *NLRP3* mRNA in HBx-related MASH. Our findings revealed that upregulated METTL3 enhanced *NLRP3* mRNA stability via m6A modification at the A2748 site, leading to NLRP3-dependent pyroptosis and lipotoxicity in HBx-Tg mice and HBx-expressing hepatocytes. Furthermore, the interaction between the protein phosphatase 2A (PP2A) B56α subunit and METTL3 modulated m6A methyltransferase activity and global RNA m6A levels. Inhibition of METTL3 activity by STM2457 alleviated lipotoxicity and pyroptotic phenotypes in MASH. These results suggest a novel regulatory mechanism involving the B56α/METTL3-NLRP3 axis in m6A modification regulation and identify potential targets for intervention in HBx-related MASH.

## Materials and methods

Materials and methods are described in the Supplementary Files.

## Results

### m6A modification is associated with lipotoxicity and NLRP3-mediated inflammation in HBx-related MASH in transgenic mice

Chronic HBV infection has previously been implicated in lipotoxicity and MASH development, and HBV-induced inflammatory pathways potentially exacerbate hepatic steatosis [8]. We performed transcriptomic and untargeted metabolomic analyses of livers from HBx-Tg mice and WT mice (Fig. S1A). Transcriptome analysis identified 859 upregulated and 1066 downregulated differentially expressed genes (DEGs), including genes involved in lipid metabolism (e.g., *Fabp2*, *Apoa4*, *Fasn*) and inflammation (e.g., *Nlrp3*, *Pycard*), indicating disrupted lipid homeostasis and activation of NLRP3-related pathways (Fig. S1B, C). GO enrichment analysis confirmed that the DEGs were mainly related to fatty acid metabolic process, triglyceride metabolic process, lipid storage, inflammatory response, and lipid droplet pathways (Fig. S1D). Metabolomic analysis revealed 104 upregulated and 35 downregulated differentially expressed metabolites (DEMs) in HBx-Tg mice. A heatmap revealed that HBx increased DEMs involved in bile acid metabolism (e.g., chenodeoxycholic acid, deoxycholic acid), lipid metabolism (e.g., linoleic acid, stearic acid), and arachidonic acid metabolism (e.g., prostaglandin H2, prostaglandin G2) (Fig. S1E). KEGG enrichment analysis confirmed that the DEMs were mainly enriched in fatty acid biosynthesis, arachidonic acid metabolism, and bile secretion pathways (Fig. S1F). Integrative analysis revealed a strong correlation between lipid- and inflammation-related DEGs and DEMs, linking HBx expression to MASH-related metabolic disturbances (Fig. S1G).

**Fig. 1:**
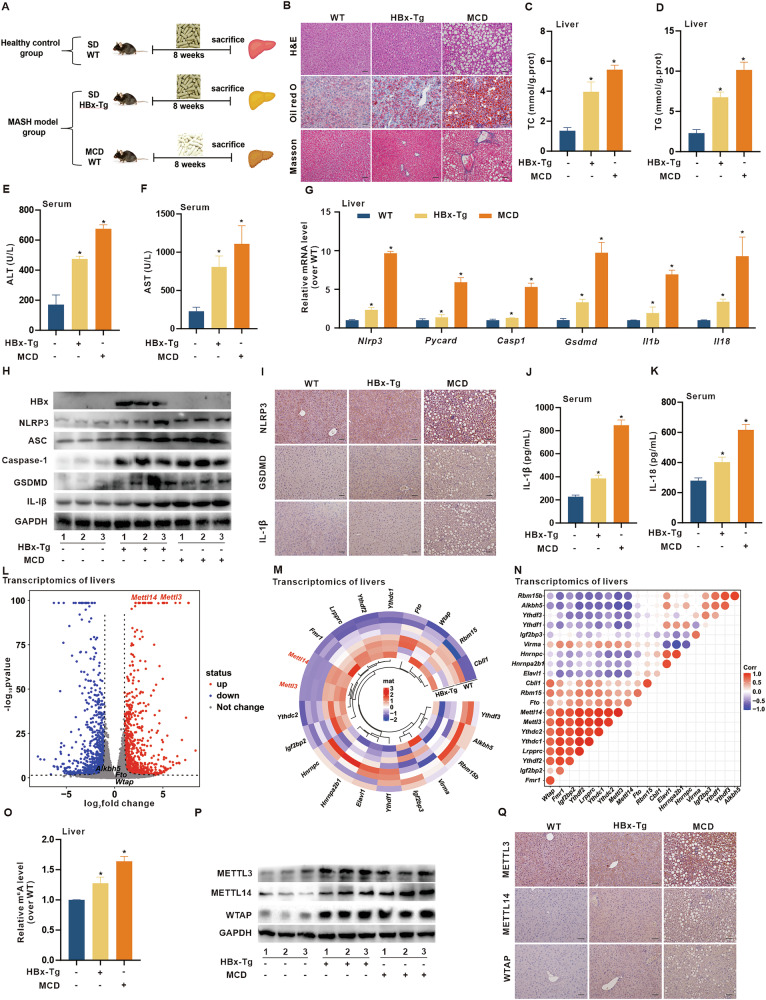
m6A modification is associated with lipotoxicity and NLRP3-mediated inflammation in HBx-related MASH in transgenic mice. HBx-Tg mice were used to construct the MASH model, whereas C57BL/6 WT mice served as the negative control and MCD-fed mice as the positive model. *n* = 5 per group. **A** Schematic of the experimental MASH model in mice. **B** Representative images showing H&E, Oil red O, and Masson staining of liver tissues illustrating the histopathological phenotypes of MASH. Scale bar, 100 μm. **C**, **D** Levels of total cholesterol (TC) (**C**) and triglyceride (TG) (**D**) in liver tissues were detected using colorimetric assays. **E**, **F** Levels of ALT (**E**) and AST (**F**) in serum. **G** Relative mRNA levels of *Nlrp3* and pyroptosis-related genes were measured by qRT-PCR. **H** Levels of HBx expression and NLRP3, ASC, Caspase-1, GSDMD, and IL-1β proteins were measured by WB. *n* = 3. **I** Representative images showing the IHC staining of NLRP3, GSDMD, and IL-1β in liver tissues. Scale bar, 100 μm. **J**, **K** Levels of IL-1β (**J**) and IL-18 (**K**) in serum. **L**, **M** Transcriptomic profiling was performed on liver tissues from WT mice (*n* = 4) and HBx-Tg mice (*n* = 4). **L** Volcano plot of DEGs. **M** Heatmap of m6A-related genes. **N** Pearson correlation analysis showing the relationship between mRNA levels of m6A-related genes. **O** Global RNA m6A levels in liver tissues were detected using colorimetric assays. **P** Protein levels of METTL3, METTL14, and WTAP were measured by WB. **Q** Representative images showing the IHC staining of METTL3, METTL14, and WTAP in liver tissues. Scale bar, 100 μm. Data are presented as mean ± SD. *, *P* < 0.05, compared to the WT group.

To validate these changes in vivo, we established a MASH model in HBx-Tg mice and compared it with an MCD-fed group serving as a positive control (Fig. 1A). Although body weight remained unchanged (Fig. S1H), the liver/body weight ratio significantly increased in both the HBx-Tg and MCD groups compared with the WT group (Fig. S1I). Histological examination of the HBx-Tg livers, as well as the MCD group, revealed typical MASH features—hepatocyte ballooning, vacuolization, inflammatory infiltration, lipid droplet (LD) accumulation, and fibrosis (Fig. 1B). Similarly, the levels of MASH indices, including the lipid indicators TC, TG, and ox-LDL (Fig. 1C, D and S1J), the oxidative stress marker GPx (Fig. S1K), and the liver function and toxicity indicators ALT, AST, and ALP (Fig. 1E, F and S1L), were considerably higher in the HBx-Tg and MCD groups than in the WT group, confirming MASH phenotypes in HBx-Tg mice. Given the known role of the NLRP3 inflammasome in MASH progression [25], our transcriptomic analysis also revealed significant activation of the NLRP3-related inflammatory pathways in the livers of HBx-Tg mice. We next assessed NLRP3-related inflammation in HBx-Tg mice. The mRNA and protein levels of NLRP3, ASC, Caspase-1, GSDMD, IL-1β, and IL-18 were significantly increased in the HBx-Tg livers compared with the WT group (Fig. 1G, H). IHC staining revealed that the levels of NLRP3, GSDMD, and IL-1β (Fig. 1I), as well as the levels of serum IL-1β, IL-18, and IL-6 (Fig. 1J, K and S1M), were increased in the HBx-Tg group compared with the WT group. These results indicate that HBx expression significantly promotes NLRP3-mediated inflammation in HBx-related MASH in vivo.

Recent studies have implicated m6A RNA methylation in hepatic lipid and inflammatory disorders [26]. Transcriptomic analysis revealed elevated expression of m6A writers (*Mettl3*, *Mettl14*, and *Wtap)* in the livers of HBx-Tg mice (Fig. 1L, M), with strong inter-gene correlation (Fig. 1N) and increased global RNA m6A methylation levels (Fig. 1O). These findings were validated by RT‒qPCR and western blotting, confirming upregulation of METTL3, METTL14, and WTAP (Fig. 1P, Q and S1N). These results suggest that the increase in m6A RNA methylation is involved in HBx-related MASH pathogenesis in vivo.

### HBx upregulates NLRP3-mediated pyroptosis and MASH-associated METTL3 in vitro

**Fig. 2:**
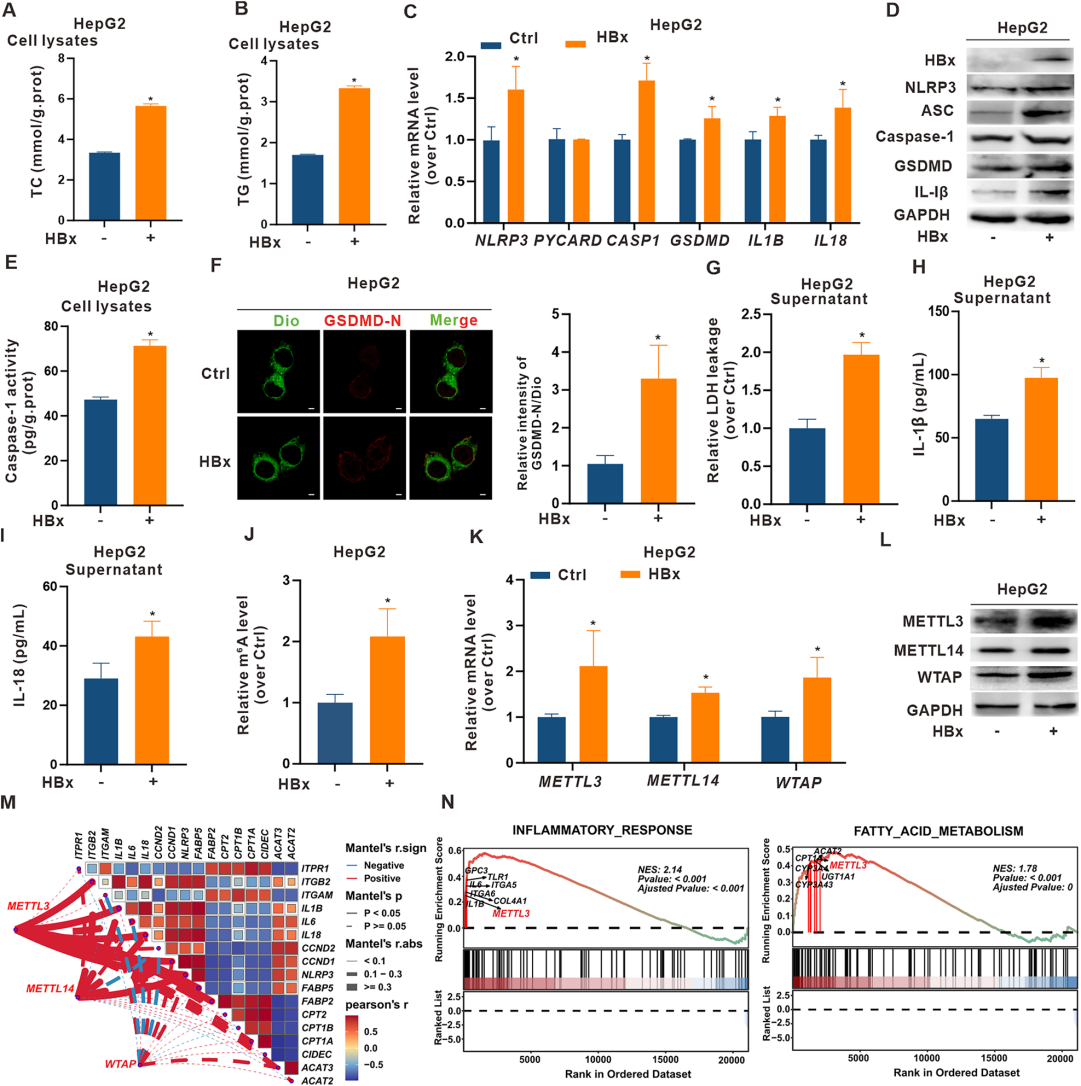
HBx upregulates NLRP3-mediated pyroptosis and MASH-associated METTL3 in HBx-expressing hepatocytes in vitro. **A**–**L** HepG2 cells were transfected with pcDNA3.1-HBx (1 μg/ml, 24 h) to construct HBx-expressing hepatocytes, while the negative control (NC) cells transfected with pcDNA3.1 vector served as controls. **A**, **B** Levels of TC (**A**) and TG (**B**) in cells. **C** Relative mRNA levels of *NLRP3* and pyroptosis-related genes were measured by qRT-PCR. **D** Levels of HBx expression and NLRP3, ASC, Caspase-1, GSDMD, and IL-1β proteins were detected by WB. **E** The activity of caspase-1 related to pyroptosis was quantified by ELISA. **F** Representative images showing co-localization of GSDMD-N (red) and Dio (green) were captured by confocal microscopy (Left). Scale bar, 10 µm. Quantification of the relative intensity of GSDMD-N/Dio is shown in a bar graph (Right). **G**–**I** Levels of LDH (**G**), IL-1β (**H**), and IL-18 (**I**) in supernatants. **J** Global RNA m6A levels in cells were measured by colorimetric assays. **K** Relative mRNA levels of *METTL3*, *METTL14*, and *WTAP* were detected by qRT-PCR. **L** Expression levels of METTL3, METTL14, and WTAP proteins were detected by WB. (M-N) A liver transcriptome dataset (GSE89632) was derived from NASH patients (*n* = 19) and healthy controls (HC, *n* = 24). **M** Correlation analysis between methyltransferase-related genes and inflammation- or lipid metabolism-related genes. **N** GSEA was performed. Data are presented as mean ± SD. *, *P* < 0.05, compared to the control group.

To further explore the mechanism underlying HBx-related MASH, we used HBx-expressing HepG2 and differentiated HepaRG cells as models based on our previous studies [27]. The cell models showed dramatically increased TC and TG levels in HBx-expressing hepatocytes (Fig. 2A-B and S2A-B), indicating induced lipotoxicity. Our previous study revealed that HBx-Tg mice exhibited NLRP3 inflammasome activation, inflammatory infiltration, and increased hepatic steatosis [12]. Given the critical role of the NLRP3 inflammasome in hepatocyte pyroptosis, we hypothesized that the activated NLRP3 inflammasome may regulate pyroptosis-associated MASH in HBx-expressing hepatocytes. The mRNA levels of pyroptosis-related genes, including *NLRP3*, *PYCARD*, *CASP1*, *GSDMD*, *IL1B*, and *IL18*, were increased in HBx-expressing hepatocytes (Fig. 2C and S2C), accompanied by elevated protein levels of NLRP3 and inflammasome components (Fig. 2D and S2D). Subsequently, caspase-1 activity (Fig. 2E and S2E), membrane-localized GSDMD-N (Fig. 2F and S2F), and LDH, IL-1β, and IL-18 release (Fig. 2G–I and S2G–I) were also significantly increased. These results suggest that HBx induces hepatocyte pyroptosis by regulating NLRP3-dependent inflammasome activation.

Given the emerging role of m6A modification in disease regulation [28], we next assessed its involvement in HBx-expressing hepatocytes. In line with the findings of the MASH mouse model (Fig. 1L–Q and S1N), global RNA m6A levels were increased (Fig. 2J and S2J), as were the mRNA and protein levels of METTL3, METTL14, and WTAP (Fig. 2K, L and S2K, L) in HBx-expressing hepatocytes. Similarly, we analyzed liver transcriptome data (GSE89632) from both NASH patients (*n* = 19) and healthy controls (HCs) (*n* = 24), and detected significant differences in m6A-related genes, particularly in *METTL3*, *METTL14*, and *WTAP* (Fig. S2M). Correlation analysis revealed a significant association between these methyltransferase-related genes and inflammation- or lipid metabolism-related genes (Fig. 2M).

Although METTL14 and WTAP were also upregulated in the above study, METTL3 was prioritized for downstream investigation because of its unique catalytic function and predominant upregulation. Specifically, METTL3 serves as the enzymatic core of the m6A methyltransferase complex, harboring the methyltransferase domain (MTD) directly responsible for m6A deposition. In contrast, METTL14 primarily acts as an RNA-binding cofactor, while WTAP serves as a structural scaffold, neither of which possesses catalytic activity. Moreover, GSEA enrichment analyses further highlighted the involvement of METTL3 in inflammatory and fatty acid metabolism pathways (Fig. 2N), and correlation analysis revealed stronger associations between *METTL3* expression and key pyroptosis or inflammation markers (e.g., *IL1B*, *IL6*, *IL18*, and *NLRP3*) (Fig. S2N). Together, these results suggest that MASH-associated functional regulation of METTL3-mediated m6A modification may be involved in promoting NLRP3 inflammasome-dependent pyroptosis in HBx-expressing hepatocytes.

### METTL3 targets *NLRP3* mRNA to mediate pyroptosis in HBx-expressing hepatocytes

**Fig. 3:**
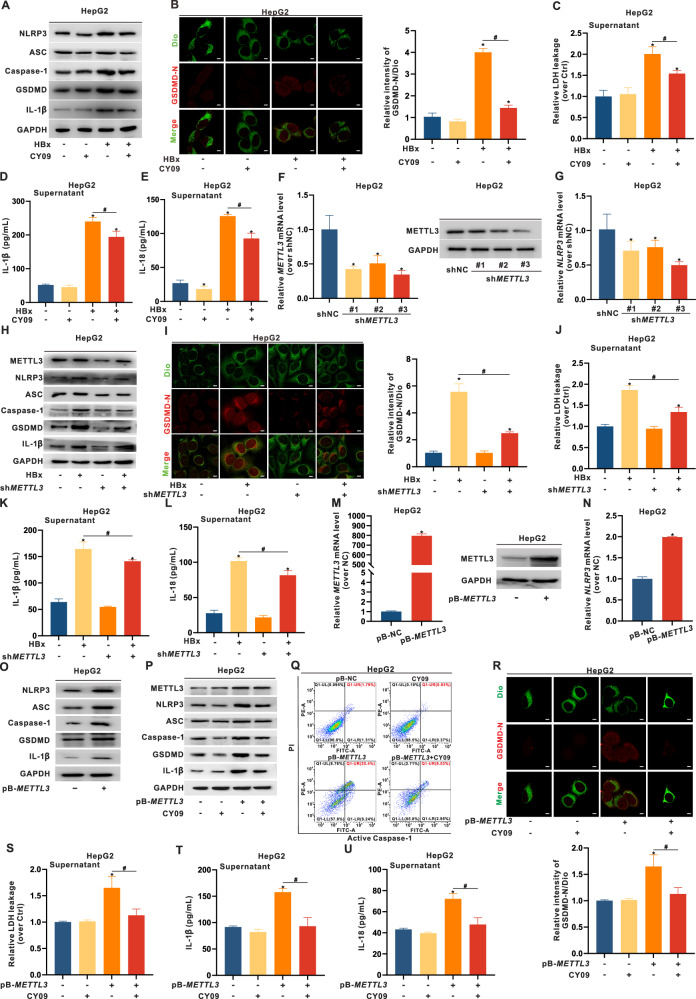
METTL3 targets *NLRP3* mRNA to mediate pyroptosis in HBx-expressing HepG2 cells. **A**–**E** HepG2 cells were transfected with pcDNA3.1-HBx (1 μg/mL, 24 h) to construct the HBx-expressing hepatocytes, followed by treatment with or without CY-09 (10 μM, 24 h), while CY-09 was used as a specific inhibitor of NLRP3 activity. **A** Expression levels of NLRP3, ASC, Caspase-1, GSDMD, and IL-1β proteins were detected by WB. **B** Representative IF images showing co-localization of GSDMD-N (red) and Dio (green) were captured by confocal microscopy (Left). Scale bar, 10 µm. Quantification of the relative intensity of GSDMD-N/Dio is shown in a bar graph (Right). **C**–**E** Levels of LDH (**C**), IL-1β (**D**), and IL-18 (**E**) in supernatants. **F**–**L** HBx-expressing HepG2 cells were transfected with sh*METTL3* (1 μg/mL, 24 h) to knock down METTL3, while shNC was transfected as a negative control. **F** Levels of *METTL3* mRNA and METTL3 protein in cells knocked down by serial sh*METTL3* (#1-#3). **G** The level of *NLRP3* mRNA was detected. **H** Expression levels of METTL3, NLRP3, and pyroptosis-related proteins. **I** Representative IF images showing co-localization of GSDMD-N (red) and Dio (green) in cells (Left), while the quantification is shown in a bar graph (Right). Scale bar, 10 µm. **J**–**L** Levels of LDH (**J**), IL-1β (**K**), and IL-18 (**L**) in supernatants. **M**–**O** A pB-*METTL3* recombinant plasmid was constructed and transfected to construct the stable METTL3 overexpression (OE) in HepG2 cells, while pB-NC was used as a control. **M** Levels of *METTL3* mRNA and METTL3 protein in cells. **N** The level of *NLRP3* mRNA was detected. **O** Expression levels of NLRP3 and pyroptosis-related proteins in cells. **P**–**U** METTL3-overexpressing HepG2 cells were pre-treated with or without CY-09 (10 μM, 24 h) to inhibit NLRP3 activity. **P** Expression levels of METTL3, NLRP3, and pyroptosis-related proteins. **Q** Representative images showing the flow cytometry quantification of caspase-1 activity related to pyroptosis. **R** Representative IF images showing co-localization of GSDMD-N (red) and Dio (green) (Upper), while the quantification is shown in a bar graph (Lower). Scale bar, 10 µm. **S**–**U** Levels of LDH (**S**), IL-1β (**T**), and IL-18 (**U**) in supernatants. Data are presented as mean ± SD. *, *P* < 0.05, compared to the control group. #, *P* < 0.05, compared to the corresponding group.

The above experiments demonstrated that HBx significantly induces NLRP3-related inflammation and lipotoxicity in mouse livers, along with NLRP3-dependent pyroptosis and lipid accumulation in hepatocytes. Previous studies have shown that CY-09, a direct and specific inhibitor of the NLRP3 inflammasome, significantly inhibits the progression of MASH [29–32]. As shown in Fig. 3A and S3A, the increased protein expression of NLRP3 and its downstream pyroptosis-related proteins in HBx-expressing hepatocytes was significantly inhibited by CY-09 treatment. Moreover, GSDMD-N membrane co-localization was suppressed (Fig. 3B and S3B), accompanied by decreased LDH leakage and reduced release of IL-1β and IL-18 (Fig. 3C-E and S3C–E).

METTL3 has been shown to play a crucial role in the progression of MASH via targeted m6A modification of specific mRNAs [13, 33]. However, whether METTL3 regulates NLRP3-dependent pyroptosis in HBx-expressing hepatocytes through mRNA-level modulation remains unclear. To further investigate this regulatory axis, we established stable METTL3-knockdown HepG2 and HepaRG cells (Fig. 3F and S3F). METTL3 knockdown resulted in a concurrent decrease in *NLRP3* mRNA expression (Fig. 3G and S3G). Notably, silencing METTL3 in HBx-expressing hepatocytes markedly suppressed HBx-induced NLRP3 inflammasome activation (Fig. 3H and S3H), reduced GSDMD-N membrane translocation (Fig. 3I and S3I), and significantly decreased LDH, IL-1β, and IL-18 release (Fig. 3J–L and S3J–L). These results demonstrate that METTL3 knockdown alleviates HBx-induced NLRP3 inflammasome-dependent pyroptosis.

To validate this regulatory relationship, we next generated METTL3-overexpressing HepG2 and HepaRG cells (Fig. 3M and S3M). Overexpression of METTL3 led to elevated NLRP3 mRNA and protein levels, accompanied by increased inflammasome activation (Fig. 3N, O and S3N, O). Furthermore, intervention with CY-09 effectively reversed METTL3-mediated inflammasome activation (Fig. 3P and S3P), suppressed caspase-1 activity (Fig. 3Q and S3Q), reduced GSDMD-N membrane co-localization (Fig. 3R and S3R), and inhibited LDH, IL-1β, and IL-18 release (Fig. 3S-U and S3S–U).

To determine whether the enzymatic activity of METTL3 is required for these effects, we introduced a catalytically inactive METTL3 mutant (METTL3-MUT) into HBx-expressing hepatocytes (Fig. S4A). Unlike wild-type METTL3 (METTL3-WT), the mutant failed to increase the level of *NLRP3* mRNA, did not activate the NLRP3 inflammasome, and did not induce GSDMD-N membrane co-localization or pyroptosis-related LDH, IL-1β, and IL-18 release (Fig. S4B–H). Collectively, these findings demonstrate that METTL3 promotes *NLRP3* mRNA stability and inflammasome activation in an m6A methyltransferase activity-dependent manner, thereby driving NLRP3-dependent pyroptosis in HBx-expressing hepatocytes.

### METTL3-targeting function regulates *NLRP3* mRNA stability in an A2748 site m6A-dependent manner

**Fig. 4:**
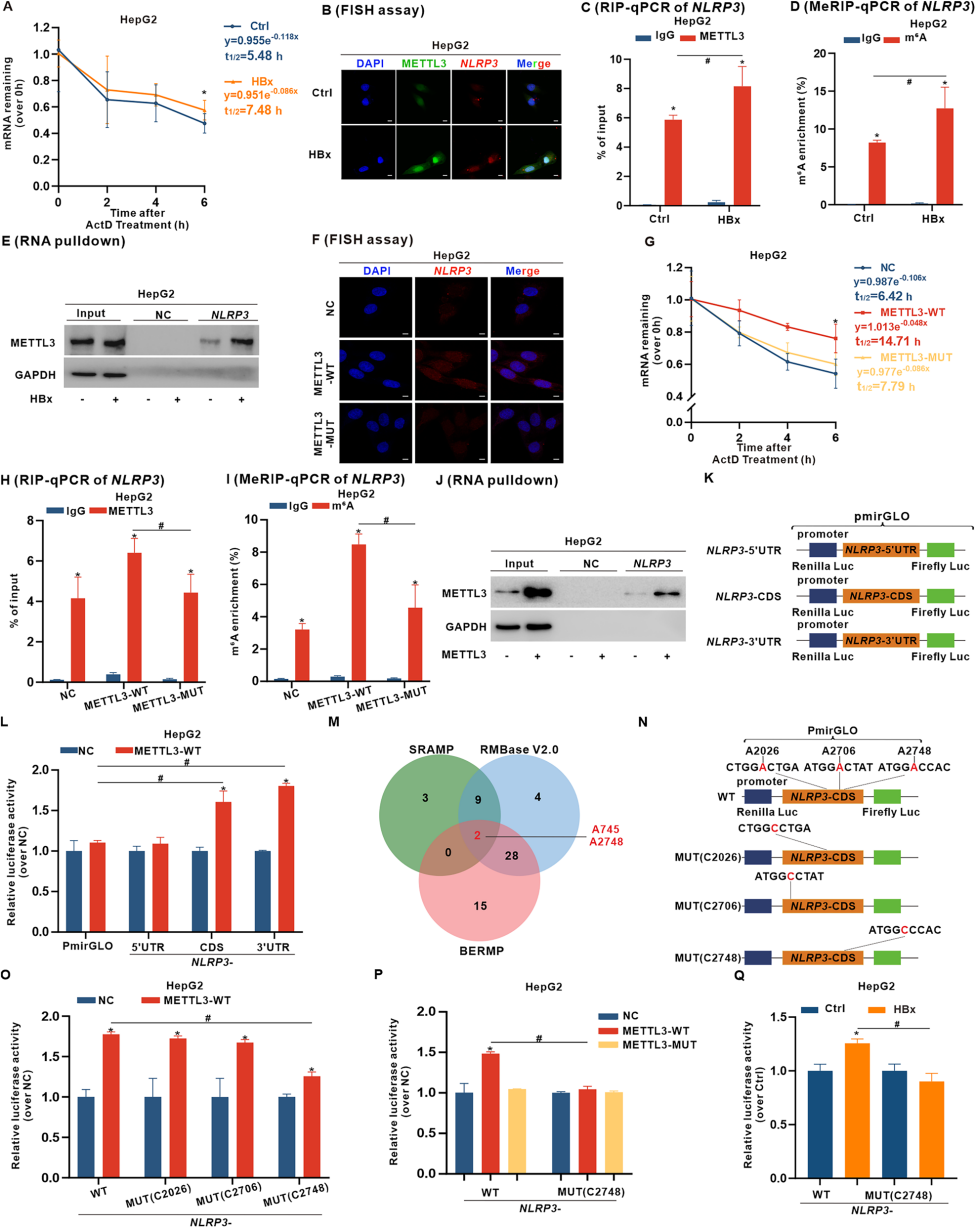
METTL3-targeting function regulates *NLRP3* mRNA stability in an A2748 site m6A-dependent manner in HBx-expressing HepG2 cells. **A**–**E** HepG2 cells were transfected with pcDNA3.1-HBx (1 μg/ml, 24 h) to construct HBx-expressing hepatocytes, while the negative control (NC) cells transfected with pcDNA3.1 vector served as controls. **A** The mRNA stability assay for half-life of *NLRP3* mRNA transcript was measured by qRT-PCR in cells treated with actinomycin D (ActD, 5 μg/ml) at the indicated time points. **B** Representative FISH images showing the staining of METTL3 (green) and *NLRP3* mRNA (red) in cells, while DAPI (blue) was used to counterstain the nuclei. Scale bar, 20 µm. **C** RIP-qPCR analysis of *NLRP3* mRNA illustrating the binding interaction between *NLRP3* mRNA and METTL3. **D** MeRIP-qPCR analysis of m6A levels on *NLRP3* mRNA. **E** RNA pulldown assay using an *NLRP3* mRNA probe followed by detection of METTL3 protein by WB. **F**–**I** Stable METTL3-overexpressing HepG2 cells were generated using recombinant plasmids encoding wild-type (METTL3-WT) or a catalytically inactive mutant (DWWP to AWWA) (METTL3-MUT). **F** Representative FISH images showing the staining of *NLRP3* mRNA (red) and DAPI (blue) in cells. Scale bar, 20 µm. **G** Half-life of *NLRP3* mRNA transcript was measured by the mRNA stability assay. **H** RIP-qPCR analysis of *NLRP3* mRNA illustrating the binding interaction between *NLRP3* mRNA and METTL3. **I** MeRIP-qPCR analysis of m6A levels on *NLRP3* mRNA. **J** RNA pulldown assay using an *NLRP3* mRNA probe followed by detection of METTL3 protein by WB. **K**, **L** pmirGLO-*NLRP3*-5′UTR, -CDS, and -3′UTR reporter gene recombinant plasmids were constructed by inserting the corresponding fragments of *NLRP3* mRNA (**K**), followed by transfection into METTL3-WT-overexpressing HepG2 cells (**L**). **L** Relative luciferase activities of the reporters were detected in cells. **M** Based on the online databases, including SRAMP, BERMP, and RMBase V2.0, a Venn plot shows the predicted m6A modification sites in *NLRP3* mRNA. **N**–**Q** pmirGLO-*NLRP3-*WT, -MUT(C2026), -MUT(C2706), and -MUT(C2748) luciferase reporter gene recombinant plasmids were constructed by inserting WT or site-directed mutant (MUT) *NLRP3* CDS fragments (**N**), followed by transfection into cells. **O** Relative luciferase activities of *NLRP3-*WT and -MUT luciferase reporters in NC or METTL3-WT-overexpressing HepG2 cells. **P** Relative luciferase activities of *NLRP3-*WT or -MUT(C2748) luciferase reporters in NC, METTL3-WT-, and METTL3-MUT-overexpressing HepG2 cells. **Q** Relative luciferase activities of *NLRP3-*WT or -MUT(C2748) luciferase reporters in HBx-expressing HepG2 cells. Data are presented as mean ± SD, *n* = 3. *, *P* < 0.05, compared to the control group. #, *P* < 0.05, compared to the corresponding group.

To explore the mechanism of METTL3-mediated upregulation of NLRP3 in HBx-expressing hepatocytes, we assessed *NLRP3* mRNA stability. HBx significantly prolonged the half-life of *NLRP3* mRNA in HBx-expressing hepatocytes (Fig. 4A and S5A), suggesting that enhanced mRNA stability contributes to the increased expression. Additionally, fluorescence in situ hybridization (FISH) assays further revealed enhanced co-localization of METTL3 and *NLRP3* mRNA in HBx-expressing cells (Fig. 4B and S5B), suggesting a potential interaction. RNA immunoprecipitation (RIP), methylated RNA immunoprecipitation (MeRIP), and RNA pulldown assays confirmed that HBx promoted METTL3–*NLRP3* mRNA binding and m6A enrichment (Fig. 4C–E and S5C–E). These findings suggest that HBx enhances METTL3-mediated m6A modification of *NLRP3* mRNA, leading to increased mRNA stability and upregulation of NLRP3 protein in HBx-expressing cells.

We further investigated the role of METTL3 functional m6A methyltransferase activity in regulating NLRP3 expression. Ectopically expressed METTL3-WT, but not the catalytically inactive METTL3-MUT, significantly upregulated *NLRP3* mRNA levels (Fig. 4F and S5F). Moreover, the results of the mRNA stability assay revealed that only METTL3-WT extended the half-life of *NLRP3* mRNA (Fig. 4G and S5G). Subsequently, RIP, MeRIP, and RNA pulldown assays revealed that METTL3-WT significantly promoted the binding interaction between METTL3 and *NLRP3* mRNA and increased the m6A modification level of *NLRP3* mRNA, whereas METTL3-MUT cells showed decreased binding and m6A levels (Fig. 4H–J and S5H–J). These results suggest that the m6A catalytic activity of METTL3 is essential for *NLRP3* mRNA stability and upregulation.

To identify specific m6A sites involved in METTL3-mediated regulation of *NLRP3* mRNA, we constructed pmirGLO-*NLRP3*-5′UTR, -CDS, and -3′UTR recombinant plasmids by inserting the corresponding fragments of *NLRP3* mRNA (Fig. 4K). In METTL3-WT cells, luciferase activities of *NLRP3*-CDS and *NLRP3*-3′UTR were increased (Fig. 4L and S5K), suggesting that m6A modification sites within *NLRP3*-CDS or the *NLRP3*-3′UTR are indispensable for *NLRP3* mRNA upregulation. Using online databases, including SRAMP, BERMP, and RMBase V2.0 [34, 35], we predicted two potential m6A modification sites (A745 and A2748) in the CDS region of *NLRP3* mRNA (Fig. 4M). Moreover, three high confidence m6A sites in *NLRP3*-CDS predicted by SRAMP were site-directed mutated from adenine (A) to cytosine (C), yielding mutants *NLRP3*-MUT(C2026), -MUT(C2706), and -MUT(C2748) (Fig. 4N). Luciferase activity of *NLRP3*-MUT(C2748) was clearly lower than that of *NLRP3*-WT (Fig. 4O and S5L), suggesting that A2748 site may be a critical m6A motif for the functional modulation of *NLRP3* mRNA. Furthermore, to confirm A2748 site-specific m6A modification of *NLRP3* mRNA, we assessed METTL3-dependent effects related to HBx expression. METTL3-WT, but not METTL3-MUT, substantially promoted luciferase activity of *NLRP3*-WT (with A2748), whereas *NLRP3*-MUT (with C2748) showed no change (Fig. 4P and S5M), demonstrating that METTL3 regulates the expression of *NLRP3* through an A2748 m6A-dependent mechanism. Additionally, luciferase activity of *NLRP3*-WT (with A2748) increased in HBx-expressing hepatocytes, while *NLRP3*-MUT (with C2748) remained unchanged across all groups of cells (Fig. 4Q and S5N), confirming that A2748 site m6A modification of the *NLRP3* mRNA is specifically mediated by METTL3-dependent activity. Collectively, these results indicate that METTL3-mediated targeting regulation of *NLRP3* mRNA stability occurs in an A2748 site m6A-dependent manner in HBx-expressing hepatocytes.

### HBx-induced B56α-interacting METTL3 increases *NLRP3* mRNA m6A levels to mediate pyroptosis in hepatocytes

**Fig. 5:**
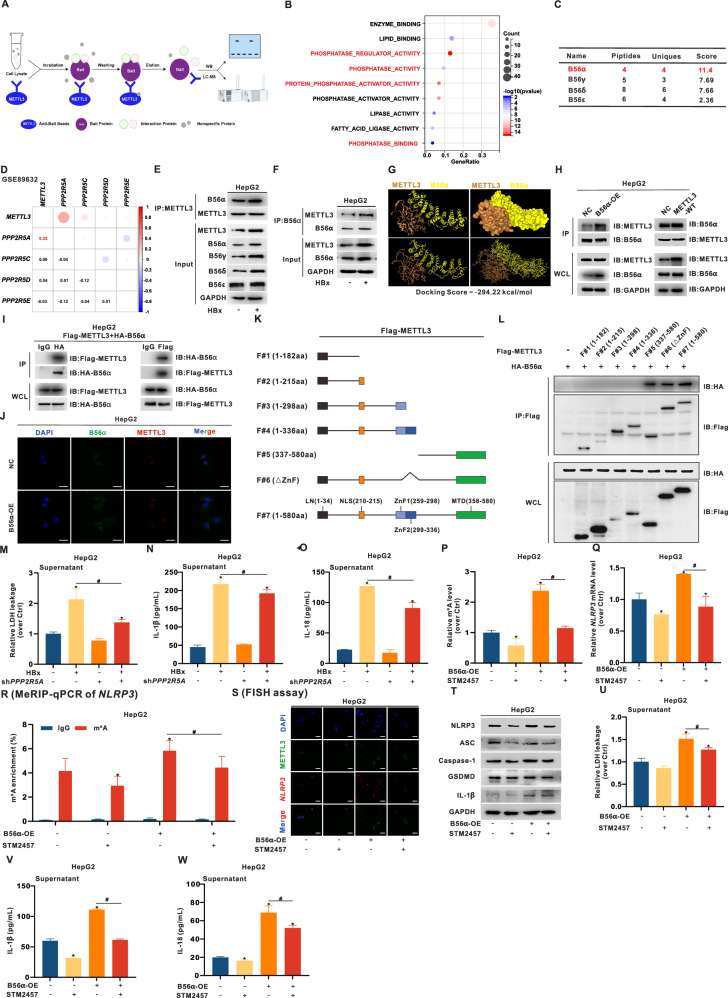
HBx-induced B56α-interacting METTL3 increases *NLRP3* mRNA m6A levels to mediate pyroptosis in HepG2 cells. **A**–**C** METTL3-overexpressing 293 T cells were constructed, followed by protein extraction and subjection to the Co-IP mass spectrometry (MS) assay (**A**). **B** GO enrichment analysis was performed on the identified METTL3-interacting proteins. **C** Potential protein interactions between PP2A B-subunits and METTL3 were identified. **D** The GSE89632 dataset, derived from NASH patients (*n* = 19) and healthy controls (HC, *n* = 24), was used for correlation analysis between *METTL3* and the four PP2A subunit genes. **E**, **F** Co-IP analyses were conducted using anti-METTL3 (**E**) and anti-B56α (**F**) antibodies to assess the binding interaction between METTL3 and B56α in HepG2 cells transfected with or without pcDNA3.1-HBx plasmid (1 μg/mL, 24 h). **G** Molecular docking images showing the predicted interaction between METTL3 and B56α using HDOCK (http://hdock.phys.hust.edu.cn/). **H** HepG2 cells overexpressing B56α (B56α-OE) or METTL3-WT were constructed. Co-IP analysis showing the binding interaction between endogenous METTL3 and B56α proteins. **I** HepG2 cells were co-transfected with Flag-METTL3 and HA-B56α recombinant plasmids for 24 h, followed by subjecting the cell extracts to Co-IP analysis. The binding interaction between exogenous Flag-METTL3 and HA-B56α fusion proteins was detected by WB with anti-HA and anti-Flag antibodies. **J** Representative images showing co-localization of B56α (green) and METTL3 (red) were captured by confocal microscopy in B56α-OE HepG2 cells, while DAPI (blue) was used to counterstain the nuclei. Scale bar, 10 µm. **K**, **L** Recombinant plasmids (based on PB513B-1 vector) expressing Flag-METTL3 fusion proteins with corresponding fragments, including Flag-METTL3-F#1 to -F#7 (**K**), were transfected into HepG2 cells (1 μg/mL, 24 h). **L** Co-IP analysis showing the binding interactions between the fragments of Flag-METTL3 fusion proteins and B56α protein. **M**–**O** HBx-expressing HepG2 cells were transfected with sh*PPP2R5A* (1 μg/mL, 24 h) to knock down B56α expression, while shNC was used as a negative control. The release levels of LDH (**M**), IL-1β (**N**), and IL-18 (**O**) in supernatants were detected. **P**–**W** B56α-OE HepG2 cells were treated with STM2457 (20 μM, 24 h) to inhibit m6A catalytic activity of METTL3. **P** Global RNA m6A levels in cells was measured. **Q** The level of *NLRP3* mRNA was detected by RT-qPCR. **R** *NLRP3* mRNA m6A levels were detected by MeRIP-qPCR using an anti-m6A antibody. **S** Representative FISH images showing co-localization of METTL3 (green) and *NLRP3* mRNA (red), while DAPI was used to counterstain the nuclei. Scale bar, 10 µm. **T** Expression levels of NLRP3, ASC, Caspase-1, GSDMD, and IL-1β proteins were detected by WB. **U**–**W** The release levels of LDH (**U**), IL-1β (**V**), and IL-18 (**W**) in supernatants were detected. Data are presented as mean ± SD, *n* = 3. *, *P* < 0.05, compared to the control group. #, *P* < 0.05, compared to the corresponding group.

Previous studies have focused on the regulation of METTL3-interacting proteins on its m6A enzymatic activity, subcellular localization, and nucleocytoplasmic shuttling [36]. To identify METTL3-associated proteins, we used co-immunoprecipitation followed by mass spectrometry (Co-IP/MS) analysis in METTL3-overexpressing 293 T cells (Fig. 5A). GO enrichment analysis of the METTL3 interactome revealed that METTL3-associated proteins were enriched predominantly in “phosphatase regulator activity”, “phosphatase activity”, “protein phosphatase activator activity”, and “phosphatase binding” pathways (Fig. 5B), suggesting that METTL3 may be subject to phosphorylation-mediated regulation. Among the identified candidates, four PP2A B-subunits—B56α, B56γ, B56δ, and B56ε—were highly represented, with elevated peptide counts and confidence scores (Fig. 5C). In parallel, PP2A phosphatase activity was significantly increased in HBx-expressing hepatocytes (S6A). Correlation analysis further revealed the strongest correlation (*r* = 0.33) between *METTL3* and *PPP2R5A* (encoding B56α) (Fig. 5D). Protein levels of B56α and B56γ were also significantly increased in HBx-expressing hepatocytes (Fig. S6B). Given that METTL3 is mainly localized to the nucleus [37], we detected the subcellular distribution of interacting proteins. B56α was strongly induced and translocated into the nucleus (Fig. S6C), and there was a significant interaction between METTL3 and B56α proteins in HBx-expressing hepatocytes (Fig. 5E, F).

To explore the regulatory role of B56α in METTL3 activity, we first performed molecular docking using HDOCK (http://hdock.phys.hust.edu.cn/), which predicted a structural interaction between the two proteins (Fig. 5G). We then established B56α-overexpressing (OE) hepatocytes (Fig. S6D, E) and validated the interactions between endogenous or exogenous METTL3 and B56α using Co-IP. In B56α-OE or METTL3-WT transfected cells, IP with anti-METTL3 or anti-B56α antibodies revealed protein interactions between METTL3 and B56α (Fig. 5H and S6F). Moreover, in hepatocytes co-transfected with Flag-METTL3 and HA-B56α, Co-IP with anti-Flag or anti-HA antibodies revealed the co-expression of HA-B56α and Flag-METTL3 in cells (Fig. 5I and S6G). IF analysis further revealed increased co-localization of B56α and METTL3, particularly in the nucleus (Fig. 5J), supporting their interaction upon HBx-induced B56α upregulation. To identify the METTL3 region responsible for B56α binding, we constructed a series of METTL3 truncation mutants—Flag-METTL3-F#1 to -F#7—covering key domains on basis of the PB513B-1 plasmid (Fig. 5K). Co-IP revealed that only truncations containing the MTD (F#5, F#6, and F#7) bound B56α (Fig. 5L). As the MTD is known to be the catalytic core of METTL3 for m6A activity [38], these results suggest that B56α interacts with METTL3 via its MTD to modulate m6A methyltransferase activity.

To investigate the role of B56α downregulation in HBx-induced pyroptosis, we used sh*PPP2R5A* to knock down B56α expression (Fig. S6H-I). This significantly reduced LDH, IL-1β, and IL-18 levels in supernatants of HBx-expressing hepatocytes (Fig. 5M-P and S6J-L), suggesting that downregulation of B56α reduces METTL3-mediated pyroptosis. Additionally, we employed STM2457, a novel, highly selective, orally active METTL3 inhibitor [39], to develop a pharmacologic intervention model. STM2457 treatment reduced NLRP3 protein levels in a dose-dependent manner (Fig. S6M). In B56α-OE cells, B56α increased global m6A levels, *NLRP3* mRNA expression, and m6A modification, whereas STM2457 reversed these effects (Fig. 5P-R and S6N), indicating that B56α regulates METTL3 activity toward the m6A modification of *NLRP3*. Moreover, STM2457 suppressed B56α-induced METTL3–*NLRP3* mRNA co-localization and attenuated NLRP3 inflammasome activation (Fig. 5S-T). Consequently, STM2457 treatment significantly reduced pyroptosis-related protein expression and LDH, IL-1β, and IL-18 release in B56α-OE hepatocytes (Fig. 5U-W and S6O-Q). These results indicate that HBx-induced B56α enhances METTL3 activity to promote *NLRP3* m6A modification and pyroptosis, while pharmacologic inhibition of METTL3 mitigates this inflammatory cascade.

### HBx-induced pyroptosis and lipotoxicity are suppressed by STM2457-mediated inhibition of METTL3 in HBx-expressing hepatocytes

**Fig. 6:**
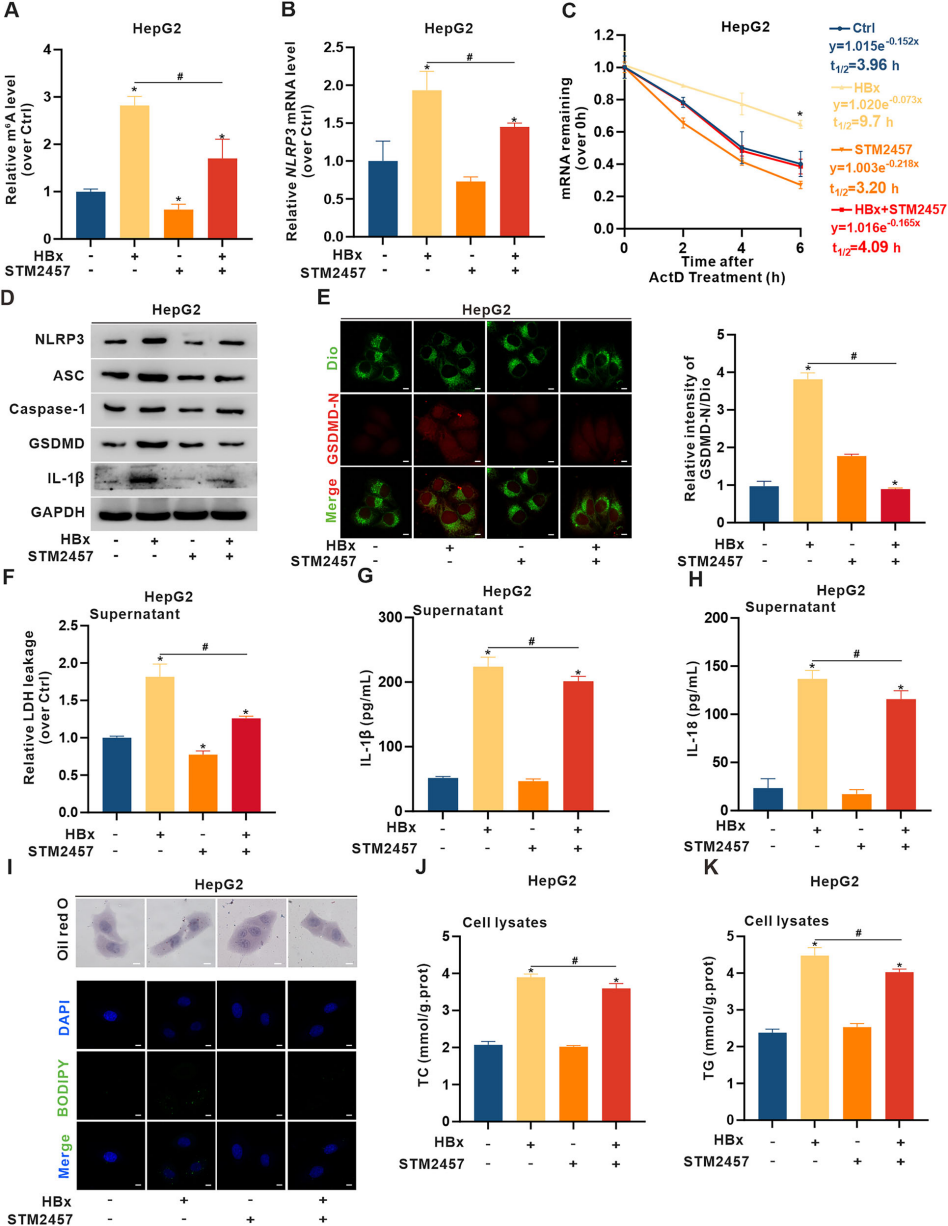
HBx-induced pyroptosis and lipotoxicity are suppressed by STM2457-mediated inhibition of METTL3 in HBx-expressing HepG2 cells. HBx-expressing HepG2 cells were treated with STM2457 (20 μM, 24 h). **A** Global RNA m6A levels in cells were measured. **B** *NLRP3* mRNA levels were detected by qRT-PCR. **C** The mRNA stability assay for half-life of *NLRP3* mRNA transcript was measured by qRT-PCR in cells treated with actinomycin D (ActD, 5 μg/ml) at the indicated time points. **D** Expression levels of NLRP3, ASC, Caspase-1, GSDMD, and IL-1β proteins were detected by WB. **E** Representative IF images showing co-localization of GSDMD-N (red) and Dio (green) were captured by confocal microscopy (Left). Scale bar, 10 µm. Quantification of the relative intensity of GSDMD-N/Dio is shown in a bar graph (Right). **F**–**H** The release levels of LDH (**F**), IL-1β (**G**), and IL-18 (**H**) in supernatants were detected. **I** Representative images showing Oil red O staining (Scale bar, 100 μm) and IF staining of LD (Scale bar, 10 μm) in cells. **J**, **K** The levels of TC (**J**) and TG (**K**) in cells were detected. Data are presented as mean ± SD, *n* = 3. *, *P* < 0.05, compared to the control group. #, *P* < 0.05, compared to the corresponding group.

Targeting the m6A-mediated regulation of NLRP3 expression may represent a novel therapeutic approach to suppress pyroptosis-associated hepatic injury. METTL3-targeted intervention was hypothesized to mitigate pyroptosis and lipotoxicity by disrupting m6A-modulated *NLRP3* mRNA stability. STM2457, a highly selective METTL3 inhibitor with no known off-target effects on other RNA methyltransferases [40], was used to evaluate this hypothesis in HBx-expressing hepatocytes. STM2457 treatment significantly alleviated the increase in global RNA m6A levels (Fig. 6A and S7A) and decreased *NLRP3* mRNA levels (Fig. 6B and S7B), suggesting impaired mRNA stability. To further verify this effect, we measured the half-life of *NLRP3* mRNA after transcriptional blockade using actinomycin D (ActD). Compared with that in control cells, *NLRP3* mRNA in HBx-expressing cells exhibited a prolonged half-life, whereas STM2457 treatment significantly shortened this half-life, even in the presence of HBx (Fig. 6C and S7C), confirming that METTL3-mediated m6A modification plays a key role in stabilizing *NLRP3* transcripts. Consistently, STM2457 also reduced the expression of pyroptosis markers, including NLRP3 and associated inflammasome proteins (Fig. 6D and S7D), diminished membrane co-localization of GSDMD-N (Fig. 6E and S7E), and decreased LDH, IL-1β, and IL-18 release (Fig. 6F-H and S7F-H). These findings indicate that METTL3 intervention can suppress NLRP3-dependent pyroptosis and related inflammation in HBx-expressing hepatocytes.

Given the critical role of m6A modifications in mRNAs and METTL3-mediated regulation of hepatic lipid metabolism [13], we further investigated the effect of METTL3 intervention on HBx-induced lipotoxicity related to MASH. The density and distribution of Oil red O stained LDs (Fig. 6I and S7I), as well as the quantity and area of BODIPY-labeled LDs (Fig. 6I and S7I), were increased in HBx-expressing cells, whereas STM2457 intervention counteracted these increases and decreased levels of TC and TG (Fig. 6J, K and S7J, K) in HBx-expressing hepatocytes. These results indicated that specific inhibition of METTL3 catalytic activity by STM2457 could relieve pyroptotic inflammation and lipotoxicity in HBx-expressing hepatocytes related to MASH phenotypes.

### METTL3 intervention by STM2457 alleviates NLRP3-dependent MASH in HBx-Tg mice in vivo

**Fig. 7:**
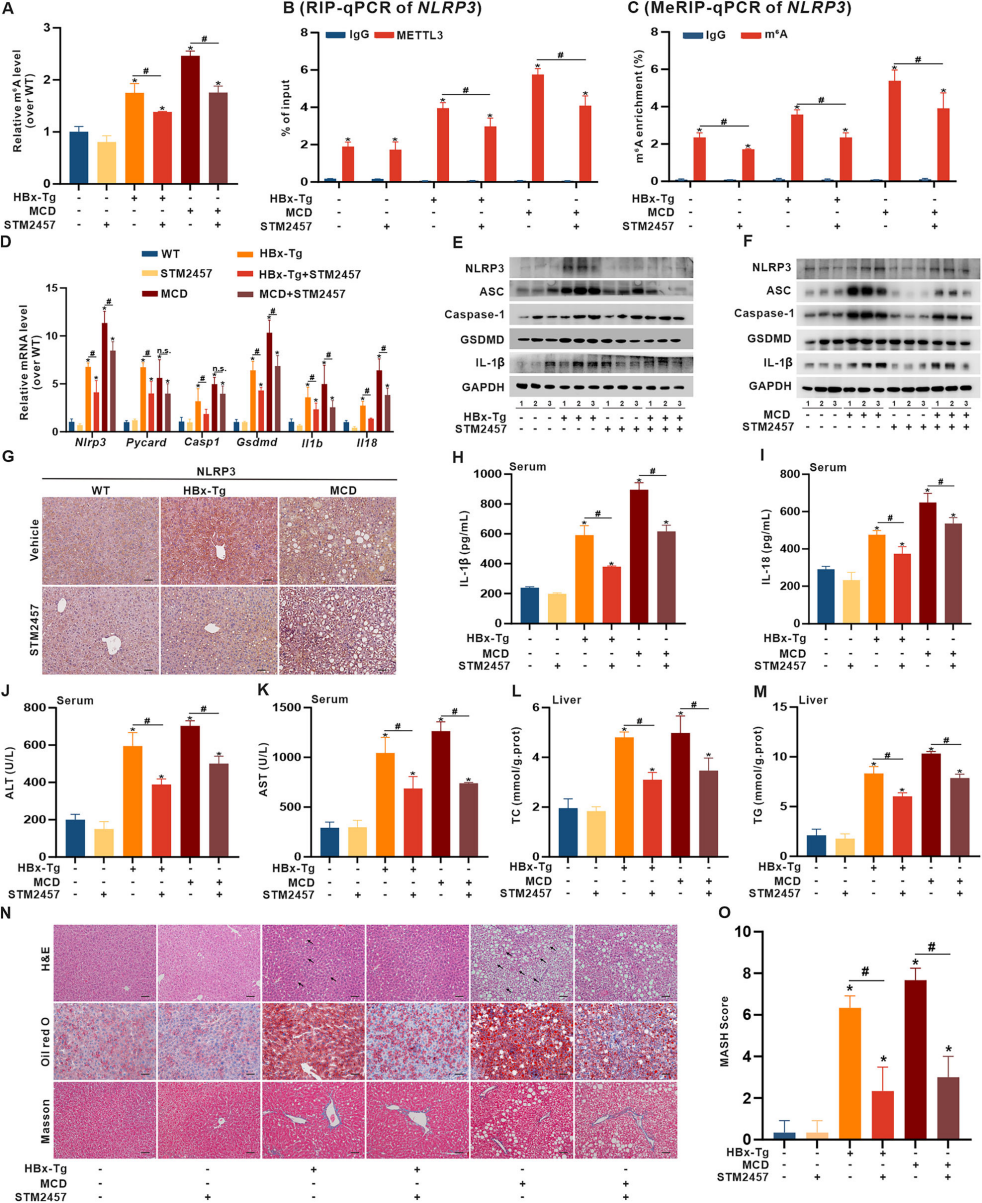
METTL3 intervention by STM2457 alleviates NLRP3-dependent MASH in HBx-Tg mice in vivo. HBx-Tg mice were used to construct the MASH model, and MCD-fed mice served as the positive model. Mice revived daily intraperitoneal injection of STM2457 (50 mg•kg^−1^ BW) for two weeks, while wild-type mice served as the negative control. n = 5 per group. **A** Global RNA m6A levels in livers were measured by colorimetric assays. **B** RIP-qPCR analysis of *NLRP3* mRNA in livers illustrating the binding interaction between *NLRP3* mRNA and METTL3 using anti-METTL3 antibody. **C** MeRIP-qPCR analysis of m6A levels on *NLRP3* mRNA in livers using anti-m6A antibody. **D** Relative mRNA levels of *NLRP3* and pyroptosis-related genes in livers were detected by qRT-PCR. **E**, **F** Expression levels of NLRP3, ASC, Caspase-1, GSDMD, and IL-1β proteins in livers were detected by WB. (**G**) Representative IHC staining images showing NLRP3 protein expression in liver tissues. Scale bar, 100 μm. **H**, **I** Levels of serum IL-1β (**H**) and IL-18 (**I**) were detected. **J**, **K** Levels of serum ALT (**J**) and AST (**K**) were detected. **L**, **M** Levels of TC (**L**) and TG (**M**) in livers were detected. **N** Representative images showing H&E, Oil red O, and Masson staining in liver tissues. Scale bar, 100 μm. Black arrows indicate inflammatory foci. **O** MASH score analysis is shown in a bar graph. Data are presented as mean ± SD, n = 3. *, *P* < 0.05, compared to the control group. #, *P* < 0.05, compared to the corresponding group.

Recent studies have focused on the inhibitory effects of STM2457 on the regulation of METTL3 in various diseases [39, 41]. To further investigate the role of METTL3 in regulating *NLRP3* mRNA and pyroptosis-related MASH in vivo, STM2457 (50 mg/kg) or the respective vehicle was administered daily i.p. injections for 14 days to HBx-related MASH model mice, with the MCD group serving as the MASH positive control (Fig. S8A). STM2457-mediated inhibition of METTL3 enzymatic activity was confirmed by reduced global RNA m6A levels in livers from HBx-Tg and MCD mice (Fig. 7A). Moreover, STM2457 intervention significantly reduced the binding affinity between METTL3 and *NLRP3* mRNA in the RIP assay (Fig. 7B) and reduced m6A modification of *NLRP3* mRNA in the MeRIP assay (Fig. 7C). Additionally, *NLRP3* mRNA levels were decreased in livers of HBx-Tg mice (Fig. 7D). Compared with those in the HBx-Tg or MCD groups, the hepatic mRNA levels of pyroptosis-related genes (including *Pycard*, *Casp1*, *Gsdmd*, *Il1b*, and *Il18*) (Fig. 7D), as well as the expression and distribution of NLRP3 and inflammasome-related pyroptosis markers (including GSDMD, ASC, Caspase-1, and IL-1β) (Fig. 7E-G and S8B), were significantly lower in livers from STM2457-treated HBx-Tg or MCD mice. To further validate the regulatory relevance of B56α in vivo, we examined its expression in liver tissues. Consistent with in vitro findings, B56α was significantly upregulated in HBx-Tg mice compared with wild-type controls (Fig. S8C). STM2457 treatment did not affect B56α protein levels, indicating that METTL3 inhibition does not directly affect B56α expression. However, co-immunoprecipitation revealed an enhanced B56α-METTL3 interaction in HBx-Tg livers, which was modestly reduced upon STM2457 treatment (Fig. S8D). This slight attenuation likely results from STM2457-induced conformational interference at the METTL3 MTD, the shared binding site for STM2457 and B56α. Moreover, STM2457 treatment significantly inhibited the increased levels of serum IL-1β, IL-18, and IL-6 in HBx-Tg and MCD groups (Fig. 7H-I and S8E).

Furthermore, STM2457 intervention significantly reduced elevated serum levels of ALT, AST, GPX, and ALP in HBx-Tg mice (Fig. 7J-K and S8F-G) and reduced hepatic TC, TG, and ox-LDL levels (Fig. 7L-M and S8H), indicating inhibition of liver lipotoxicity. Moreover, H&E, Oil red O, and Masson staining revealed severe steatohepatitis and fibrosis with increased MASH scores in liver tissues from HBx-Tg and MCD groups (Fig. 7N-O). STM2457 treatment alleviated hepatocyte ballooning, vacuolization, inflammatory foci, LD distribution, and MASH scores (Fig. 7N-O). These results indicate that targeting METTL3 inhibition by STM2457 can suppress lipid accumulation-related lipotoxicity and NLRP3 inflammasome-associated liver injury in HBx-driven MASH in vivo.

## Discussion

HBV is considered a “metabolic virus” that profoundly influences hepatic metabolic homeostasis. However, the relationships between chronic HBV infection and metabolic disorders—such as metabolic syndrome, diabetes, and MASH—remain poorly understood. The global prevalence of MASH has risen among the general population and patients with chronic hepatitis B (CHB), but its interaction with HBV and its clinical implications remain elusive. Numerous studies suggest that HBV infection may contribute to or accelerate MASH progression [42–44], although the underlying molecular mechanisms are still unclear. The prevailing view suggests that the HBx protein, encoded by the HBV genome, plays a key role in mediating metabolic changes following HBV infection. HBx has been shown to promote hepatic steatosis by enhancing lipid deposition and upregulating lipogenic enzymes [10, 45]. It also aggravates inflammation via NLRP3 inflammasome activation, partly through increased mitochondrial ROS production [24]. In this study, we provide direct evidence that HBV/HBx drives MASH-related lipotoxicity and inflammation. Using HBx-transgenic (HBx-Tg) mice, transcriptomic and metabolomic profiling revealed dysregulation of lipid- and inflammation-related pathways (e.g., *Fasn*, *Nlrp3*, linoleic acid, and stearic acid), suggesting HBx-induced metabolic disturbances. Following 8 weeks of MCD diet feeding, HBx-Tg mice developed enhanced lipid accumulation and NLRP3 inflammasome activation, with histopathology showing features consistent with those of MASH, including steatosis, inflammation, and ballooning degeneration. Consistent with these findings, HBx-expressing hepatocyte models presented similar phenotypes in vitro. These findings demonstrate that HBx disrupts hepatic metabolism and promotes MASH progression by impairing lipid homeostasis and activating inflammatory pathways.

The mechanisms underlying HBx-related liver lipotoxicity and inflammation in MASH are multifactorial. Recently, m6A RNA methylation has garnered attention for its role as a key regulator in various diseases. In MASH, m6A modification contributes to driving disease progression by regulating lipid accumulation, inflammation, and fibrosis [46, 47]. It exerts both direct and indirect effects on gene expression, including modulation of lipid metabolism via *ACLY* and *SCD1* [15]. In our study, HBx promoted lipid accumulation and activated the NLRP3 inflammasome. Transcriptomic and experimental data from HBx-Tg mice revealed notable alterations in m6A-related regulators, particularly METTL3 and METTL14. Additionally, transcriptome datasets from patients with NASH corroborate these findings, highlighting the central role of METTL3 in lipid metabolism and inflammation. In vitro, HBx increased global RNA m6A levels in parallel with increased METTL3 expression and activity. Mechanistically, METTL3 directly binds the m6A motif on *NLRP3* mRNA, increasing its stability and inducing pyroptosis. We specifically identified A2748 site in the *NLRP3* CDS as the critical functional m6A modification target of METTL3.

m6A methylation is crucial for regulating RNA metabolism, yet upstream regulation of METTL3 remains less defined. Post-translational modifications (PTMs), such as SUMOylation [48], lactylation [38], acetylation [49], and phosphorylation [50], have been implicated in modulating METTL3 localization, stability, and activity. Among these, phosphorylation has been extensively studied, with known sites including S2, S43, S48, S50, S219, and S243 [50]. In this study, mass spectrometry screen following METTL3 Co-IP, PP2A-B56α emerged as a prominent interacting protein of METTL3. Previous research has shown that HBx interacts with the PP2A-C subunit in liver cancer cells [51], and our group previously reported that HBx induces cell cycle arrest and apoptosis via PP2A-B56γ-mediated dephosphorylation of p-Thr55-p53 and activation of the p21 pathway [52]. Although PP2A-B56γ was also identified via METTL3 Co-IP analysis, PP2A-B56α displayed a higher confidence score and stronger association with METTL3, prompting us to focus on the regulatory role of PP2A-B56α on METTL3. Notably, PP2A-B56α did not alter METTL3 phosphorylation, but increased total m6A levels, likely by increasing METTL3 abundance and facilitating its nuclear translocation. Domain-mapping analysis revealed that B56α binds the METTL3 MTD—critical for catalytic activity—thereby enhancing METTL3-mediated m6A modification and NLRP3-dependent pyroptosis. This B56α/METTL3-NLRP3 axis uncovers a novel mechanism by which HBx promotes MASH progression through m6A-dependent NLRP3 activation and inflammatory signaling.

Although our study offers compelling mechanistic insights, several limitations remain. Functional validation in human liver tissues or primary hepatocytes is required to facilitate clinical translation. Furthermore, while *NLRP3* mRNA was the primary target explored, additional m6A-regulated transcripts involved in fibrosis and lipid homeostasis also warrant investigation. Time-course studies are necessary to elucidate the temporal dynamics of METTL3 activity during MASH progression. Finally, although we characterized B56α as a METTL3 regulator, the full spectrum of its PTMs and regulatory influence requires further clarification.

## Conclusions

In this study, we demonstrated that HBV/HBx induces METTL3 to regulate *NLRP3*^A2748^ mRNA stability via m6A modification, thereby promoting hepatic pyroptosis. Our results indicate that METTL3 interacts with PP2A-B56α to mediate its methyltransferase activity and regulate NLRP3-dependent pyroptosis in HBx-related MASH. These results reveal a novel mechanism in which the B56α/METTL3-NLRP3 axis regulates HBx-related MASH, highlighting METTL3 as a potential therapeutic target for preventing MASH pathogenesis.

## Supplementary information

Supplemental Material

Original Data

## Data Availability

All data supporting this study’s findings are available within the article and its supplementary materials. Researchers and interested parties can access the relevant information and supporting evidence from the corresponding authors upon reasonable request.
