# Supplementary material for: Hepatic steatosis and pyroptosis are induced by the hepatitis B virus X protein via B56α-METTL3 interaction-mediated m6A modification of the NLRP3 mRNA
Source: Cell Death Dis. 2025 Oct 6;16(1):698. doi: 10.1038/s41419-025-08019-8 (PMC12500908; doi:10.1038/s41419-025-08019-8)
Supplement: Supplementary file 1 — Supplemental Material [file 41419_2025_8019_MOESM1_ESM.pdf]

Supplemental Material

Supplementary Materials and Methods

Multi-omics and bioinformatics analysis

The transcriptomic and untargeted metabolomic analyses, including data generation and processing for liver samples from HBx-Tg mice (n = 4) and wild type (WT) mice (n = 4), were performed as described previously (1). Briefly, for transcriptomic analysis, standardization and expression value calculation were performed on the original dataset. The log2 fold change (logFC $\geq$ 1) and *P*-values ($P \leq 0.05$) were used to screen differentially expressed genes (DEGs). The relative expression levels of inflammation-, lipid metabolism-, and m6A-related genes in liver tissue were analyzed, and DEGs were visualized using heatmaps and volcano plots. Gene ontology (GO) pathway enrichment analysis was subsequently performed to identify pathways significantly affected by HBx expression. For untargeted metabolomic analysis, metabolites identified by liquid chromatography–mass spectrometry (LC-MS) were matched against the mzCloud database based on reference standards, and features with coefficient of variation below 30% in quality control samples were retained. Differentially expressed metabolites (DEMs) were filtered using multivariate statistical methods, including principal component analysis (PCA) and partial least-squares discriminant analysis (PLS-DA). The raw metabolomic data were normalized and visualized as volcano plots, heatmaps, and bubble plots. For integrated transcriptome–metabolome analysis, DEGs and DEMs were jointly analyzed using the Kyoto Encyclopedia of Genes and Genomes (KEGG) database. Lipid- and inflammation-related DEGs and their associated DEMs were mapped to relevant metabolic pathways, followed by correlation analysis.

The publicly available NASH associated dataset (GSE89632) of mRNA expression was retrieved from the Gene Expression Omnibus (GEO) database. The GSE89632 dataset comprises samples derived from 24 healthy living liver donors and 19 NASH liver donors. The relative expressions of m6A-related genes in liver tissues were analyzed. Heatmap and volcano plots were generated to visualize DEGs. Gene set enrichment analysis (GSEA) was performed to determine various signaling signatures related to NASH, and the pathways with a false discovery rate (FDR) < 0.25 were considered significantly enriched.

Animal experiment design and treatment

C57BL/6-WT and C57BL/6-HBx-Tg mice (20-25 g, 6-month-old, male) were randomly divided into 6 groups (n = 5 per group): (1) the control group (WT), treated with normal saline; (2) the HBx group, treated with normal saline; (3) the MCD group, fed with a methionine choline deficiency (MCD) diet for 8 weeks to induce MASH model, as the positive control; (4) the STM2457 group, treated daily intraperitoneal (i.p.) injection with STM2457 (50 mg•kg^{-1} BW; Sigma-Aldrich) for two weeks (2). (5) the STM2457-interventive MCD group (MCD+STM), MCD-fed mice receiving daily intraperitoneal (i.p.) injection with STM2457; (6) the STM2457-interventive HBx group (HBx+STM), HBx-Tg mice receiving daily intraperitoneal (i.p.) injection with STM2457. Sample size (n = 5 per group) was determined from pilot studies using similar models and interventions, showing sufficient power to detect biologically relevant differences (1). Inclusion criteria: male, 6 months old, healthy on arrival; animals with illness or abnormal behavior were excluded prior to randomization. No animals were excluded post-treatment. After treatment, mice were euthanized and serum and liver tissues were collected for subsequent analyses. Investigators performing histology and biochemistry were blinded to group allocation. All animal experiments were approved by the Ethical Committee for Animal Experimentation at Xiamen University (Ethics Approval No. XMULAC20220282, dated 2022-03-12).

Histopathological assay of liver

Paraffin-embedded mouse liver tissues were sliced into 5 μm sections and subjected to hematoxylin and eosin (H&E) and Masson staining. Fresh liver tissues were embedded in optimal cutting temperature (OCT) gel, cryosectioned, and stained with Oil red O for lipid droplets (LDs) detection. LDs in the liver were detected according to the instructions of the kit (Nanjing Jiancheng; Nanjing, Jiangsu, China). Immunohistochemistry (IHC) staining was performed using IHC kit (Maxim; Fuzhou, Fujian, China) as previously described (3). Expression of the indicated proteins was detected by incubating sections with primary antibodies. Images were captured with a light microscope (Nikon; Tokyo, Japan). The detailed information on the primary antibodies, including the manufacturers, code number, dilution used in IHC, and species, is listed in Supplementary Table S1.

Biochemical analysis of serum and liver

Serum alanine transaminase (ALT), aspartate aminotransferase (AST), and interleukin 6 (IL-6) levels, along with hepatic total cholesterol (TC), triglyceride (TG), oxidized low density lipoprotein (ox-LDL), alkaline phosphatase (ALP), and glutathione peroxidase (GPx) contents, were measured using commercial kits (Nanjing Jiancheng) according to the manufacturer's instructions.

Enzyme-linked immunosorbent assay (ELISA) of IL-1β and IL-18

ELISA was used to quantify the levels of inflammatory factors IL-1β and IL-18 in mouse serum and cell culture supernatants using commercial kits (Abclonal; Wuhan, Hubei, China) according to the manufacturer's instructions.

Cell culture

HepG2 and HepaRG cells were obtained from the American Type Culture Collection (ATCC, Beijing, China). HepG2 cells were maintained in Dulbecco's Modified Eagle's Medium (DMEM) supplemented with 10% fetal bovine serum (FBS) and 1% penicillin-streptomycin at 37°C in 5% CO_2. HepaRG cells differentiation and culture were performed as described previously (4). All cell lines were authenticated by STR profiling and confirmed mycoplasma-free prior to use. HepG2 and HepaRG cells were transfected with pcDNA3.1-*HBx* for 6 h. For NLRP3 or METTL3 intervention, the inhibitors CY09 (10 μM; Sigma-Aldrich) or STM2457 (20 μM) was added to the medium for 24 h.

Plasmids, transfection, and RNA interference

Human *METTL3*-coding sequence (CDS) and *PPP2R5A*-CDS expression plasmids were constructed by inserting the full-length open reading frames of the *Homo sapiens METTL3* and *PPP2R5A* mRNA into the PiggyBac Dual promoter vector (PB513B-1) (Miaolingbio; Wuhan, Hubei, China). The *METTL3* mutant (MUT) of the catalytic domain (395–398 aa, DPPW to APPA) was generated using the KOD-Plus Mutagenesis Kit (TOYOBO; Osaka, Kansai, Japan). Truncated *METTL3*-CDS plasmids encoding 1-182, 1-215, 1-296, 1-336, 336-580 aa, and ΔZnF (Δ296-336 aa) were constructed based on the structural domains of *METTL3* mRNA (5). Stable overexpression (OE) cells, including METTL3 (HepG2-*METTL3*^{OE}) and its mutant (HepG2-*METTL3*^{MUT}) and B56α (HepG2-*PPP2R5A*), were constructed by co-transfecting the corresponding PB513B-1 plasmids with super piggybac transposase (PB200PA-1, Miaolingbio) using Lipofectamine 2000 (Thermo Fisher; Waltham, MA, USA)

according to the manufacturer's instructions.

Stable knockdown of proteins was accomplished using the pLKO.1-puromycin short hairpin RNA (shRNA) system (6). ShRNAs targeting *METTL3, PPP2R5A*, and a non-targeting sequence (as the negative control, NC) were obtained from Sigma-Aldrich (St. Louis, MO, USA). Cells with knockdown of human METTL3 (HepG2-sh*METTL3*) and B56α (HepG2-sh*PPP2R5A*) were constructed with psPAX2, pMD2.G (Miaolingbio), and Lipofectamine 2000 according to the manufacturer's instructions, while cells were screened with 5 μg/ml puromycin. All the primers are listed in Supplementary Table S2.

Quantification of global RNA m6A levels

The m6A levels in RNA were determined using the EpiQuick^{TM} RNA Methylation Quantification Kit (Epigentek; Farmingdale, NY, USA) according to the manufacturer's protocol. Briefly, 200 ng of RNA was immobilized in microplate wells and incubated with binding solution, followed by sequential incubations with a specific anti-m6A capture antibody and a detection antibody. m6A levels were quantified via colorimetric detection at 450 nm. The relative level of m6A was calculated: m6A % = [(OD sample－OD negative control)/S] / [(OD positive control－OD negative control)/P] × 100%, where S is the RNA amount (ng) of the sample and P is the RNA amount (ng) of the positive control (7).

RNA immunoprecipitation-qPCR (RIP-qPCR) of *NLRP3* mRNA

The RIP-qPCR assay was performed using a RIP kit (BersinBio; Guangzhou, Guangdong, China). Briefly, cells and homogenized liver tissues were lysed in RIP lysis buffer supplemented with RNase inhibitor and protease inhibitor cocktail. Anti-METTL3 antibody was pre-incubated with protein A/G magnetic beads, while IgG served as a negative control. The cleared RIP lysate supernatant was added to the antibody–bead complex in RIP buffer, incubated to form RNA-binding protein-RNA complexes, washed with RIP washing buffer, and treated with proteinase K. The eluted RNA was extracted, reverse-transcribed into cDNA, and subjected to quantitative real-time PCR (qRT-PCR) for *NLRP3* mRNA, using primers listed in Supplementary Table S3.

Methylated RNA immunoprecipitation-qPCR (MeRIP-qPCR) for m6A on *NLRP3* mRNA

Enrichment of m6A-modified *NLRP3* mRNA fragments was performed using the EpiQuick m6A RNA Enrichment MeRIP Kit (Epigentek) followed by qRT-PCR, according to

the manufacturer's protocol. Briefly, total RNA was extracted using TRIzol reagent, subjected to fragmentation, and incubated with an anti-m6A antibody (Abcam; Cambridge, MA, USA) bound to affinity beads. After treatment with Nuclear Digestion Enhancer and Cleavage Enzyme Mix (Epigentek), enriched RNA was recovered following proteinase K digestion and purification with the RNA Purification Solution. One-tenth of the input RNA served as a loading control. m6A enrichment was quantified by qRT-PCR for *NLRP3* mRNA and normalized to the input sample.

RNA pulldown

A biotin-labeled oligonucleotide probe of *NLRP3* mRNA was commercially synthesized (Genepharma; Shanghai, China) and incubated with streptavidin magnetic beads (New England Biolabs; Beverly, MA, USA). Briefly, after being bound, the *NLRP3* probe-bead complex was incubated with whole-cell lysates. After washing, METTL3 protein was pulled down and detected by Western blotting (WB). Probe sequences are listed in Supplementary Table S4.

Luciferase reporter assay

To assess METTL3-mediated regulation of *NLRP3* mRNA transcription, a luciferase reporter assay was performed using the Dual-Luciferase Reporter Assay System (Promega; Madison, WI, USA) according to the manufacturer's protocol. Wild type (WT) human *NLRP3*-5′UTR, -CDS, and -3′UTR luciferase reporter plasmids were constructed by amplifying the corresponding mRNA regions and cloning them into the pmirGLO vector. Mutant (MUT) reporter *NLRP3*-2206(A-C), -2706(A-C), and -2748(A-C) were generated using the KOD-Plus Mutagenesis Kit (TOYOBO). HepG2 and HepaRG cells were transfected with WT or mutant pmirGLO-*NLRP3* plasmids. Firefly and Renilla luciferase activities were detected. Results were expressed as relative Firefly/Renilla luciferase ratios. All primers are listed in Supplementary Table S5.

***NLRP3* mRNA stability**

The transcriptional inhibitor actinomycin D (ActD; MedChem Express; Monmouth Junction, NJ, USA) was used to block RNA synthesis for mRNA stability assays (8). HepG2 and HepaRG cells were transfected with PB-$METTL3^{OE}$, PB-$METTL3^{MUT}$, or sh*METTL3* plasmids for 48 h, then treated with 5 μg/mL ActD for 2, 4, and 6 h. Total RNA was extracted, and residual *NLRP3* mRNA levels were quantified by qRT-PCR.

Fluorescence in situ hybridization (FISH) assay

The subcellular distribution and expression of *NLRP3* mRNA were assessed using a FISH kit (GenePharma) according to the manufacturer's protocol. Briefly, HepG2 and HepaRG cells were fixed in 4 % paraformaldehyde and hybridized with 10 nM Cy5-labeled *NLRP3* probe (GenePharma) in a dark chamber. After washing, nuclei were counterstained with DAPI. Images were captured using a high-sensitivity laser confocal microscope (LSM 880, Zeiss; Jena, Germany). Probe sequences are listed in Supplementary Table S6.

Cell viability assay

Cell viability was determined using the Cell Counting Kit-8 (CCK-8, Abclonal) as described previously (9). Briefly, HepG2 and HepaRG cells were seeded into 96-well plates at 5,000 cells per well. transfected with pcDNA3.1-*HBx* for 6 h, and incubated with CCK-8 reagent for 2 h. Absorbance at 450 nm (OD_{450}) was measured using a multifunctional microplate reader (BMG LabTech; Ortenberg, Germany).

Measurement of lactic dehydrogenase (LDH) leakage

Cell membrane permeability was assessed using an LDH assay kit (Beyotime; Shanghai, China) (10). Culture supernatants were collected to measuree LDH release from HepG2 and HepaRG cells in the treatment groups.

Caspase-1 activity detection

the corresponding experimental design, At the experimental endpoint, culture supernatants were collected and Caspase-1 activity was measured using a commercial kit (Proteintech; Wuhan, Hubei, China) according to the manufacturer's instructions.

Flow cytometry (FCM) analysis for pyroptotic cells

Cells were stained with FLICA 660-YVAD-FMK and propidium iodide (PI) to identify cells with membrane pores, according to manufacturer's instructions (FLICA 660 In Vitro Active Caspase-1 Detection Kit, ImmunoChemistry Technologies; Bloomington, MN, USA). HepG2 and HepaRG cells were incubated with 30 × FLICA 660 for 1 h, washed with 1 × Pyroptosis Wash Buffer, and subsequent stained with PI. The percentage of pyroptotic cells was determined using a CytoFLEX flow cytometer (Beckman Coulter; Krefeld, Germany).

Immunofluorescence (IF) assay

IF analysis was performed as described previously (11). Detailed information on the

antibodies, including those against GSDMD and METTL3, are listed in Supplementary Table S1.

Co-immunoprecipitation (CO-IP) and CO-IP-based mass spectrometry (CO-IP-MS)

The CO-IP assay was performed according to the operating protocols. HepG2 and HepaRG cells were lysed in RIPA buffer containing protease inhibitors on ice. The lysates were centrifuged at 10,000 × g at 4°C. The supernatants were collected and incubated with anti-METTL3, anti-B56α, or control IgG antibodies. Protein A/G magnetic beads were then added, and the mixtures were incubated for 1 h at 4°C. After washing, the bound proteins were eluted and analyzed by WB to detect METTL3 or B56α protein.

To identify proteins interacting with METTL3, 293T cells overexpressing METTL3 (293T-*METTL3*^{OE}) were constructed. Protein samples eluated from CO-IP were either analyzed directly by WB or resolved by SDS-PAGE and stained with Coomassie blue. Specific bands were excised and subjected to in-gel tryptic digestion followed by LC-MS/MS analysis. LC–MS/MS was performed on an Orbitrap Fusion Lumos mass spectrometer (Thermo Fisher).

qRT-PCR

qRT-PCR analysis was performed as previously described (12). All primers used in this study are listed in Supplementary Table S3.

Western blotting (WB)

WB analysis was performed as previously described (12). Detailed information on the primary antibodies, including manufacturers, catalog number, species, and dilutions used for WB, RIP, and IHC, is listed in Supplementary Table S1.

Statistical analysis

All experiments were carried out in triplicate, and statistical parameters, including sample size and significance analysis, are specified in the figure legends. Data obtained from at least three independent experiments were analyzed using GraphPad Prism software (version 9; La Jolla, CA, USA). Quantitative data are presented as the mean ± standard deviation (SD). Prior to statistical comparison, the homogeneity of variance across groups was assessed using Levene's test. Differences between two groups were compared with an unpaired two-tailed Student's *t* test. Comparisons among multiple groups were performed using one-way ANOVA

followed by the least significant difference (LSD) post-hoc test for pairwise comparisons. A P value < 0.05 was considered statistically significant, while * and # denote comparisons with the corresponding group with $P < 0.05$.

Supplementary References

1. Lan Y, Qian B, Huang HY, Wang P, Li T, Yuan Q, et al. Hepatocyte-Derived Prostaglandin E2-Modulated Macrophage M1-Type Polarization via mTOR-NPC1 Axis-Regulated Cholesterol Transport from Lysosomes to the Endoplasmic Reticulum in Hepatitis B Virus x Protein-Related Nonalcoholic Steatohepatitis. Int J Mol Sci. 2022;23(19).
2. Yankova E, Blackaby W, Albertella M, Rak J, De Braekeleer E, Tsagkogeorga G, et al. Small-molecule inhibition of METTL3 as a strategy against myeloid leukaemia. Nature. 2021;593(7860):597-601.
3. Zhang S, Che L, He C, Huang J, Guo N, Shi J, et al. Drp1 and RB interaction to mediate mitochondria-dependent necroptosis induced by cadmium in hepatocytes. Cell Death Dis. 2019;10(7):523.
4. Chen YY, Lin Y, Han PY, Jiang S, Che L, He CY, et al. HBx combined with AFB1 triggers hepatic steatosis via COX-2-mediated necrosome formation and mitochondrial dynamics disorder. J Cell Mol Med. 2019;23(9):5920-33.
5. Oerum S, Meynier V, Catala M, Tisné C. A comprehensive review of m6A/m6Am RNA methyltransferase structures. Nucleic Acids Res. 2021;49(13):7239-55.
6. Che L, Du ZB, Wang WH, Wu JS, Han T, Chen YY, et al. Intracellular antibody targeting HBx suppresses invasion and metastasis in hepatitis B virus-related hepatocarcinogenesis via protein phosphatase 2A-B56γ-mediated dephosphorylation of protein kinase B. Cell proliferation. 2022;55(11):e13304.
7. Yu H, Liu J, Bu X, Ma Z, Yao Y, Li J, et al. Targeting METTL3 reprograms the tumor microenvironment to improve cancer immunotherapy. Cell chemical biology. 2023.
8. Zhao T, Sun D, Long K, Xiong W, Man J, Zhang Q, et al. N(6)-methyladenosine promotes aberrant redox homeostasis required for arsenic carcinogenesis by controlling the adaptation of key antioxidant enzymes. Journal of hazardous materials. 2023;465:133329.
9. Lin JX, Xu CY, Wu XM, Che L, Li TY, Mo SM, et al. Rab7a-mTORC1 signaling-mediated cholesterol trafficking from the lysosome to mitochondria ameliorates hepatic lipotoxicity induced by aflatoxin B1 exposure. Chemosphere. 2023;320:138071.
10. Zhang LY, Zhan DL, Chen YY, Wang WH, He CY, Lin Y, et al. Aflatoxin B1 enhances pyroptosis of hepatocytes and activation of Kupffer cells to promote liver inflammatory injury via dephosphorylation of cyclooxygenase-2: an in vitro, ex vivo and in vivo study. Arch Toxicol. 2019;93(11):3305-20.
11. Che L, Wu JS, Xu CY, Cai YX, Lin JX, Du ZB, et al. Protein phosphatase 2A-B56γ-Drp1-Rab7 signaling axis regulates mitochondria-lysosome crosstalk to sensitize the anti-cancer therapy of hepatocellular carcinoma. Biochem Pharmacol. 2022;202:115132.
12. Zhuang Q, Zhou T, He C, Zhang S, Qiu Y, Luo B, et al. Protein phosphatase 2A-B55δ enhances chemotherapy sensitivity of human hepatocellular carcinoma under the regulation of microRNA-133b. J Exp Clin Cancer Res. 2016;35:67.

Supplementary figures and legends

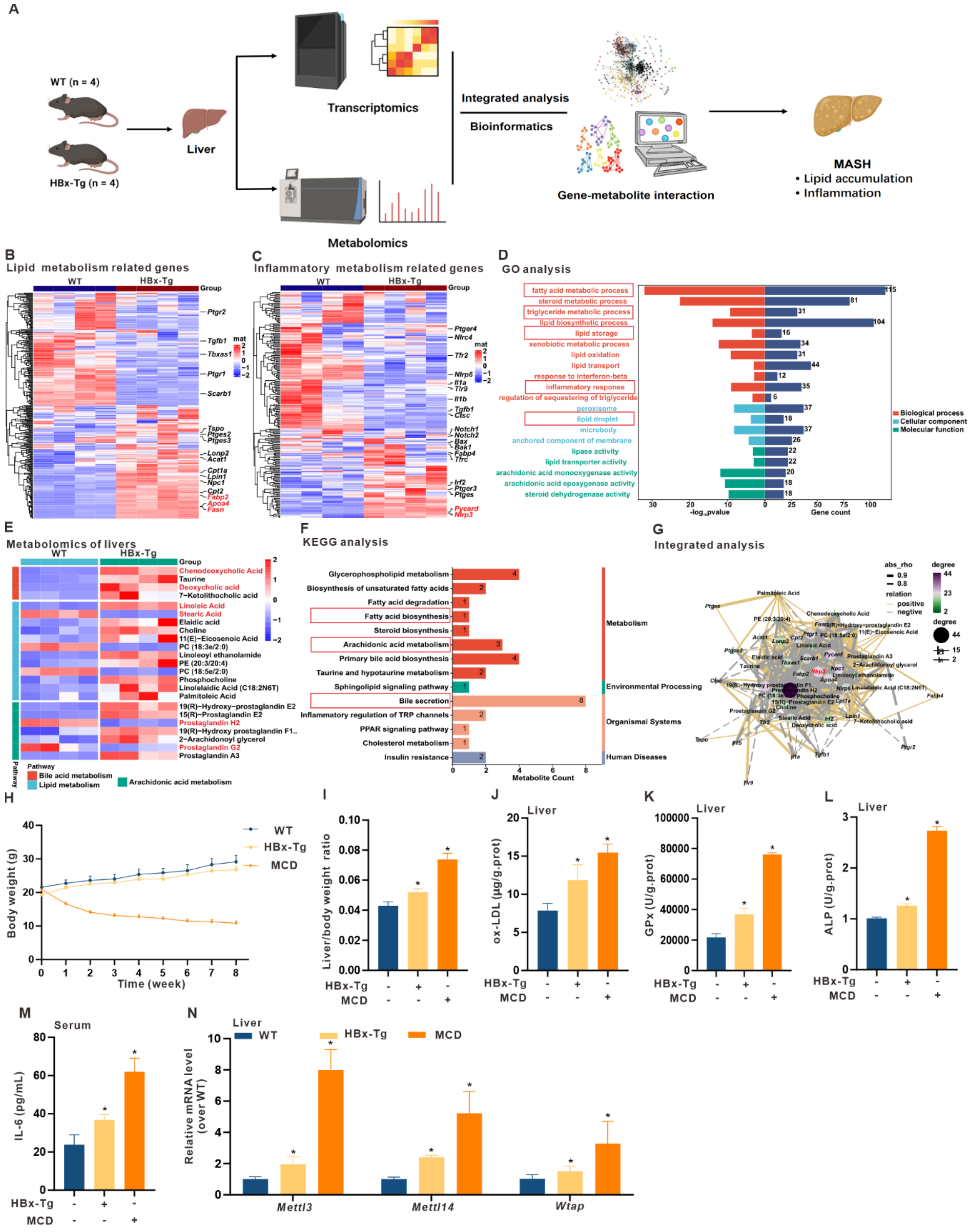

Fig. S1. m6A modification is associated with lipotoxicity and NLRP3-mediated inflammation in HBx-related MASH in transgenic mice. (A-G) Transcriptomic profiling was performed on liver tissues from WT mice (n = 4) and HBx-Tg mice (n = 4). (A) Schematic of the correlation analysis between gene expression data and metabolomics. (B-C) Heatmap of lipid- (B) and inflammatory- (C) metabolism-related DEGs. (D)

GO enrichment analysis of DEGs is shown. (E) Heatmap of arachidonic acid, lipid and bile acid metabolism-related DEMs. (F) KEGG enrichment analysis of DEMs is shown. (G) Integrated metabonomic–transcriptomic analysis of lipid- and inflammation-related genes and metabolites. (H-N) HBx-Tg mice were used to construct the MASH model, whereas C57BL/6 WT mice served as the negative control and MCD-fed mice as the positive model. n = 5 per group. (H) Body-weight growth curve. (I) Liver/body weight ratio. (J-M) Hepatic levels of ox-LDL (J), GPx (K), and ALP (L), and serum levels of IL-6 (M). (N) Relative mRNA levels of *Mettl3*, *Mettl14*, and *Wtap* were measured by qRT-PCR. Data are presented as mean ± SD. *, $P < 0.05$, compared to the WT group.

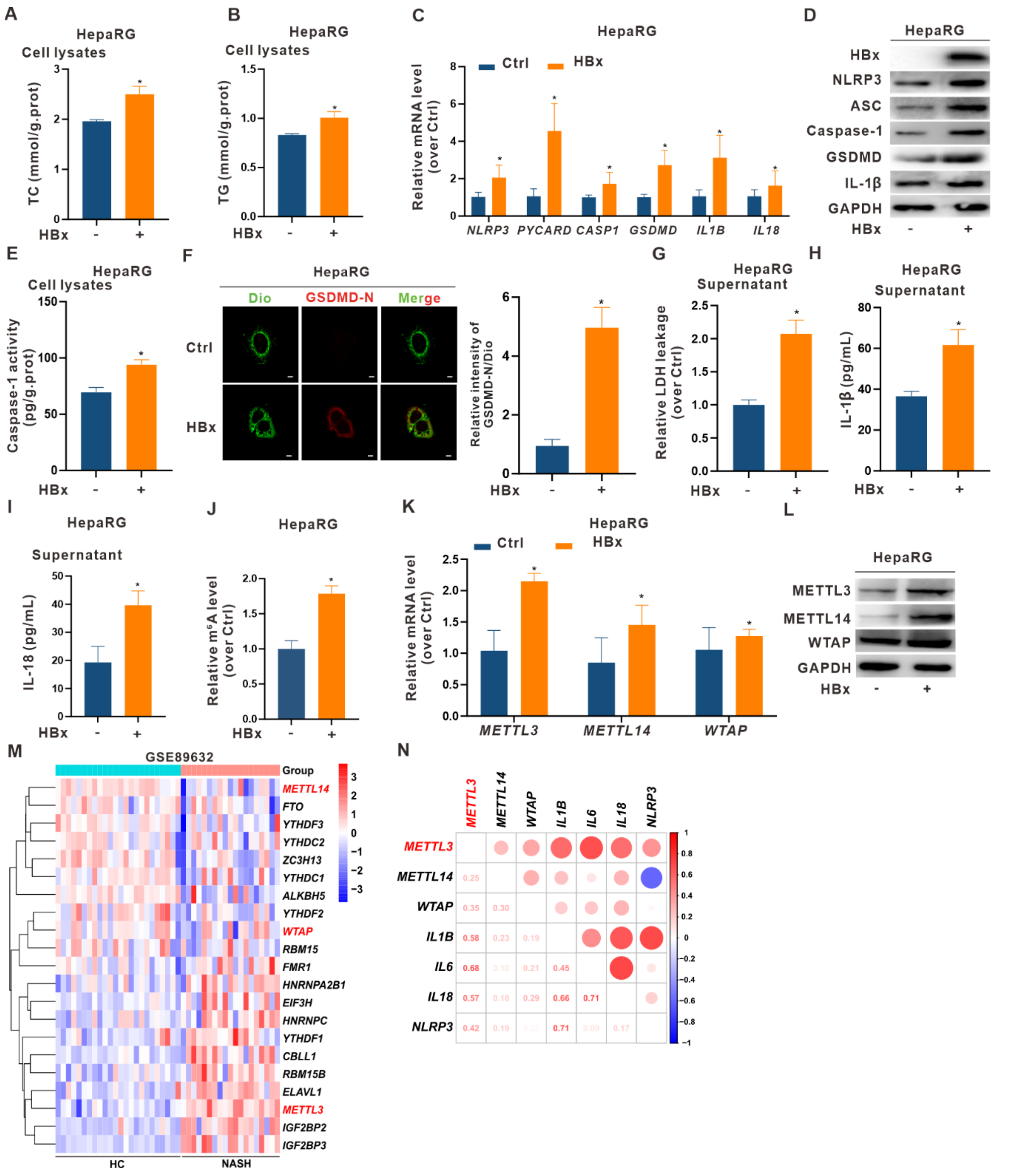

Fig. S2. HBx upregulates NLRP3-mediated pyroptosis and MASH-associated METTL3 in HBx-expressing hepatocytes *in vitro*. (A-L) HepaRG cells were transfected with pcDNA3.1-HBx (1 μg/ml, 24 h) to construct HBx-expressing hepatocytes, while the negative control (NC) cells transfected with pcDNA3.1 vector served as controls. (A-B) Levels of TC (A) and TG (B) in cells. (C) Relative mRNA levels of *NLRP3* and pyroptosis-related genes were measured by qRT-PCR. (D) Levels of HBx expression and NLRP3, ASC, Caspase-1, GSDMD, and IL-1β proteins were detected by WB. (E) The activity of caspase-1 related to pyroptosis was quantified by ELISA. (F) Representative images showing co-localization of GSDMD-N (red) and Dio (green) were captured by confocal microscopy (Left). Scale bar, 10 μm. Quantification of the relative

intensity of GSDMD-N/Dio is shown in a bar graph (Right). (G-I) Levels of LDH (G), IL-1β (H), and IL-18 (I) in supernatants. (J) Global RNA m6A levels in cells were measured by colorimetric assays. (K) Relative mRNA levels of *METTL3*, *METTL14*, and *WTAP* were detected by qRT-PCR. (L) Expression levels of METTL3, METTL14, and WTAP proteins were detected by WB. (M-N) A liver transcriptome dataset (GSE89632) was derived from NASH patients (n = 19) and healthy controls (HC, n = 24). (M) The heatmap of m6A modification-related DEGs. (N) A correlation heatmap was generated to illustrate the relationships between DEGs associated with m6A regulatory factors and those related to pyroptosis. Data are presented as mean ± SD. *, $P < 0.05$, compared to the control group.

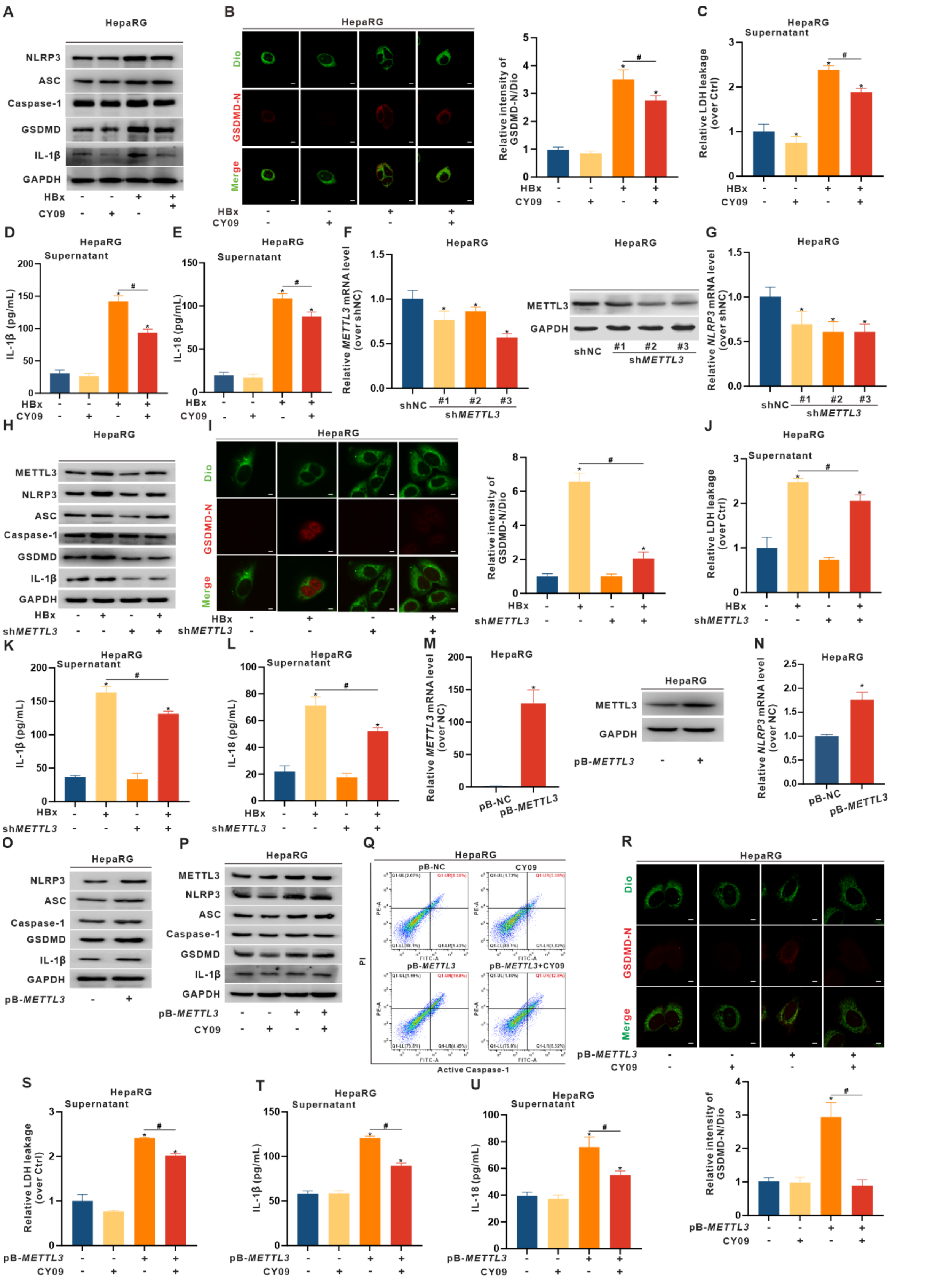

Fig. S3. METTL3 targets *NLRP3* mRNA to mediate pyroptosis in HBx-expressing HepaRG cells. (A-E) HepaRG cells were transfected with pcDNA3.1-HBx (1 μg/mL, 24 h) to construct the HBx-expressing

hepatocytes, followed by treatment with or without CY-09 (10 μM, 24 h), while CY-09 was used as a specific inhibitor of NLRP3 activity. (A) Expression levels of NLRP3, ASC, Caspase-1, GSDMD, and IL-1β proteins were detected by WB. (B) Representative IF images showing co-localization of GSDMD-N (red) and Dio (green) were captured by confocal microscopy (Left). Scale bar, 10 μm. Quantification of the relative intensity of GSDMD-N/Dio is shown in a bar graph (Right). (C-E) Levels of LDH (C), IL-1β (D), and IL-18 (E) in supernatants. (F-L) HBx-expressing HepaRG cells were transfected with sh*METTL3* (1 μg/mL, 24 h) to knock down METTL3, while shNC was transfected as a negative control. (F) Levels of *METTL3* mRNA and METTL3 protein in cells knocked down by serial sh*METTL3* (#1-#3). (G) The level of *NLRP3* mRNA was detected. (H) Expression levels of METTL3, NLRP3, and pyroptosis-related proteins. (I) Representative IF images showing co-localization of GSDMD-N (red) and Dio (green) in cells (Left), while the quantification is shown in a bar graph (Right). Scale bar, 10 μm. (J-L) Levels of LDH (J), IL-1β (K), and IL-18 (L) in supernatants. (M-O) A pB-*METTL3* recombinant plasmid was constructed and transfected to construct the stable METTL3 overexpression (OE) in HepaRG cells, while pB-NC was used as a control. (M) Levels of *METTL3* mRNA and METTL3 protein in cells. (N) The level of *NLRP3* mRNA was detected. (O) Expression levels of NLRP3 and pyroptosis-related proteins in cells. (P-U) METTL3-overexpressing HepaRG cells were pre-treated with or without CY-09 (10 μM, 24 h) to inhibit NLRP3 activity. (P) Expression levels of METTL3, NLRP3, and pyroptosis-related proteins. (Q) Representative images showing the flow cytometry quantification of caspase-1 activity related to pyroptosis. (R) Representative IF images showing co-localization of GSDMD-N (red) and Dio (green) (Upper), while the quantification is shown in a bar graph (Lower). Scale bar, 10 μm. (S-U) Levels of LDH (S), IL-1β (T), and IL-18 (U) in supernatants. Data are presented as mean ± SD. *, $P < 0.05$, compared to the control group. #, $P < 0.05$, compared to the corresponding group.

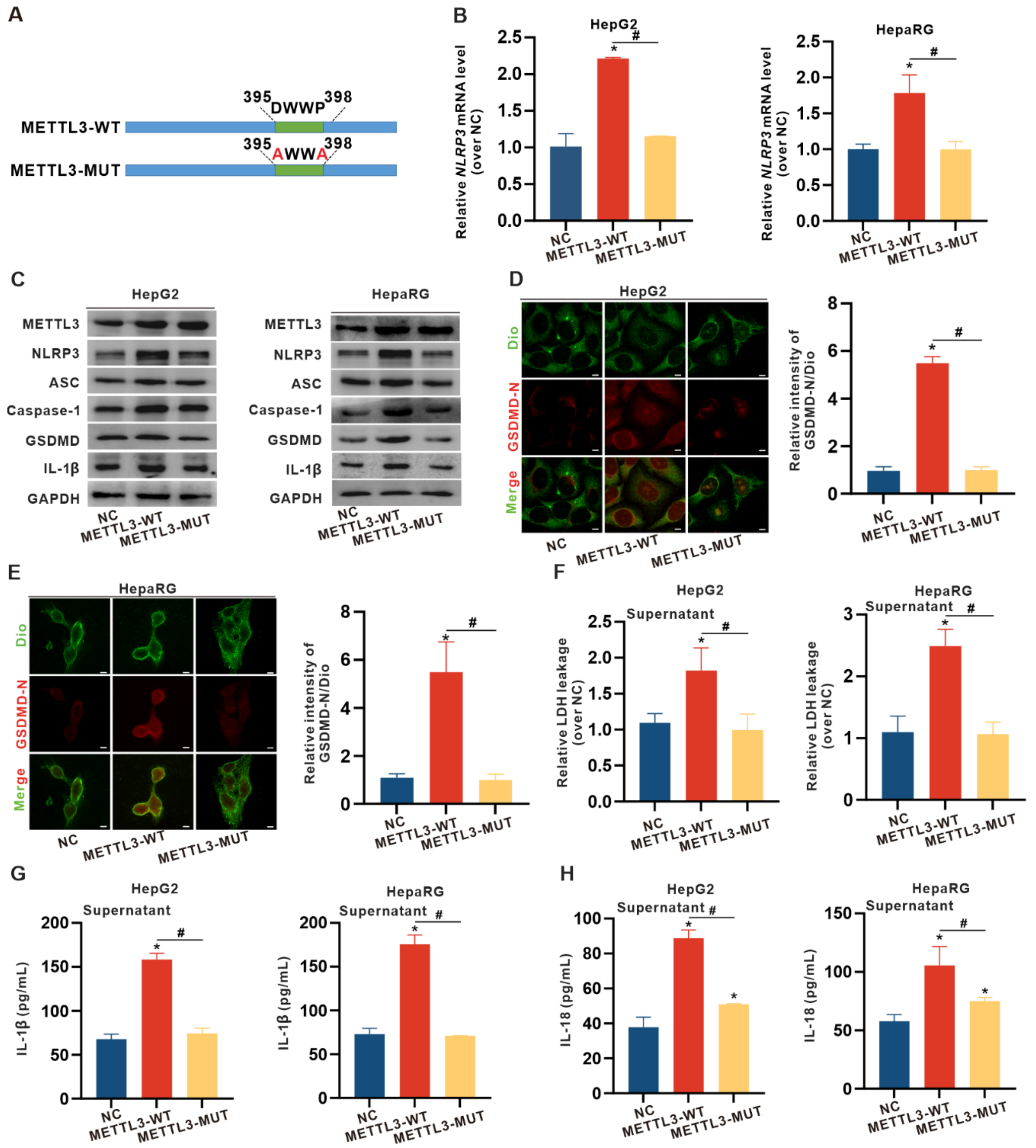

Fig. S4. Catalytic activity of METTL3 regulates *NLRP3* mRNA expression to mediate pyroptosis in HBx-expressing cells. (A-H) METTL3-overexpressing HepG2 and HepaRG cells with wild-type (METTL3-WT) or a catalytic mutant (DWWP to AWWA) (METTL3-MUT) were constructed using recombinant plasmids. (A) Schematic representation of METTL3-WT and -MUT constructs. The conserved methyltransferase motif residues 395–398 (DWWP) were replaced by alanines (AWWA) to abolish enzymatic activity. (B) The level of *NLRP3* mRNA was detected. (C) Expression levels of METTL3, NLRP3, and pyroptosis-related proteins were measured by WB. (D-E) Representative IF images showing co-localization of GSDMD-N (red) and Dio (green) in the HepG2 (D) and HepaRG (E) cells (Left), while the

quantification of the relative intensity of GSDMD-N/Dio is shown in a bar graph (Right). Scale bar, 10 μm. (F-H) Levels of LDH (F), IL-1β (G), and IL-18 (H) in supernatants. Data are presented as mean ± SD. *, $P < 0.05$, compared to the control group. #, $P < 0.05$, compared to the corresponding group.

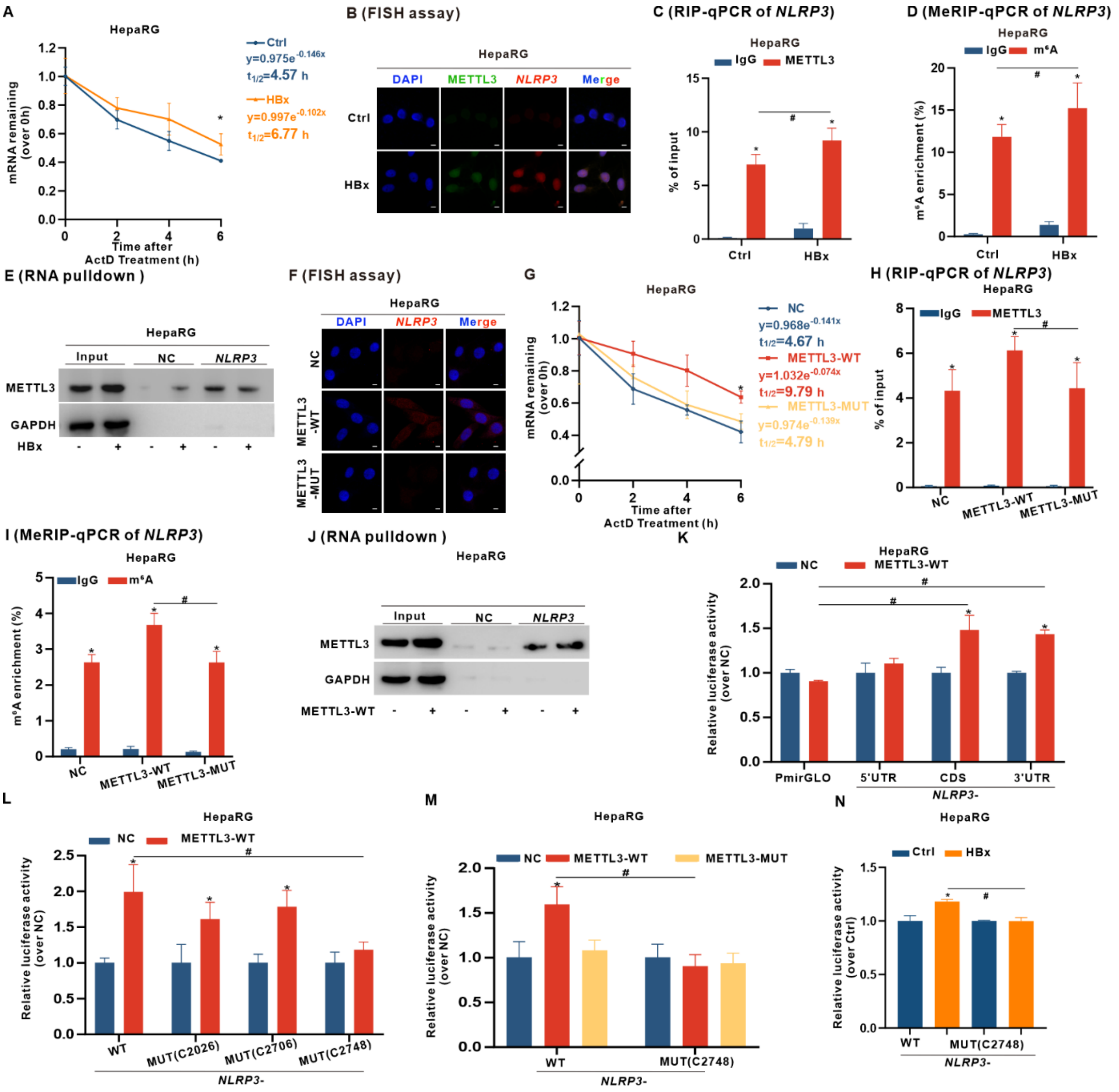

Fig. S5. METTL3-targeting function regulates *NLRP3* mRNA stability in an A2748 site m6A-dependent manner in HBx-expressing HepRG cells. (A-E) HepaRG cells were transfected with pcDNA3.1-HBx (1 µg/ml, 24 h) to construct HBx-expressing hepatocytes. (A) The mRNA stability assay for half-life of *NLRP3* mRNA transcript was measured by qRT-PCR in cells treated with actinomycin D (ActD, 5 mg/ml) at the indicated time points. (B) Representative FISH images showing the staining of METTL3 (green) and *NLRP3* mRNA (red) in cells, while DAPI (blue) was used to counterstain the nuclei. Scale bar, 20 µm. (C) RIP-qPCR analysis of *NLRP3* mRNA illustrating the binding interaction between *NLRP3* mRNA and METTL3. (D) MeRIP-qPCR analysis of m6A levels on *NLRP3* mRNA. (E) RNA pulldown assay using an *NLRP3* mRNA probe followed by detection of METTL3 protein by WB. (F-I) Stable METTL3-overexpressing HepaRG cells were generated using recombinant plasmids encoding wild-type (METTL3-

WT) or a catalytically inactive mutant (DWWP to AWWA) (METTL3-MUT). (F) Representative FISH images showing the staining of *NLRP3* mRNA (red) and DAPI (blue) in cells. Scale bar, 20 μm. (G) Half-life of *NLRP3* mRNA transcript was measured by the mRNA stability assay. (H) RIP-qPCR analysis of *NLRP3* mRNA illustrating the binding interaction between *NLRP3* mRNA and METTL3. (I) MeRIP-qPCR analysis of m6A levels on *NLRP3* mRNA. (J) RNA pulldown assay using an *NLRP3* mRNA probe followed by detection of METTL3 protein by WB. (K) Relative luciferase activities of *NLRP3*-5'UTR, -CDS, and -3'UTR luciferase reporters were detected in NC or METTL3-WT-overexpressing HepaRG cells. (L-N) pmirGLO-*NLRP3*-WT, -MUT(C2026), -MUT(C2706), and -MUT(C2748) luciferase reporter gene recombinant plasmids were constructed by inserting WT or site-directed mutants (MUT) of *NLRP3* CDS fragments, followed by transfection into cells. (L) Relative luciferase activities of *NLRP3*-WT and -MUT luciferase reporters in NC or METT3-WT-overexpressing HepaRG cells. (M) Relative luciferase activities of *NLRP3*-WT or -MUT(C2748) luciferase reporters in NC, METTL3-WT-, and METTL3-MUT-overexpressing HepaRG cells. (N) Relative luciferase activities of *NLRP3*-WT or -MUT(C2748) luciferase reporters in HBx-expressing HepaRG cells. Data are presented as mean ± SD. *, $P < 0.05$, compared to the control group. #, $P < 0.05$, compared to the corresponding group.

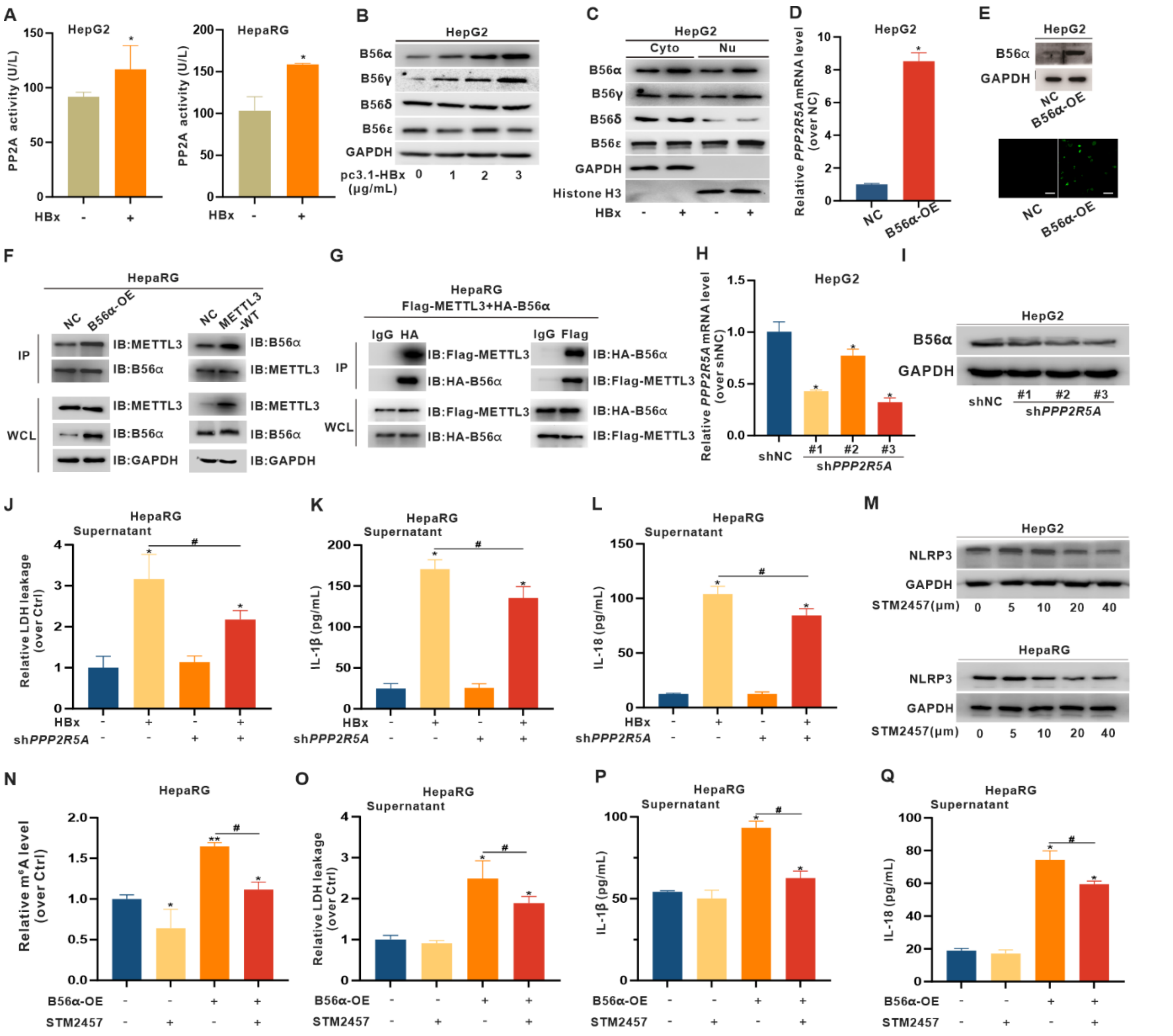

Fig. S6. HBx-induced B56α-interacting METTL3 increases *NLRP3* mRNA m6A levels to mediate pyroptosis in HepaRG cells. (A-C) HepG2 and HepaRG cells were transfected with pcDNA3.1-HBx (1 μg/ml, 24 h) to construct HBx-expressing hepatocytes, while the negative control (NC) cells transfected with pcDNA3.1 vector served as controls. (A) PP2A enzyme activity was detected by ELISA. (B) Expression levels of B56α, B56γ, B56δ, and B56ε proteins were detected by WB. (C) Levels of B56α, B56γ, B56δ, and B56ε proteins in cytoplasmic and nuclear fractions. (D-E) A pB-B56α recombinant plasmid was constructed and transfected to construct the stable B56α overexpression (OE) in HepG2 cells, while pB-NC served as controls. Levels of *METTL3* mRNA (D) and METTL3 protein (E) in cells. (F) HepaRG cells overexpressing B56α (B56α-OE) or METTL3-WT were constructed. Co-IP analysis showing the binding interaction between endogenous METTL3 and B56α proteins. (G) HepaRG cells were co-transfected with Flag-METTL3 and HA-B56α recombinant plasmids for 24 h, followed by subjecting the cell extracts to Co-IP analysis. The

binding interaction between exogenous Flag-METTL3 and HA-B56α fusion proteins was detected by WB with anti-HA and anti-Flag antibodies. (H-I) Levels of *PPP2R5A* mRNA and B56α protein in cells knocked down by serial sh*PPP2R5A* (#1-#3). (J-L) HBx-expressing HepaRG cells were transfected with sh*PPP2R5A* (1 μg/mL, 24 h) to knock down B56α expression, while shNC was used as a negative control. The release levels of LDH (J), IL-1β (K), and IL-18 (L) in supernatants were detected. (M) Levels of NLRP3 were detected by WB in cells treated with different concentrations of STM2457. (N-Q) B56α-OE HepaRG cells were treated with STM2457 (20 μM, 24h) to inhibit m6A catalytic activity of METTL3. (N) Global RNA m6A levels in cells was measured. (O-Q) The release levels of LDH (O), IL-1β (P), and IL-18 (Q) in supernatants were detected. Data are presented as mean ± SD. *, $P < 0.05$, compared to the control group. #, $P < 0.05$, compared to the corresponding group.

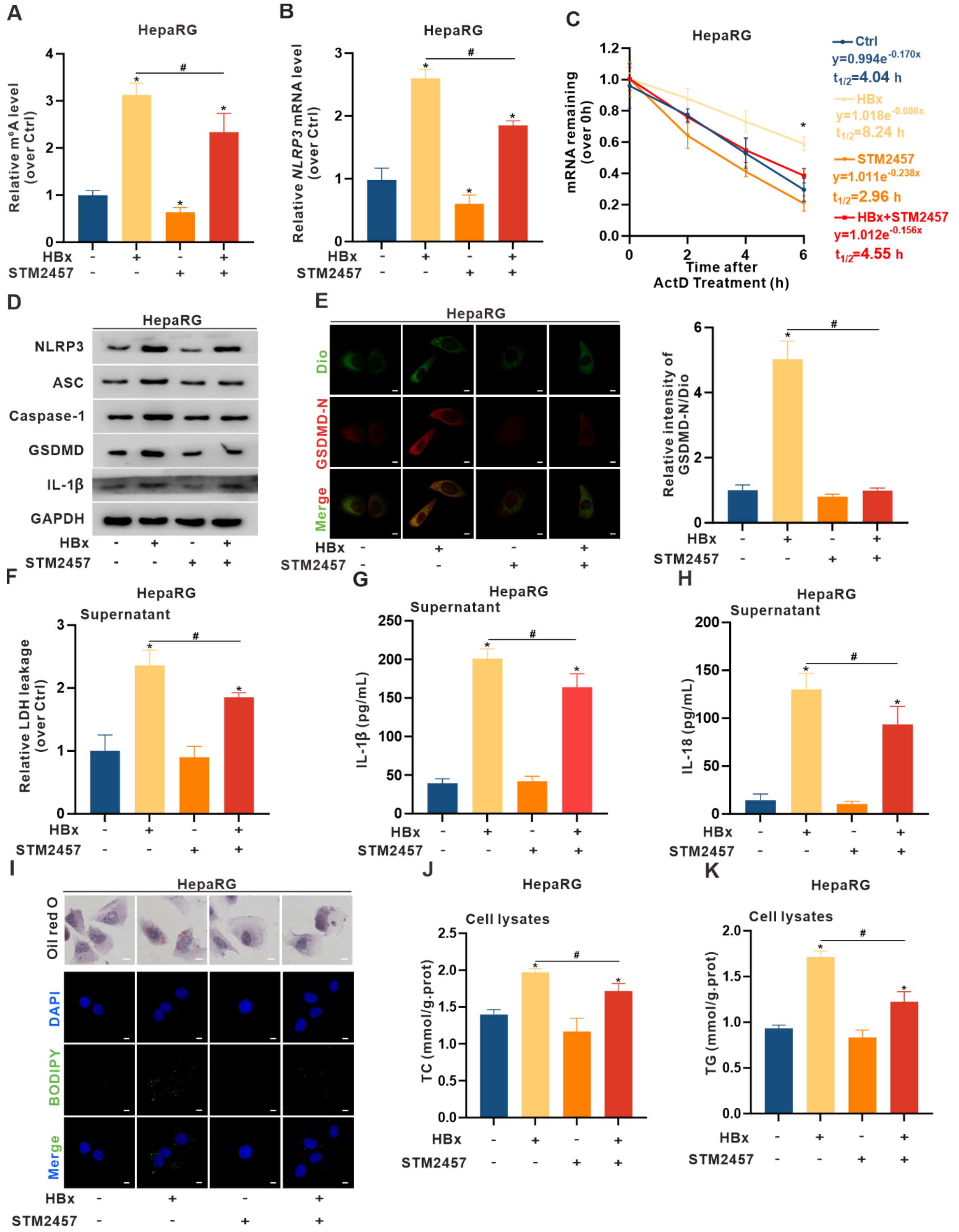

Fig. S7. HBx-induced pyroptosis and lipotoxicity are suppressed by STM2457-mediated inhibition of METTL3 in HBx-expressing HepaRG cells. HBx-expressing HepaRG cells were treated with STM2457 (20 µM, 24 h). (A) Global RNA m6A levels in cells were measured. (B) *NLRP3* mRNA levels were detected by qRT-PCR. (C) The mRNA stability assay for half-life of *NLRP3* mRNA transcript was measured by qRT-PCR in cells treated with actinomycin D (ActD, 5 µg/ml) at the indicated time points. (D) Expression levels

of NLRP3, ASC, Caspase-1, GSDMD, and IL-1β proteins were detected by WB. (E) Representative IF images showing co-localization of GSDMD-N (red) and Dio (green) were captured by confocal microscopy (Left). Scale bar, 10 μm. Quantification of the relative intensity of GSDMD-N/Dio is shown in a bar graph (Right). (F-H) The release levels of LDH (F), IL-1β (G), and IL-18 (H) in supernatants were detected. (I) Representative images showing Oil red O staining (Scale bar, 100 μm) and IF staining of LD (Scale bar, 10 μm) in cells. (J-K) The levels of TC (J) and TG (K) in cells were detected. Data are presented as mean ± SD, n = 3. *, $P < 0.05$, compared to the control group. #, $P < 0.05$, compared to the corresponding group.

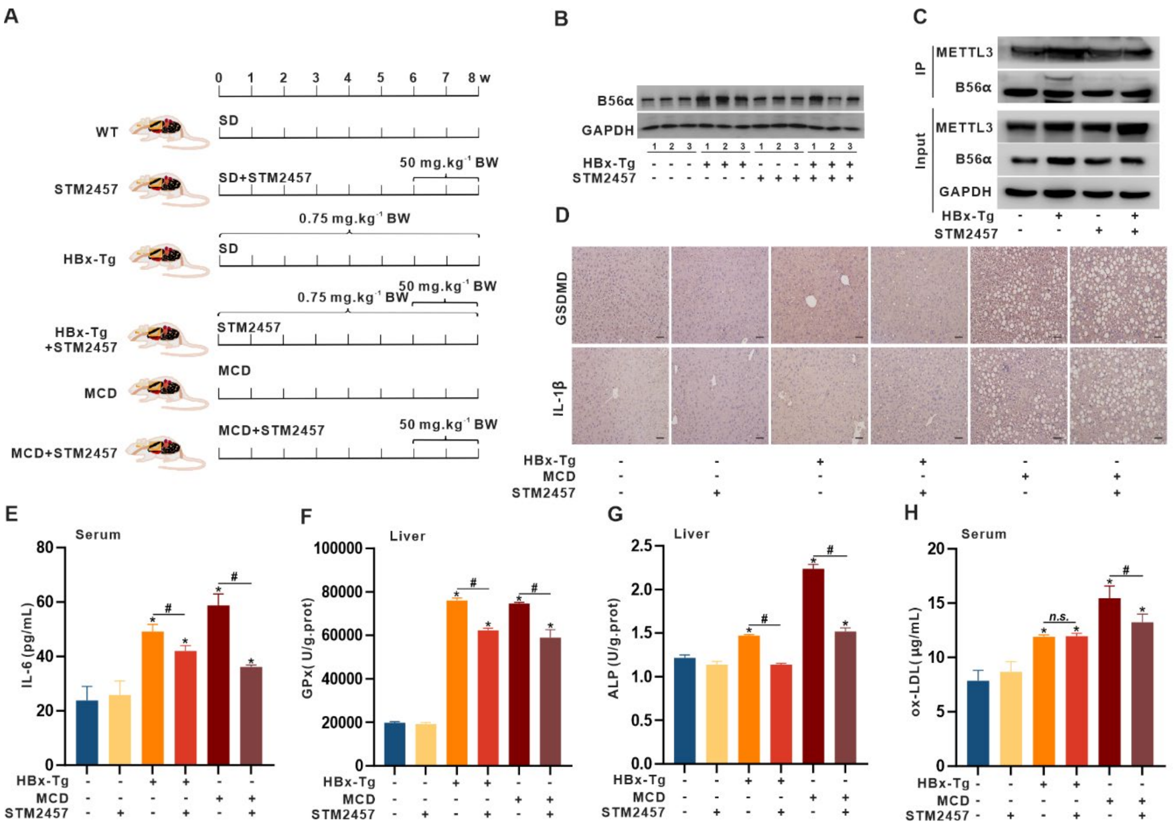

Fig. S8. METTL3 intervention by STM2457 alleviates NLRP3-dependent MASH in HBx-Tg mice *in vivo*. HBx-Tg mice were used to construct the MASH model, and MCD-fed mice served as the positive model. Mice revived daily intraperitoneal injection of STM2457 (50 mg•kg^{-1} BW) for two weeks, while wild-type mice served as the negative control. n = 5 per group. (A) Schematic of the experimental design for STM2457 intervention *in vivo.* (B) Expression levels of B56α protein in livers were detected by WB. (C) Co-IP analysis showed the binding interaction between METTL3 and B56α proteins in livers. (D) Representative IHC staining images showing GSDMD and IL-1β protein expression in liver tissues. Scale bar, 100 μm. (E-H) Levels of IL-6 (E), GPx (F), ALP (G), and ox-LDL (H) were detected. Data are presented as mean ± SD. *, $P < 0.05$, compared to the control group. #, $P < 0.05$, compared to the corresponding group.

Supplementary Tables

Table S1. Information on the primary antibodies used in the present study.

Antibody	Source	Catalog No.	Application (Dilution)
HBx(anti-mouse)	Santa Cruz	sc-57760	WB(1:1000)
NLRP3(anti-mouse)	Proteintech	68102-1-Ig	WB(1:2000); IHC(1:200)
ASC(anti-rabbit)	Proteintech	10500-1-AP	WB(1:500)
Caspase1(anti-mouse)	Santa Cruz	sc-392736	WB(1:500)
GSDMD(anti-mouse)	Proteintech	66387-1-Ig	WB(1:1000); IF(1:200); IHC(1:200)
IL-1β(anti-mouse)	Proteintech	66737-1-Ig	WB(1:1000); IHC(1:200)
METTL3(anti-rabbit)	Abcam	ab195352	WB(1:1000); IF(1:200); IP(1:50); IHC(1:500)
B56α(anti-rabbit)	Proteintech	12675-2-AP	WB(1:1000)
B56γ(anti-rabbit)	ABclonal	A5480	WB(1:1000)
B56δ(anti-rabbit)	ABclonal	A15707	WB(1:1000)
B56ε(anti-rabbit)	ABclonal	A15084	WB(1:1000)
METTL14(anti-rabbit)	Proteintech	26158-1-AP	WB(1:2000); IHC(1:500)
WTAP(anti-rabbit)	Abcam	ab195380	WB(1:1000)
Histone H3(anti-mouse)	Proteintech	68345-1-Ig	WB(1:5000)
HA tag(anti-rabbit)	Proteintech	51064-2-AP	WB(1:5000)); IP(0.5-4.0 ug)
Flag tag(anti-mouse)	Proteintech	66008-4-Ig	WB(1:5000); IP(0.5-4.0 ug)

Table S2. Sequences of primers used for various plasmids construction.

Plasmids	Primers	Sequences
PB513B-1-*METTL3*	FP	5'-CTAGCTAGCGCCACCATGTCGGACACGTGGAGCTCTATC-3'
	RP	5'-ATAAGCATGCGGCCGCCTATAAATTCTTAGGTTTAGAGATGATAC-3'
PB513B-1-*PPP2R5A*-HA	FP	5'-CGCGGATCCATGTACCCATACGATGTTCCAGATTACGCTGCCACCATGTCGTCGTCGTC-3'
	RP	5'-ATAAGAATGCGGCCGCTTATTCGGCACTTGTATTGC-3'
METTL3 (D395A-P398A)	FP	5'-TGTGATGGCTGCCCCACCCGCGGATATTCACATGGAACTGCCCTATGGG-3'
	RP	5'-ATGTGAATATCCGCGGGTGGGGCAGCCATCACAACTGCAAACTTGCCC-3'
METTL3(1-182)	RP	5'-ATAAGAATGCGGCCGCCTACTGTTCTGCACGCCGCTTCTGC-3'
METTL3(1-215)	RP	5'-ATAAGAATGCGGCCGCCTATTTCCTTGATTTCTTGGCTGGCTCC-3'
METTL3(1-298)	RP	5'-ATAAGAATGCGGCCGCCTAGTGCAGCTTGCGACA-3'
METTL3(1-336)	RP	5'-ATAAGAATGCGGCCGCCTAGCAAGCATCAATTTCATAGTG-3'
METTL3 (336-580)	FP	5'-CTAGCTAGCATGGATTACAAGGATGACGACGATAAGATGGATTCTGAGGCCCCTGGC-3'
METTL3-△ZnF	FP	5'-ATGGATTCTGAGGCCCCTGGCAGCAAAG-3'
METTL3-△ZnF	RP	5'-TTGTTCCTTGGCTGTTGTAGTATTTAATAGCTCTAG-3'
METTL3-shRNA-1	FP	5'-CCGGGCCTTAACATTGCCCACTGATCTCGAGATCAGTGGGCAATGTTAAGGCTTTTTG-3'
	RP	5'-AATTCAAAAAGCCTTAACATTGCCCACTGATCTCGAGATCAGTGGGCAATGTTAAGGC-3'
METTL3-shRNA-2	FP	5'-CCGGGCCAAGGAACAATCCATTGTTCTCGAGAACAATGGATTGTTCCTTGGCTTTTTG-3'
	RP	5'-AATTCAAAAAGCCAAGGAACAATCCATTGTTCTCGAGAACAATGGATTGTTCCTTGGC-3'
METTL3-shRNA-3	FP	5'-CCGGCGTCAGTATCTTGGGCAAGTTCTCGAGAACTTGCCCAAGATACTGACGTTTTTG-3'
	RP	5'-AATTCAAAAACGTCAGTATCTTGGGCAAGTTCTCGAGAACTTGCCCAAGATACTGACG-3'
PPP2R5A-shRNA-1	FP	5'-CCGGCACTGAATGAACTGGTTGAGTCTCGAGACTCAACCAGTTCATTCAGTGTTTTTG-3'
	RP	5'-AATTCAAAAACACTGAATGAACTGGTTGAGTCTCGAGACTCAACCAGTTCATTCAGTG-3'
PPP2R5A-shRNA-2	FP	5'-CCGGCGACCATTGTAGCACTGGTATCTCGAGATACCAGTGCTACAATGGTCGTTTTTG-3'
	RP	5'-AATTCAAAAACGACCATTGTAGCACTGGTATCTCGAGA

		TACCAGTGCTACAATGGTCG-3'
PPP2R5A-shRNA-3	FP	5'-CCGGGCTAACATCTTCCGTACACTTCTCGAGAAGTGTACGGAAGATGTTAGCTTTTTG-3'
	RP	5'-AATTCAAAAAGCTAACATCTTCCGTACACTTCTCGAGAAGTGTACGGAAGATGTTAGC-3'

Table S3. Sequences of primers used for qRT-PCR analysis.

Genes	Primers	Sequences
NLRP3	FP	5'-AGCAGATGGAGAGTGGCAAGAG-3'
	RP	5'-AAGCAGACACATCCGCCTTCT-3'
IL18	FP	5'-TCTTCATTGACCAAGGAAATCGG-3'
	RP	5'-TCCGGGGTGCATTATCTCTAC-3'
IL1B	FP	5'-ATGATGGCTTATTACAGTGGCAA-3'
	RP	5'-GTCGGAGATTCGTAGCTGGA-3'
GSDMD	FP	5'-GTGTGTCAACCTGTCTATCAAGG-3'
	RP	5'-CATGGCATCGTAGAAGTGGAAG-3'
CASP1	FP	5'-TTTCCGCAAGGTTCGATTTTCA-3'
	RP	5'-GGCATCTGCGCTCTACCATC-3'
ASC	FP	5'-TGGATGCTCTGTACGGGAAG-3'
	RP	5'-CCAGGCTGGTGTGAAACTGAA-3'
METTL3	FP	5'-CATTGCCCACTGATGCTGTG-3'
	RP	5'-AGGCTTTCTACCCCATCTTGA-3'
METTL14	FP	5'-GAACACAGAGCTTAAATCCCCA-3'
	RP	5'-TGTCAGCTAAACCTACATCCCTG-3'
WTAP	FP	5'-TTGTAATGCGACTAGCAACCAA-3'
	RP	5'-GCTGGGTCTACCATTGTTGATCT-3'
PPP2R5A	FP	5'-CGTGAGTCCCATTAAGATGGAGT-3'
	RP	5'-CCCGACAGTGGATATAGAACAGA-3'

Table S4. Sequence of the oligonucleotide used for *NLRP3* RNA pulldown.

Target gene	sense
NLRP3	5'-TGCCAGTCAGTGCAGAGCGGTCCTAT-3'

Table S5. Sequences of primers used for constructing the pmirGLO-*NLRP3* reporter.

Reporter genes	Primers	Sequences
pmirGLO-*NLRP3*	FP	5'-CTAGCTAGCTAGATGGCAAGCACCCGCTGCAAG-3'
	RP	5'-GCTCTAGAGCCTACCAAGAAGGCTCAAAGACGAC-3'
pmirGLO-*NLRP3*-2026MUT	FP	5'-GTGTGCACTGGCCTGAAACAGCAGATGGAGAGT-3'
	RP	5'-ATCTGCTGTTTCAGGCCAGTGCACACGATCCAGC-3'
pmirGLO-*NLRP3*-2706MUT	FP	5'-AGGGCCATGGCCTATTTCCCCAAGATTGAGATCAATCTCTCCACC-3'
	RP	5'-TGGGGAAATAGGCCATGGCCCTTTGCACGAAGTCCTCCTCC-3'
pmirGLO-*NLRP3*-2748MUT	FP	5'-TCCACCAGAATGGCCCACATGGTTTCTTCCTTTTG-3'
	RP	5'-GAAACCATGTGGGCCATTCTGGTGGAGAGATTGA-3'

Table S6. Sequence of the probe used for the *NLRP3* FISH assay.

Target gene	Probe sequence
NLRP3	5'-TGCCAGTCAGTGCAGAGCGGTCCTAT-3'
