# Supplementary material for: Hepatic steatosis and pyroptosis are induced by the hepatitis B virus X protein via B56α-METTL3 interaction-mediated m6A modification of the NLRP3 mRNA
Source: Cell Death Dis. 2025 Oct 6;16(1):698. doi: 10.1038/s41419-025-08019-8 (PMC12500908; doi:10.1038/s41419-025-08019-8)
Supplement: Supplementary file 2 — Original Data [file 41419_2025_8019_MOESM2_ESM.pdf]

Image of WB：

Fig.1H:

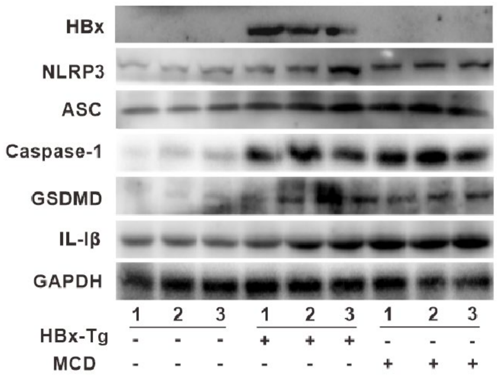

HBx

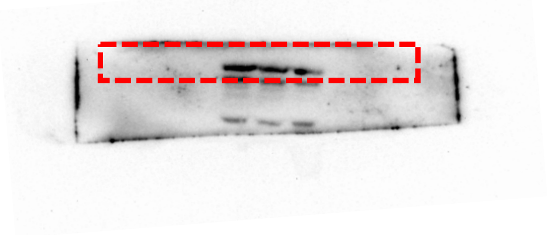

NLRP3

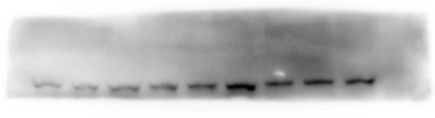

ASC

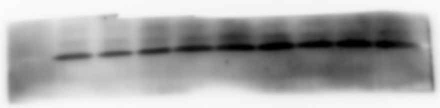

Caspase-1

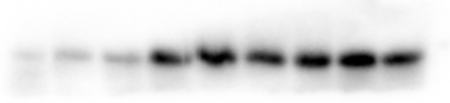

GSDMD

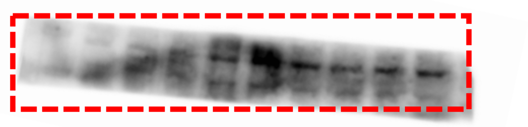

IL-Iβ

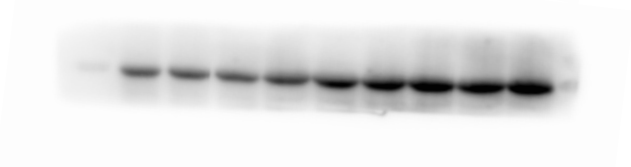

GAPDH

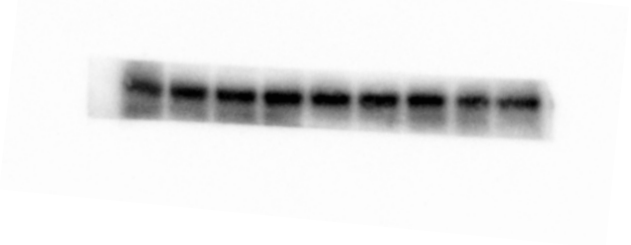

Fig.1P:

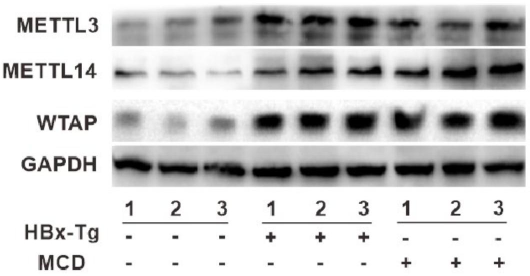

METTL3

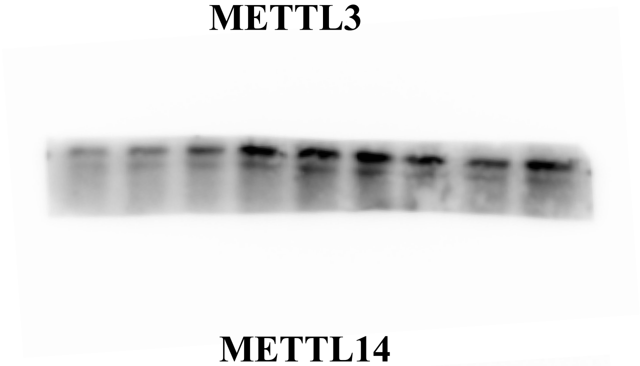

METTL14

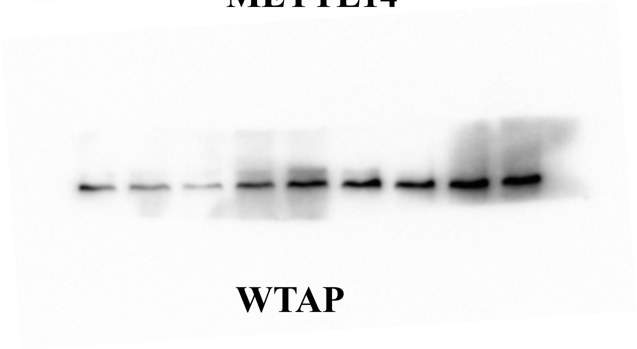

WTAP

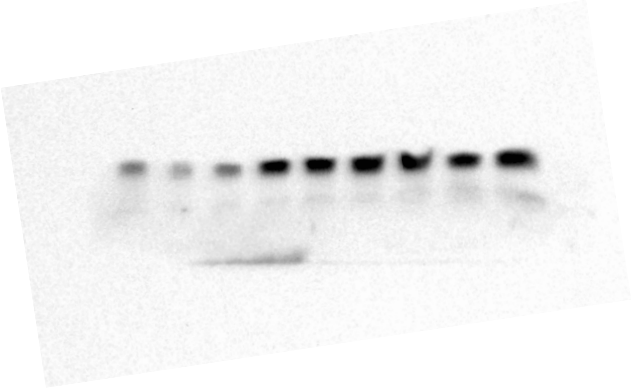

GAPDH

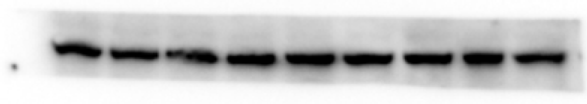

Fig.2D:

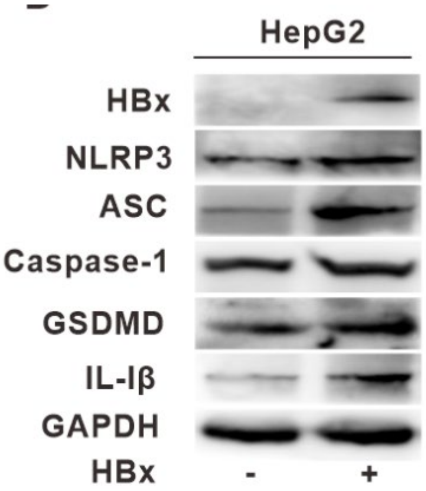
HepG2
HBx
NLRP3
ASC
Caspase-1
GSDMD
IL-Iβ
GAPDH
HBx
-
+

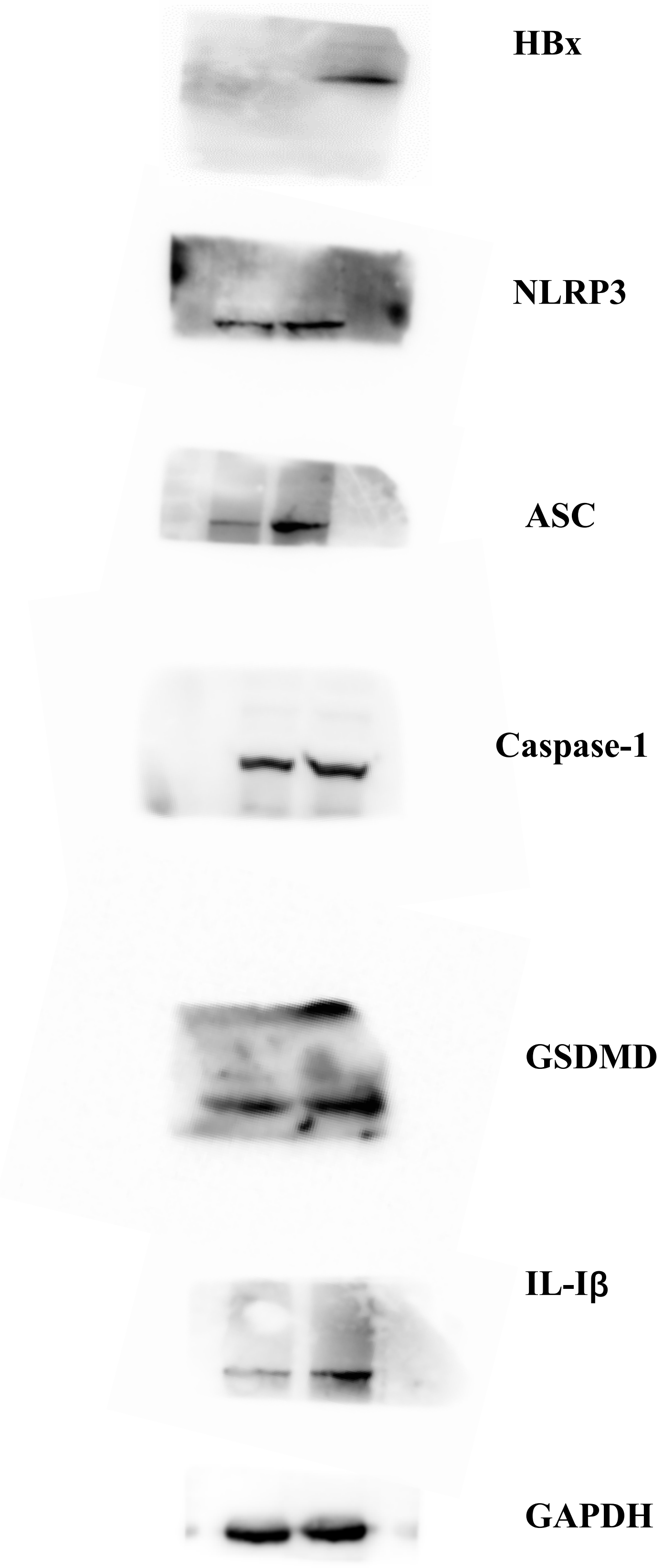
HBx
NLRP3
ASC
Caspase-1
GSDMD
IL-Iβ
GAPDH

Fig.2L:

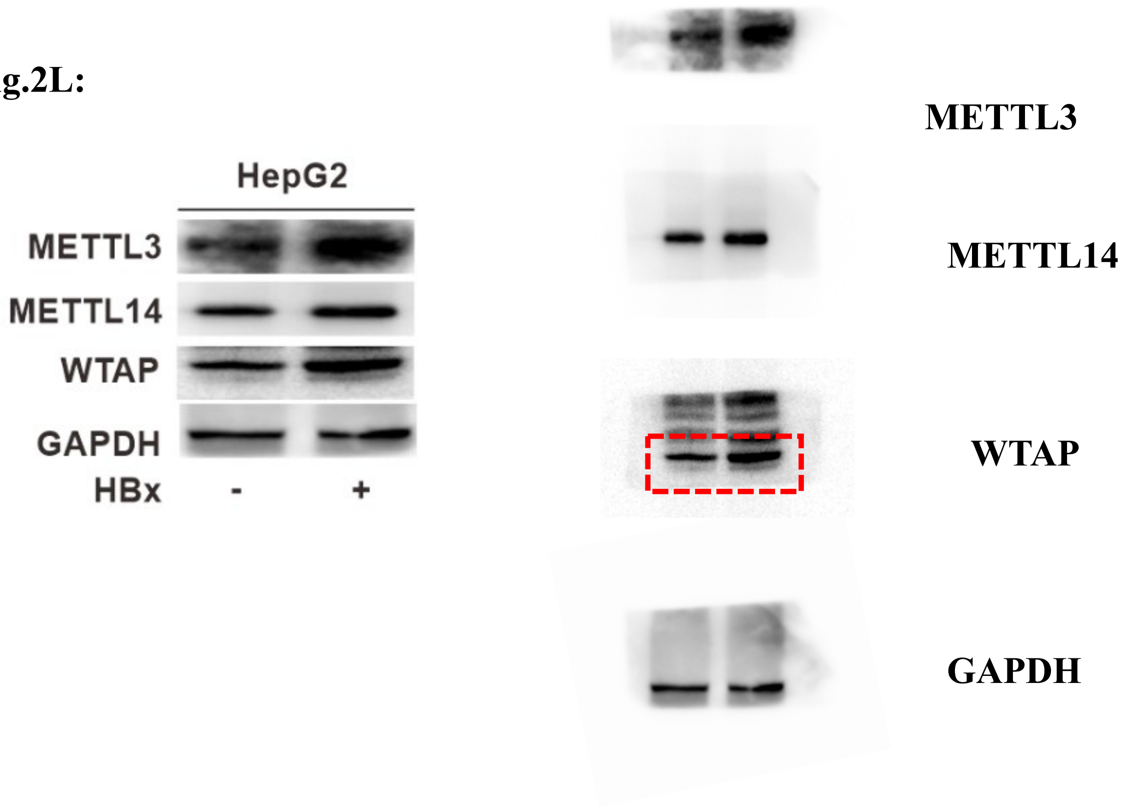
HepG2
METTL3
METTL14
WTAP
GAPDH
HBx - +
METTL3
METTL14
WTAP
GAPDH

Fig.3A:

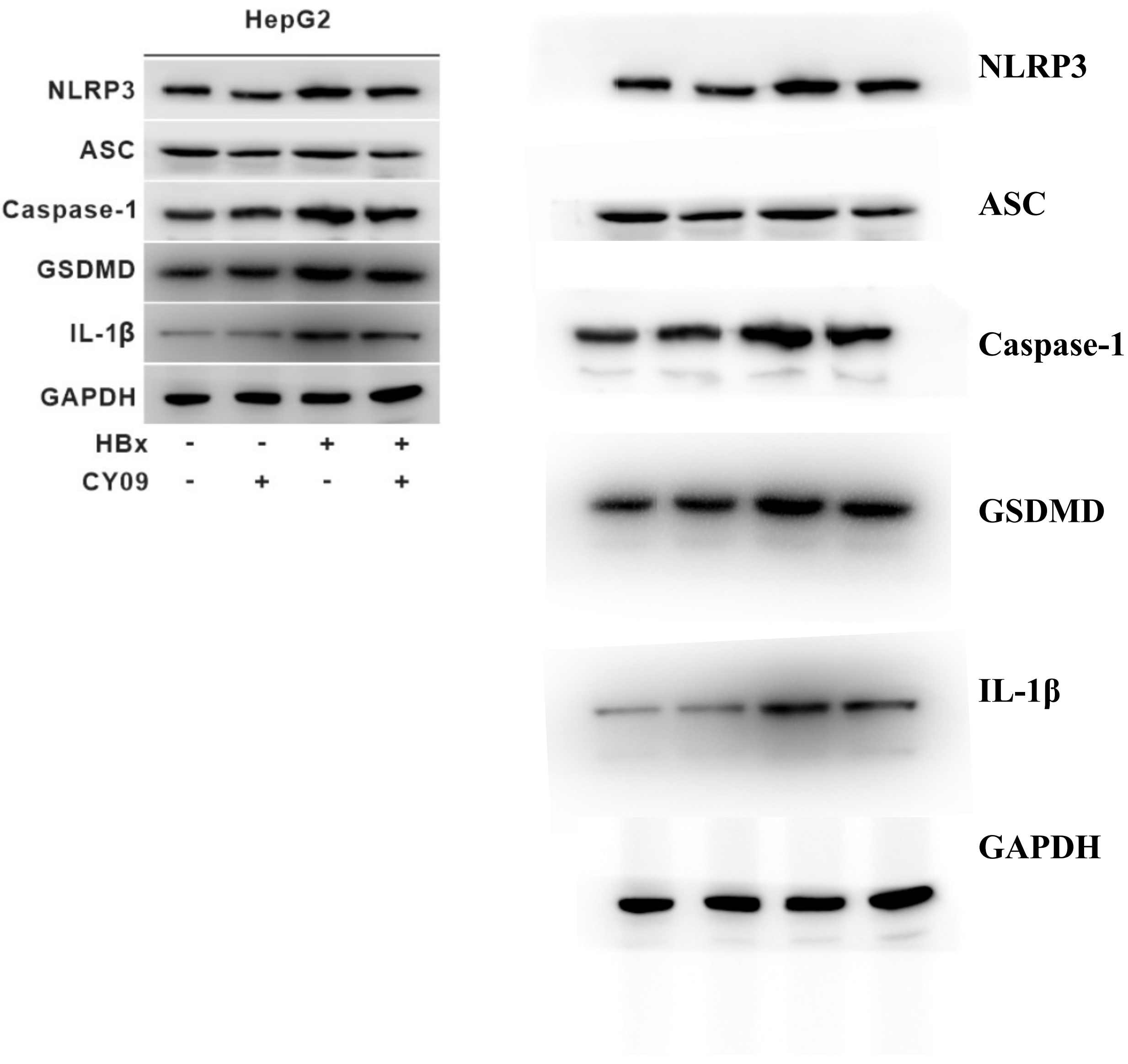
HepG2
NLRP3
ASC
Caspase-1
GSDMD
IL-1β
GAPDH
HBx - - + +
CY09 - + - +
NLRP3
ASC
Caspase-1
GSDMD
IL-1β
GAPDH

Fig.3F

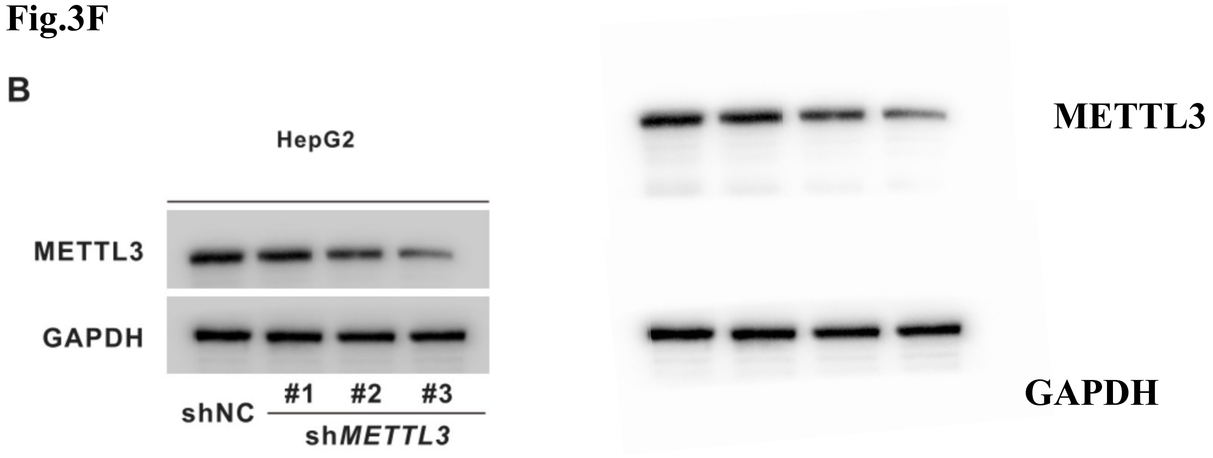
B
HepG2
METTL3
GAPDH
shNC
#1
#2
#3
shMETTL3
METTL3
GAPDH

Fig.3H

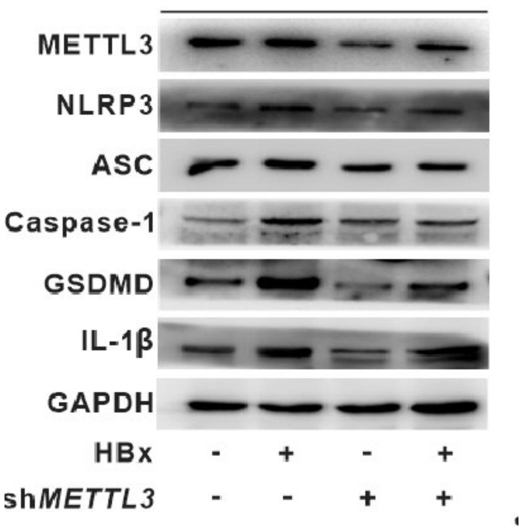
METTL3
NLRP3
ASC
Caspase-1
GSDMD
IL-1β
GAPDH
HBx - + - +
shMETTL3 - - + +

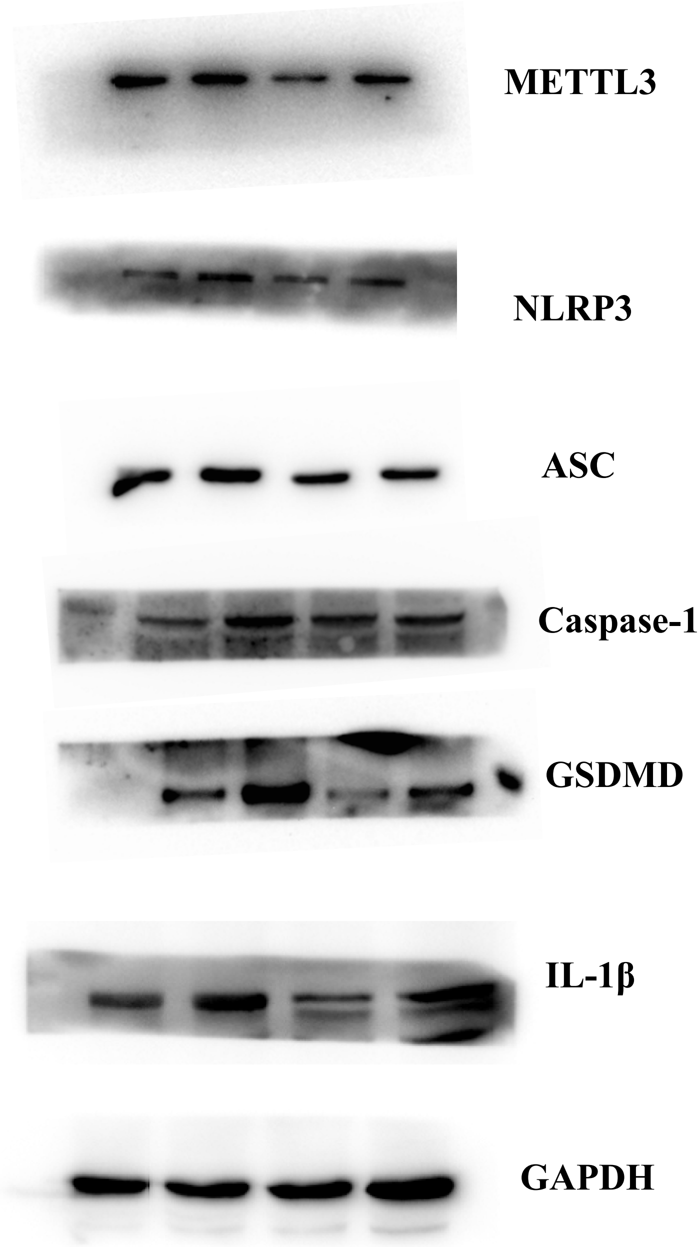
METTL3
NLRP3
ASC
Caspase-1
GSDMD
IL-1β
GAPDH

Fig.3O

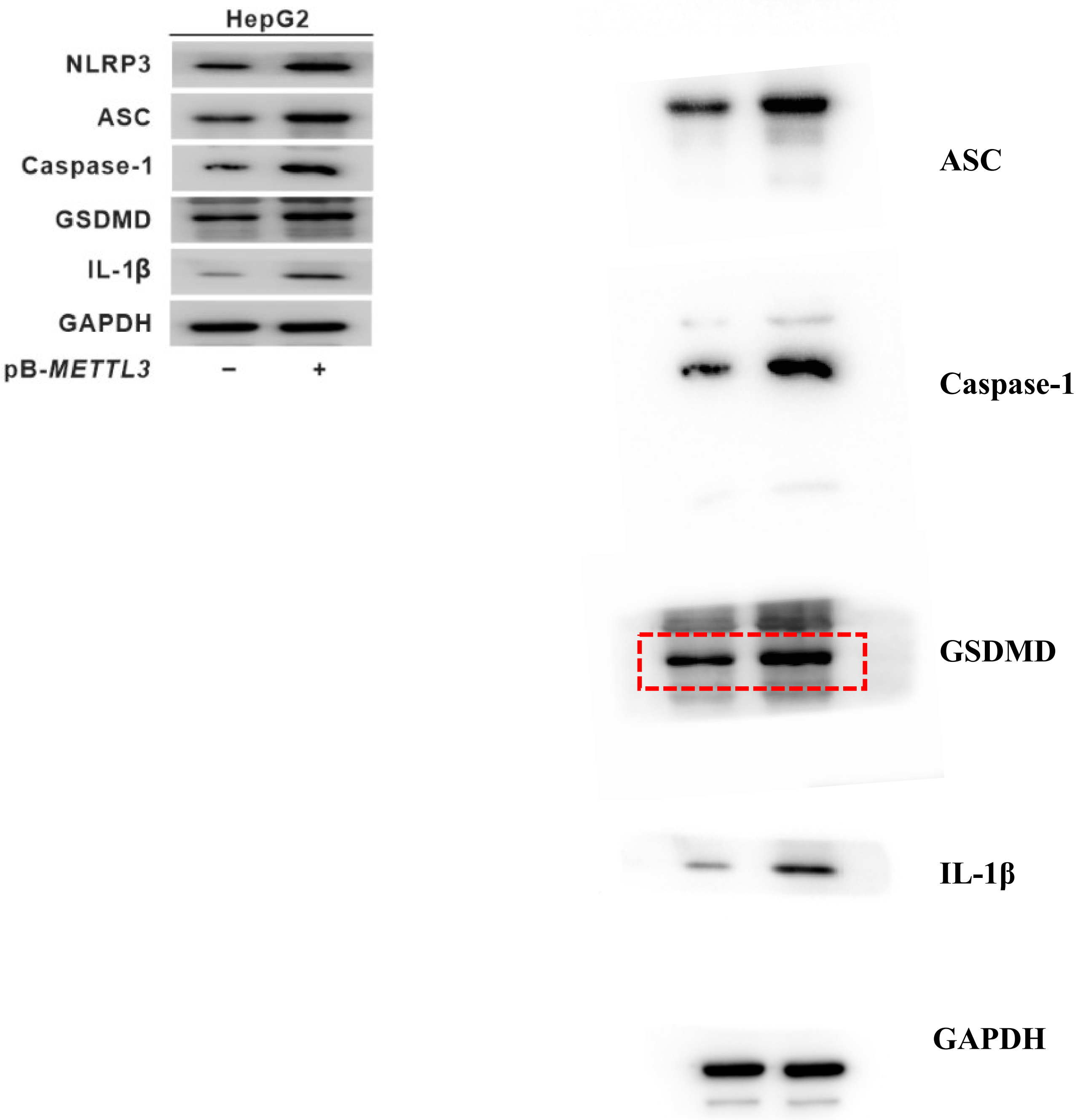

HepG2
NLRP3
ASC
Caspase-1
GSDMD
IL-1β
GAPDH
pB-*METTL3*
−
+
NLRP3
ASC
Caspase-1
GSDMD
IL-1β
GAPDH

Fig.3P

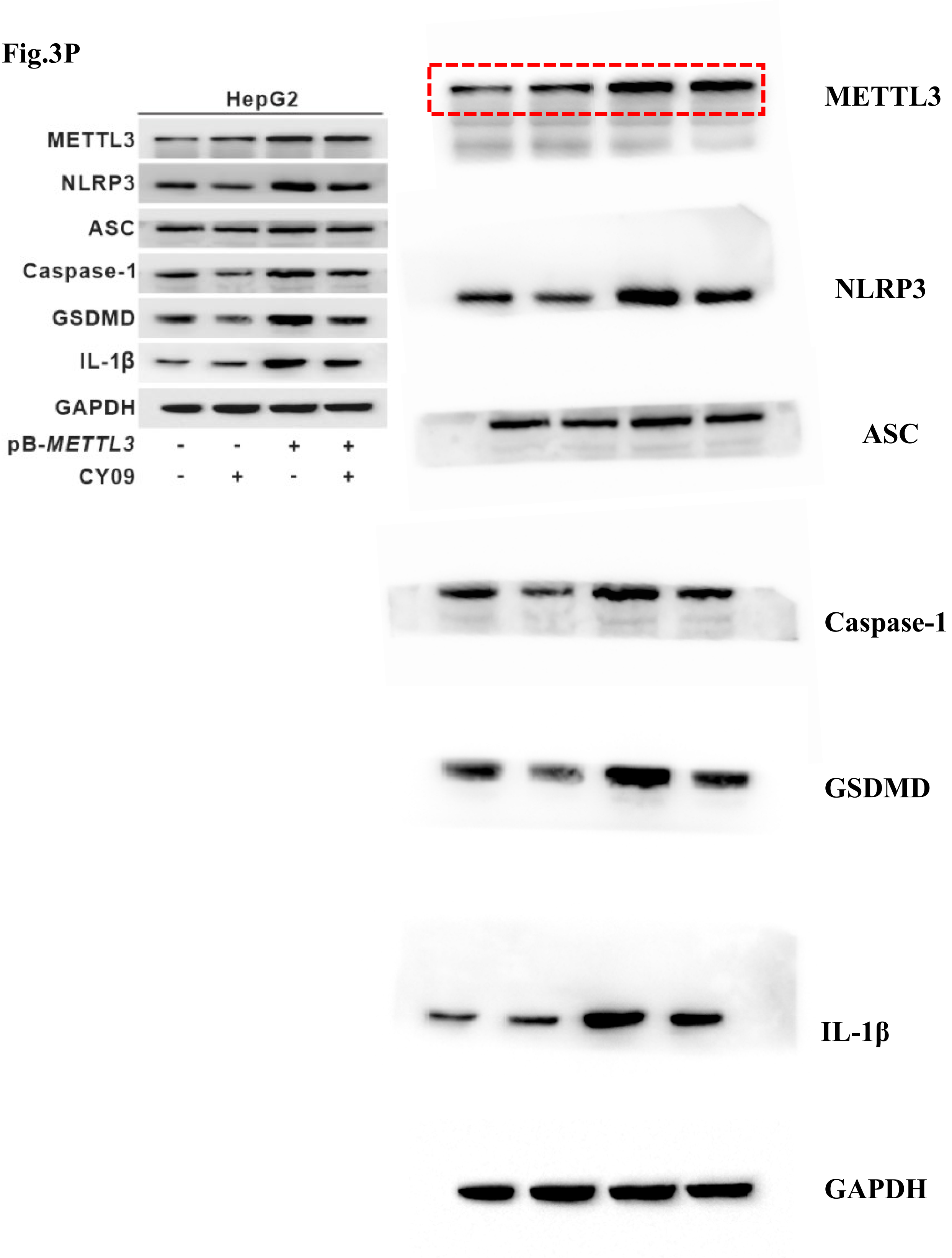
HepG2
METTL3
NLRP3
ASC
Caspase-1
GSDMD
IL-1β
GAPDH
pB-*METTL3* - - + +
CY09 - + - +
METTL3
NLRP3
ASC
Caspase-1
GSDMD
IL-1β
GAPDH

Fig.4E

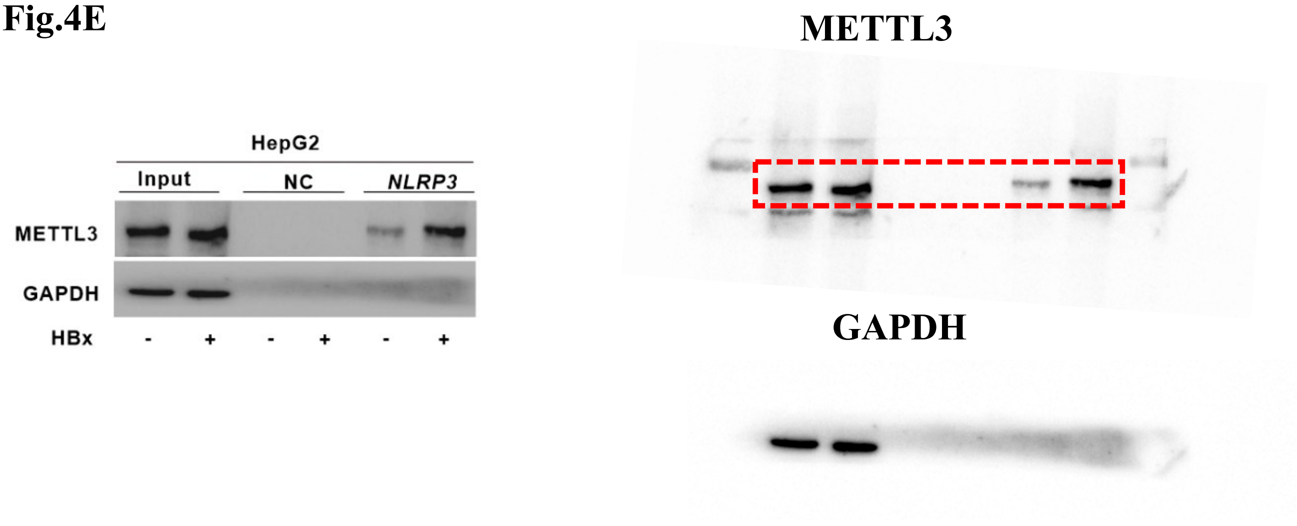
HepG2
Input
NC
NLRP3
METTL3
GAPDH
HBx - + - + - +
METTL3
GAPDH

Fig.4J

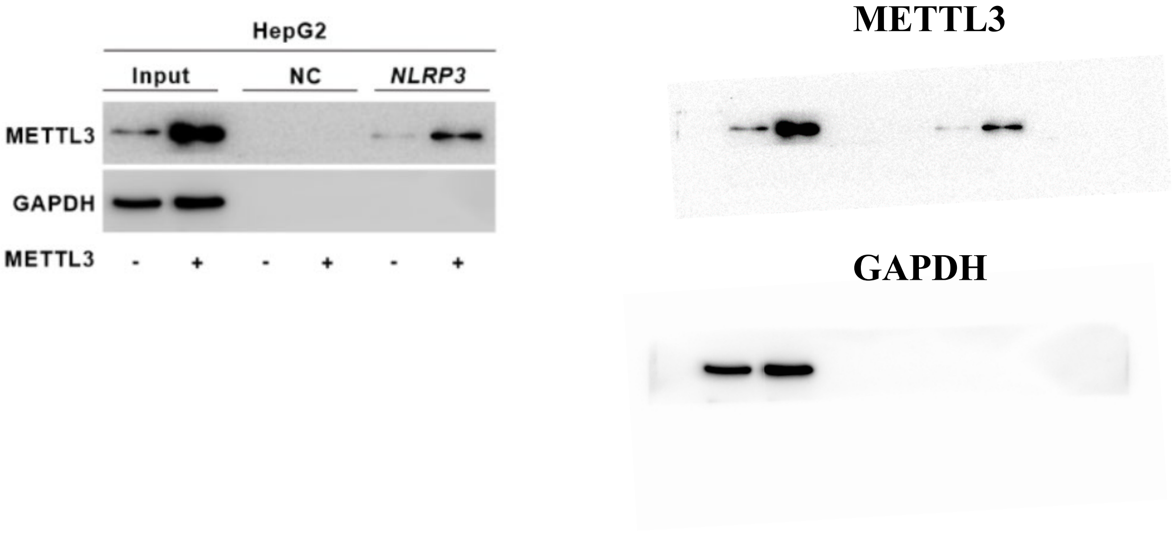
HepG2
Input
NC
NLRP3
METTL3
GAPDH
METTL3 - + - + - +
METTL3
GAPDH

Fig.5E

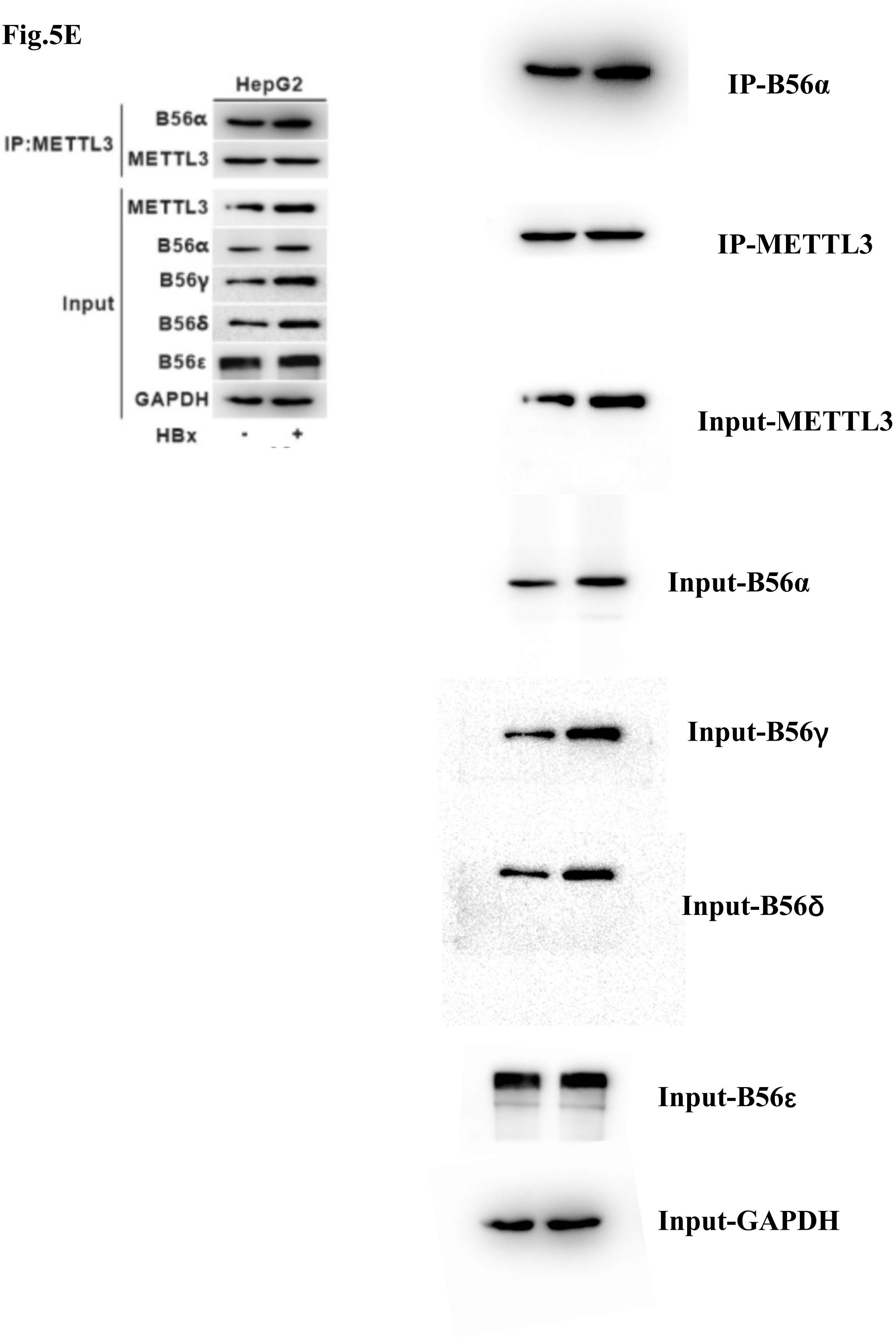
HepG2
IP:METTL3
B56α
METTL3
Input
METTL3
B56α
B56γ
B56δ
B56ε
GAPDH
HBx
-
+
IP-B56α
IP-METTL3
Input-METTL3
Input-B56α
Input-B56γ
Input-B56δ
Input-B56ε
Input-GAPDH

Fig.5F

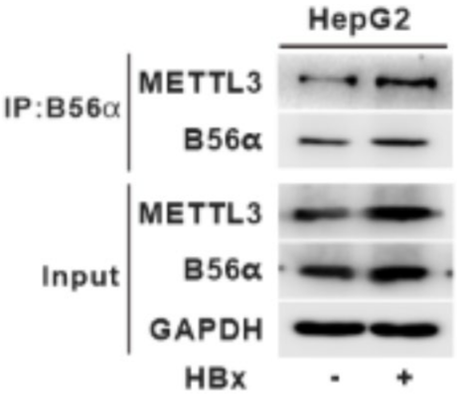

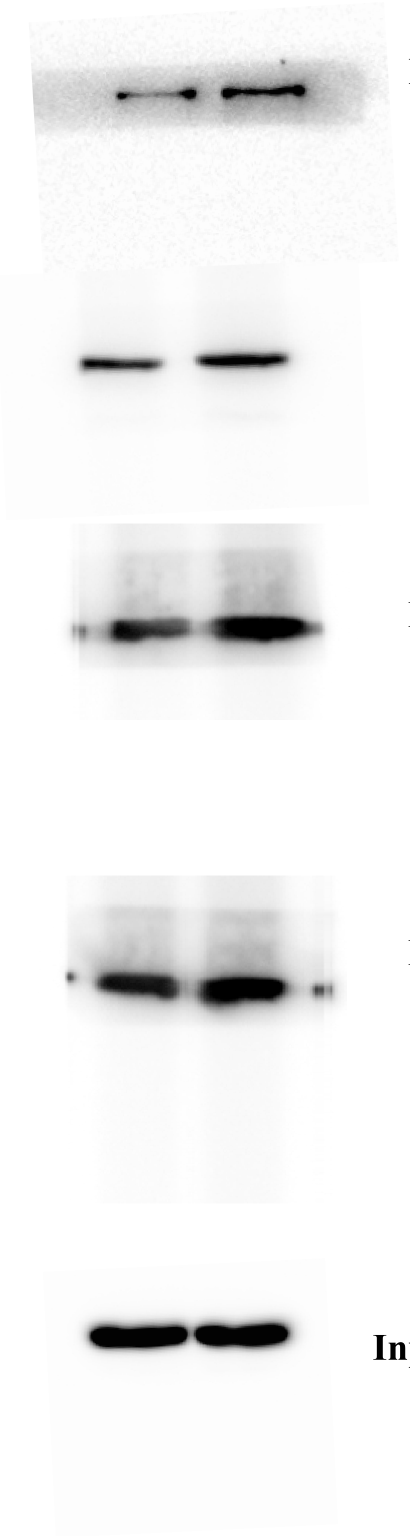

IP-METTL3

IP-B56α

Input-METTL3

Input-B56α

Input-GAPDH

Fig.5H

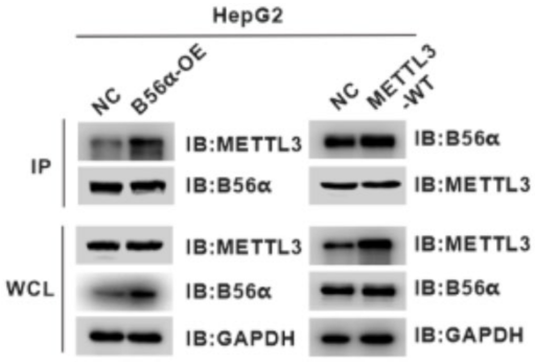
HepG2
NC
B56α-OE
NC
METTL3
-WT
IP
WCL
IB:METTL3
IB:B56α
IB:METTL3
IB:B56α
IB:GAPDH
IB:B56α
IB:METTL3
IB:METTL3
IB:B56α
IB:GAPDH

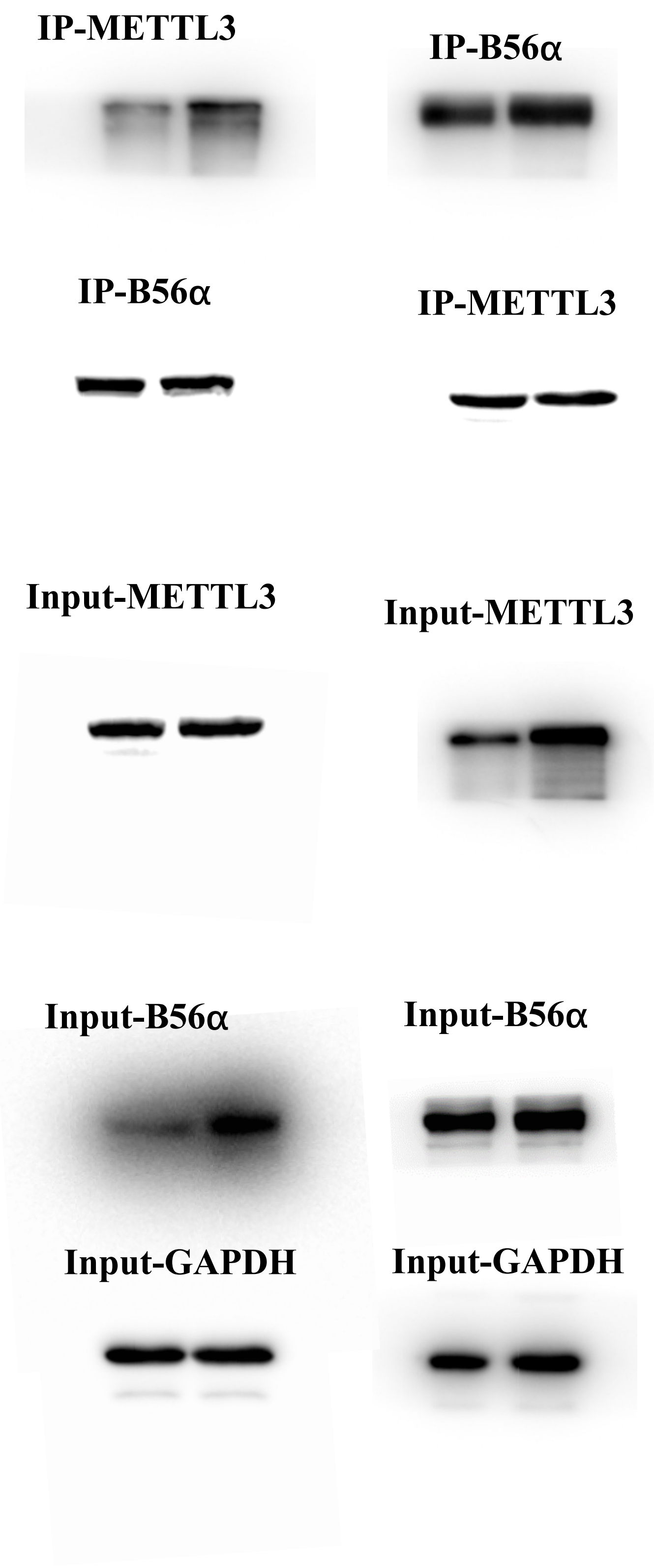
IP-METTL3
IP-B56α
IP-B56α
IP-METTL3
Input-METTL3
Input-METTL3
Input-B56α
Input-B56α
Input-GAPDH
Input-GAPDH

Fig.5I

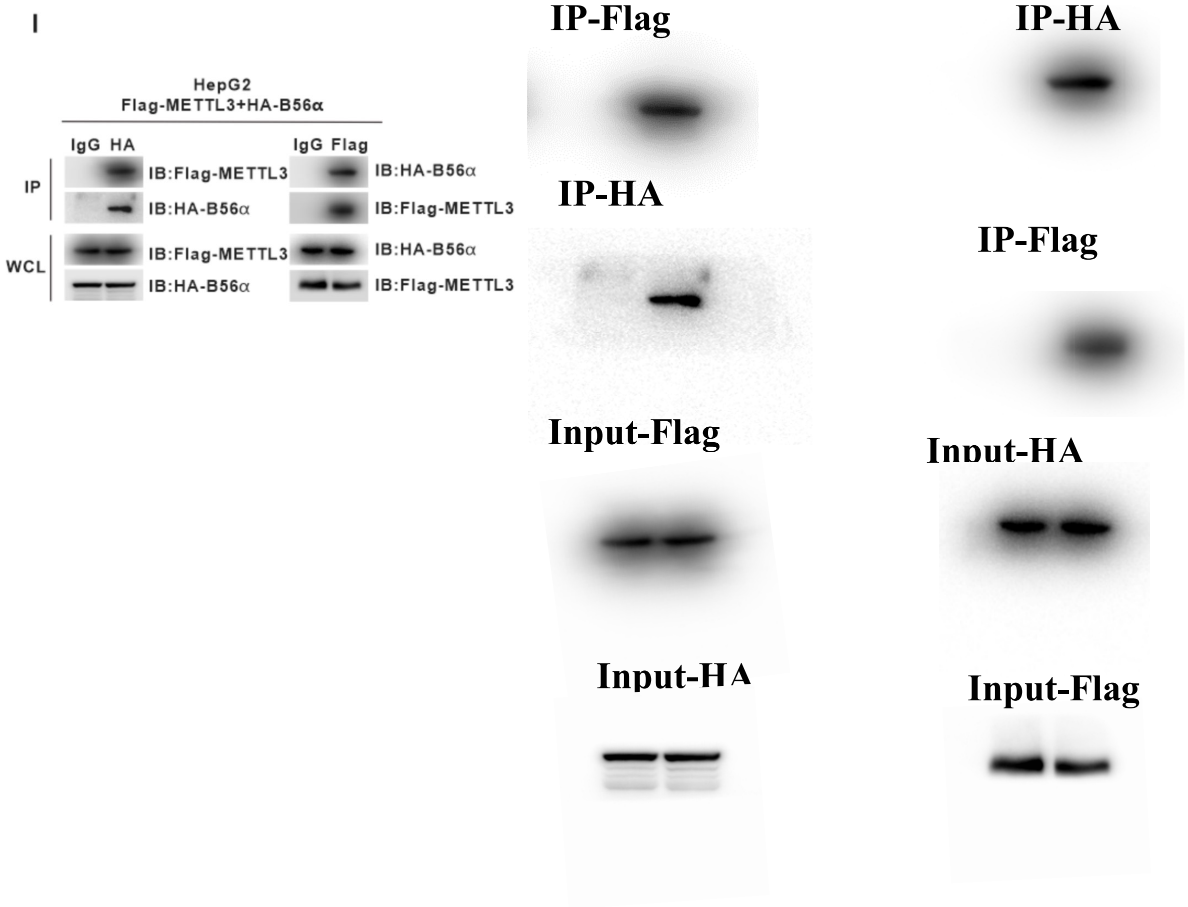
I
HepG2
Flag-METTL3+HA-B56α
IgG HA
IgG Flag
IP
WCL
IB:Flag-METTL3
IB:HA-B56α
IB:Flag-METTL3
IB:HA-B56α
IB:HA-B56α
IB:Flag-METTL3
IB:HA-B56α
IB:Flag-METTL3
IP-Flag
IP-HA
IP-HA
IP-Flag
Input-Flag
Input-HA
Input-HA
Input-Flag

Fig.5L

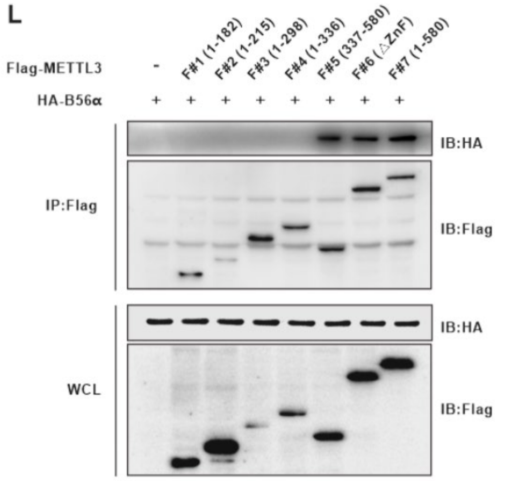
L
Flag-METTL3
-
F#1 (1-182)
F#2 (1-215)
F#3 (1-298)
F#4 (1-336)
F#5 (337-580)
F#6 (△ZnF)
F#7 (1-580)
HA-B56α
IP:Flag
WCL
IB:HA
IB:Flag
IB:HA
IB:Flag

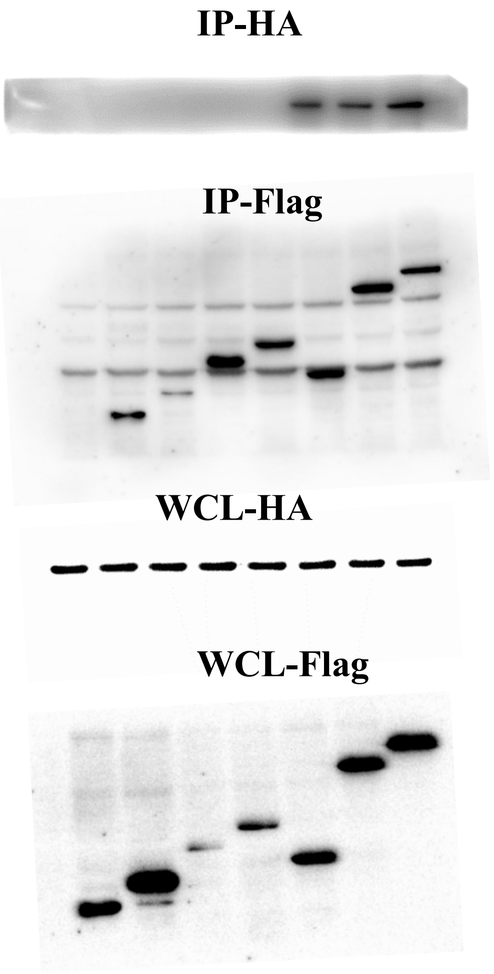
IP-HA
IP-Flag
WCL-HA
WCL-Flag

Fig.5T

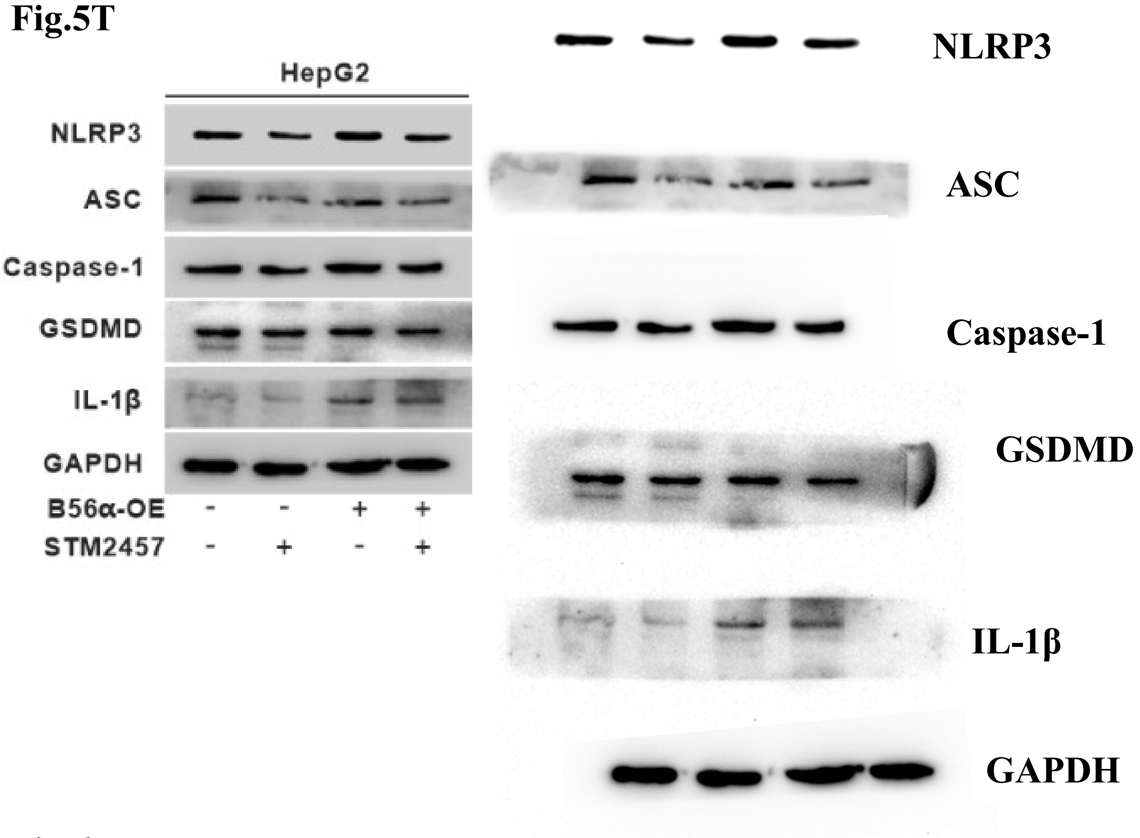
HepG2
NLRP3
ASC
Caspase-1
GSDMD
IL-1β
GAPDH
B56α-OE - - + +
STM2457 - + - +
NLRP3
ASC
Caspase-1
GSDMD
IL-1β
GAPDH

Fig.6D

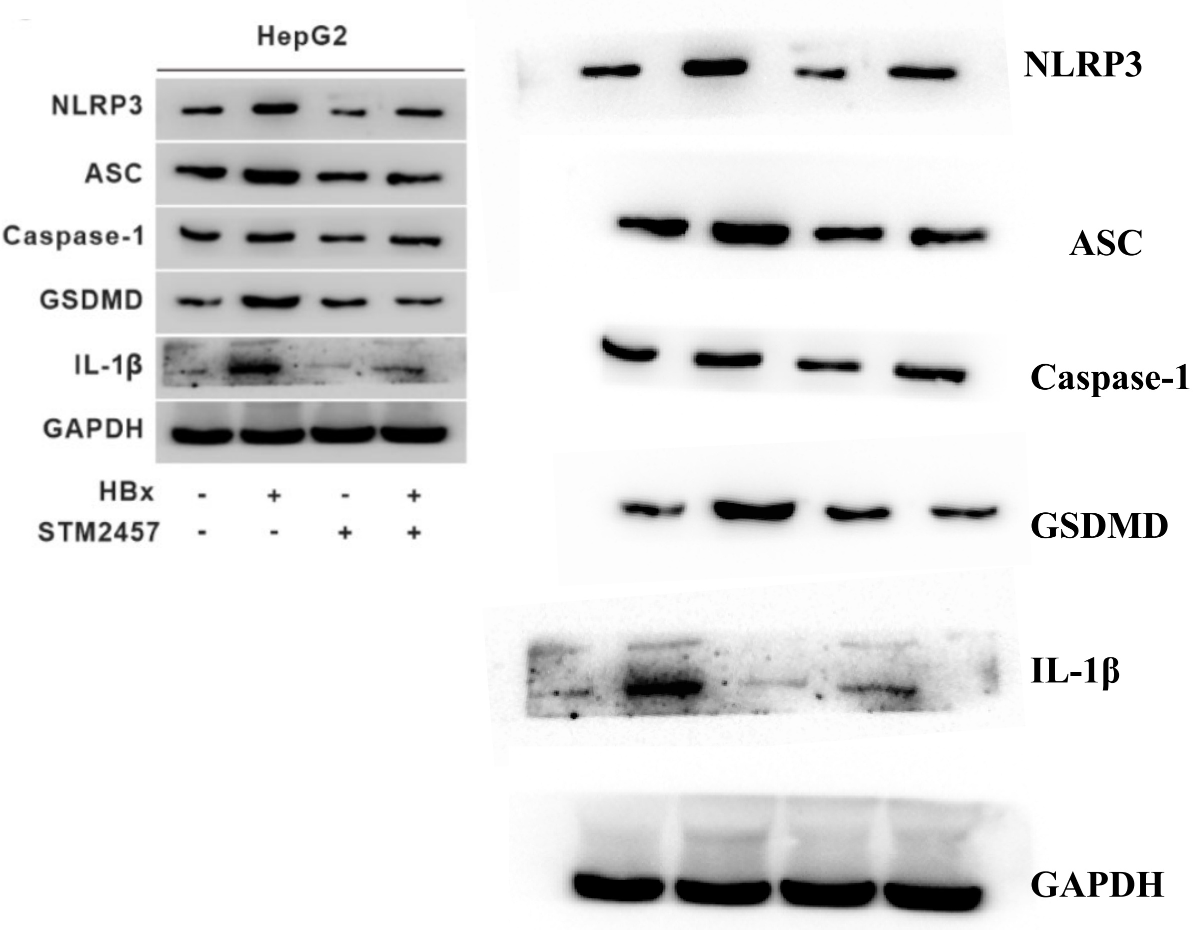
HepG2
NLRP3
ASC
Caspase-1
GSDMD
IL-1β
GAPDH
HBx - + - +
STM2457 - - + +
NLRP3
ASC
Caspase-1
GSDMD
IL-1β
GAPDH

Fig.7E

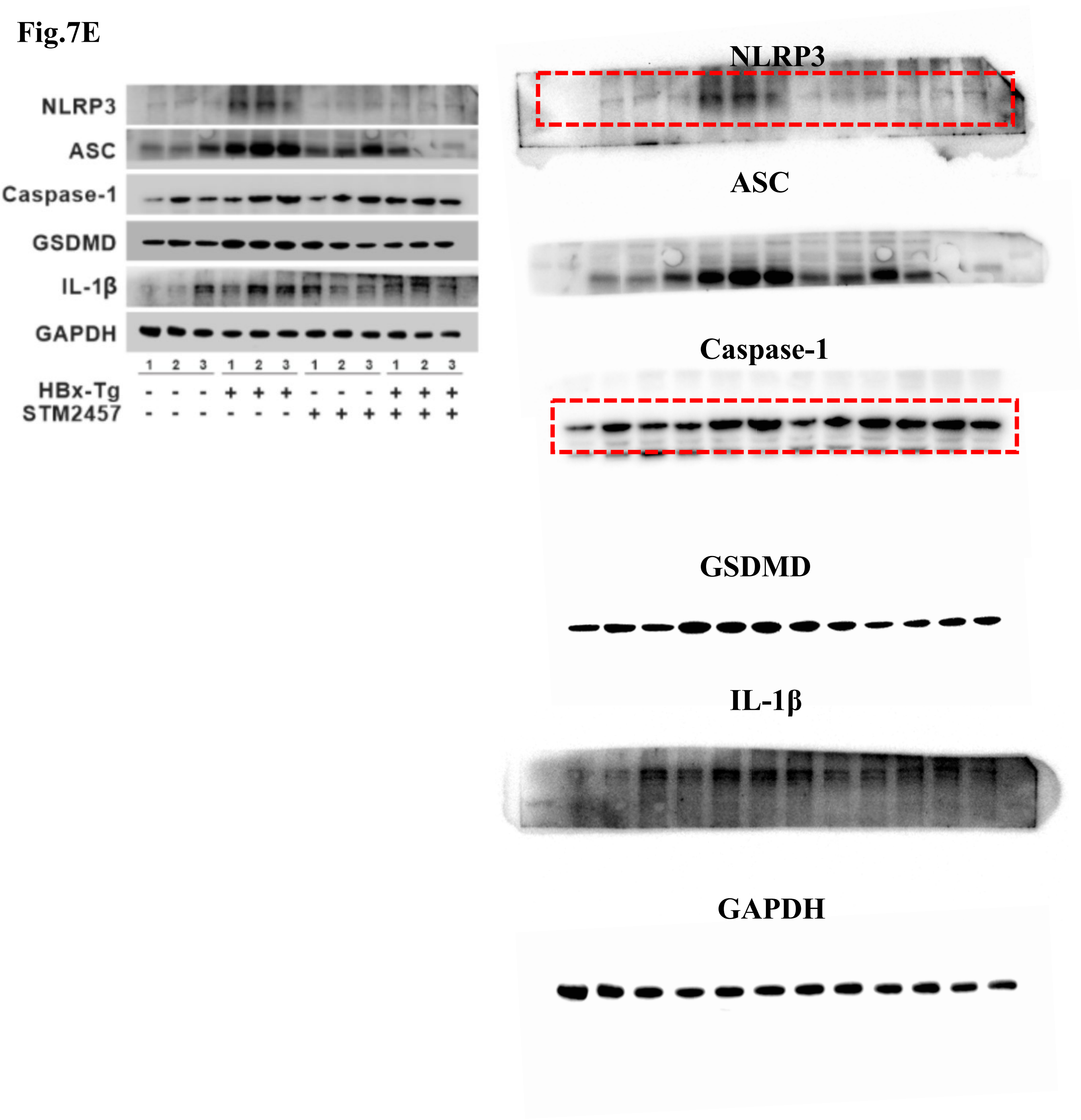

NLRP3
ASC
Caspase-1
GSDMD
IL-1β
GAPDH
1 2 3 1 2 3 1 2 3 1 2 3
HBx-Tg - - - + + + - - - + + +
STM2457 - - - - - - + + + + + +
NLRP3
ASC
Caspase-1
GSDMD
IL-1β
GAPDH

Fig.7F

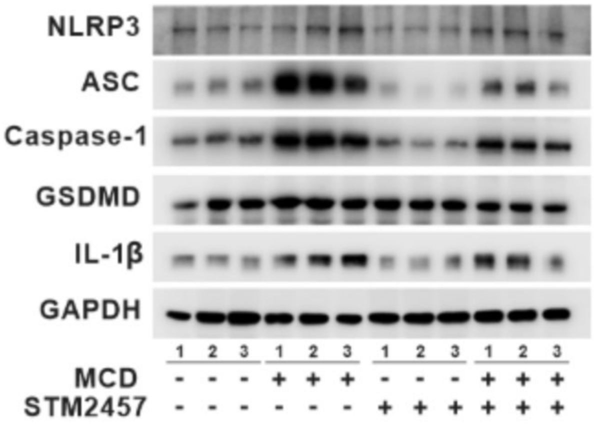
NLRP3
ASC
Caspase-1
GSDMD
IL-1β
GAPDH
1 2 3 1 2 3 1 2 3 1 2 3
MCD - - - + + + - - - + + +
STM2457 - - - - - - + + + + + +

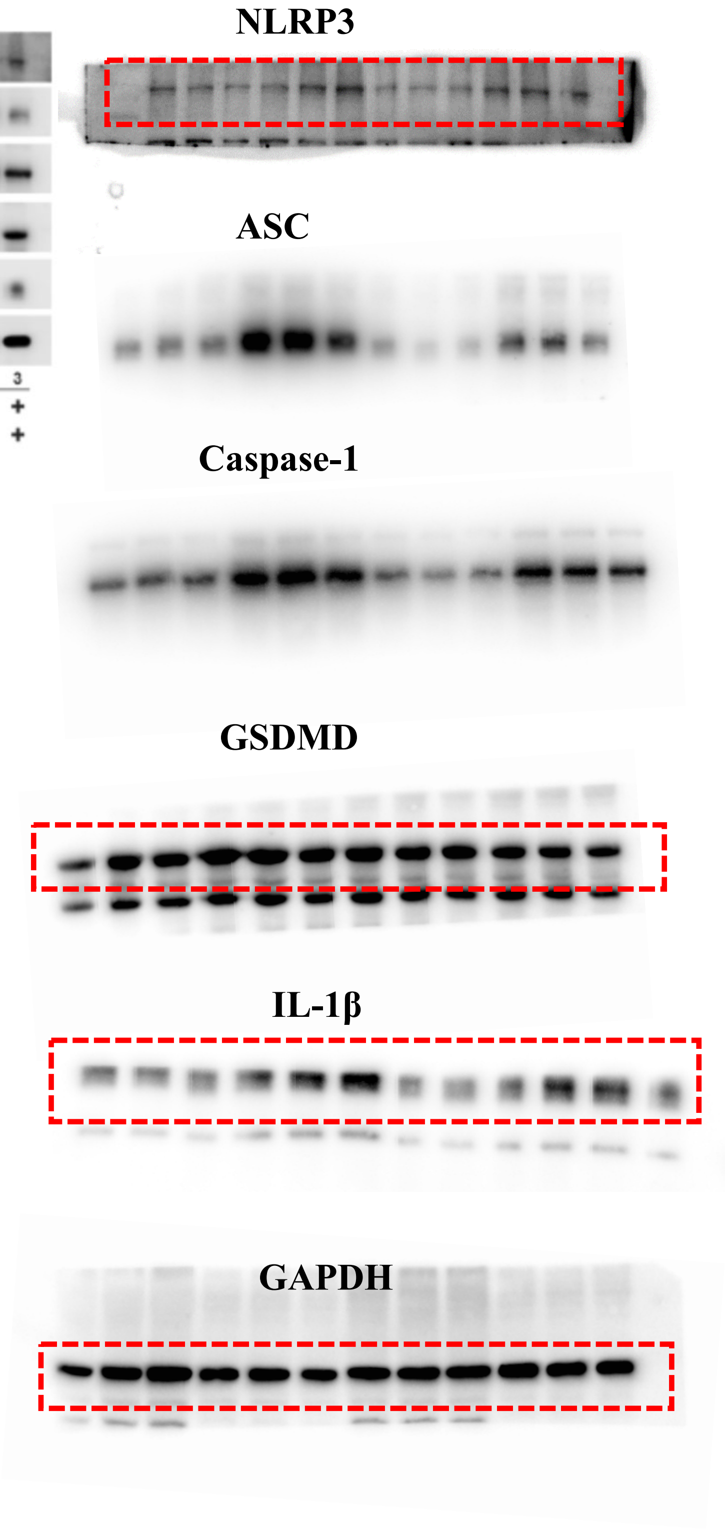
NLRP3
ASC
Caspase-1
GSDMD
IL-1β
GAPDH

Fig.S2D

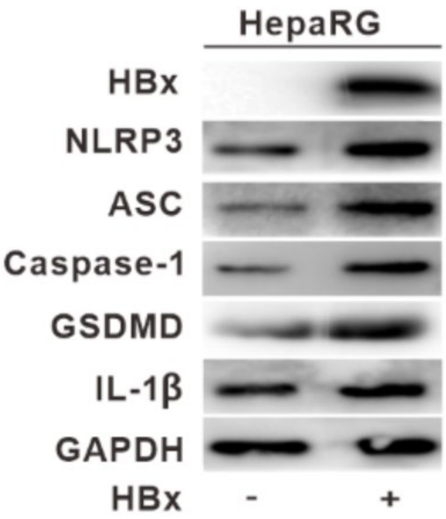

HepaRG
HBx
NLRP3
ASC
Caspase-1
GSDMD
IL-1β
GAPDH
HBx - +

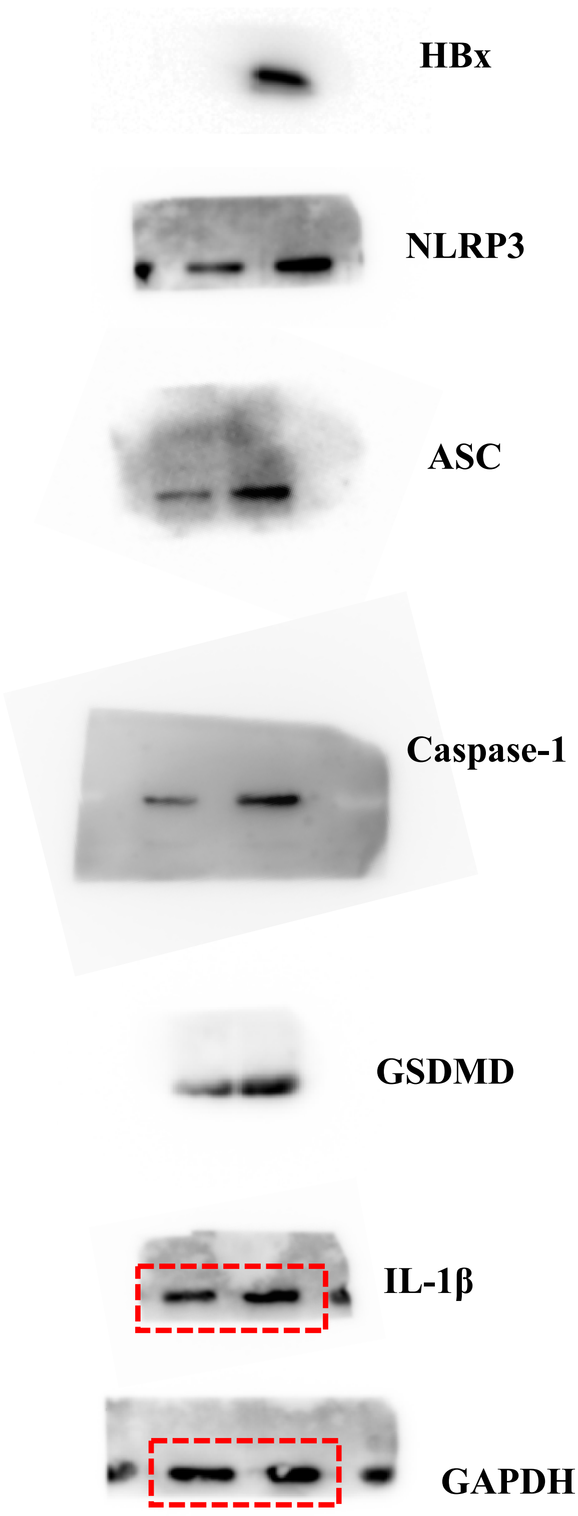

HBx
NLRP3
ASC
Caspase-1
GSDMD
IL-1β
GAPDH

Fig.S2L

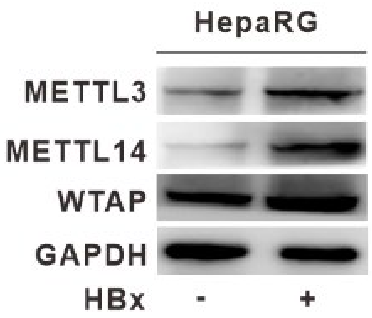

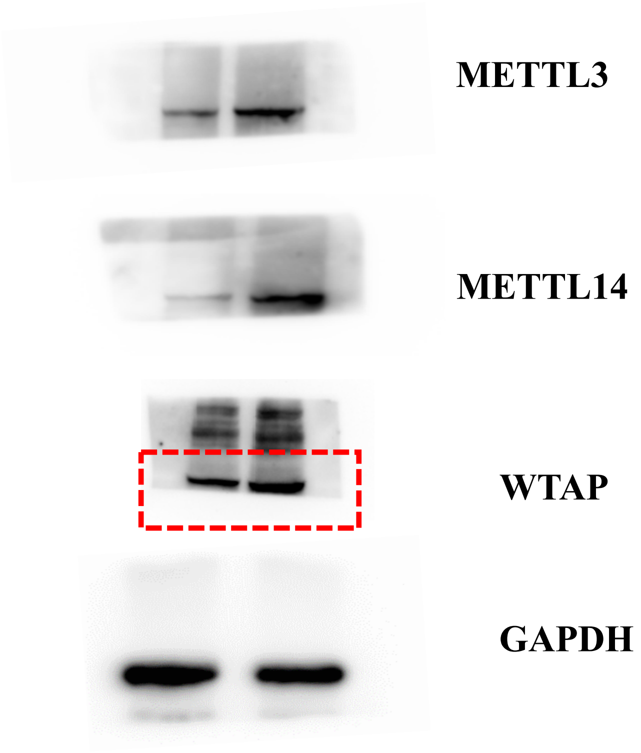

Fig.S3A

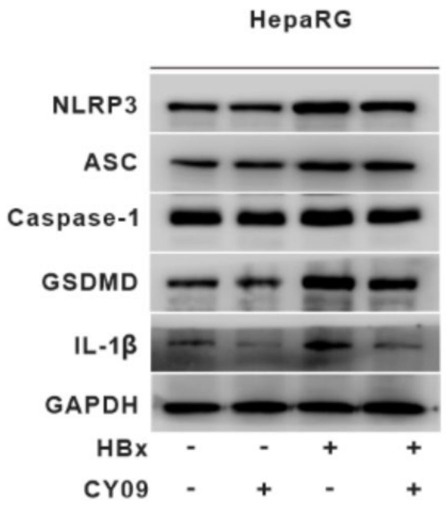

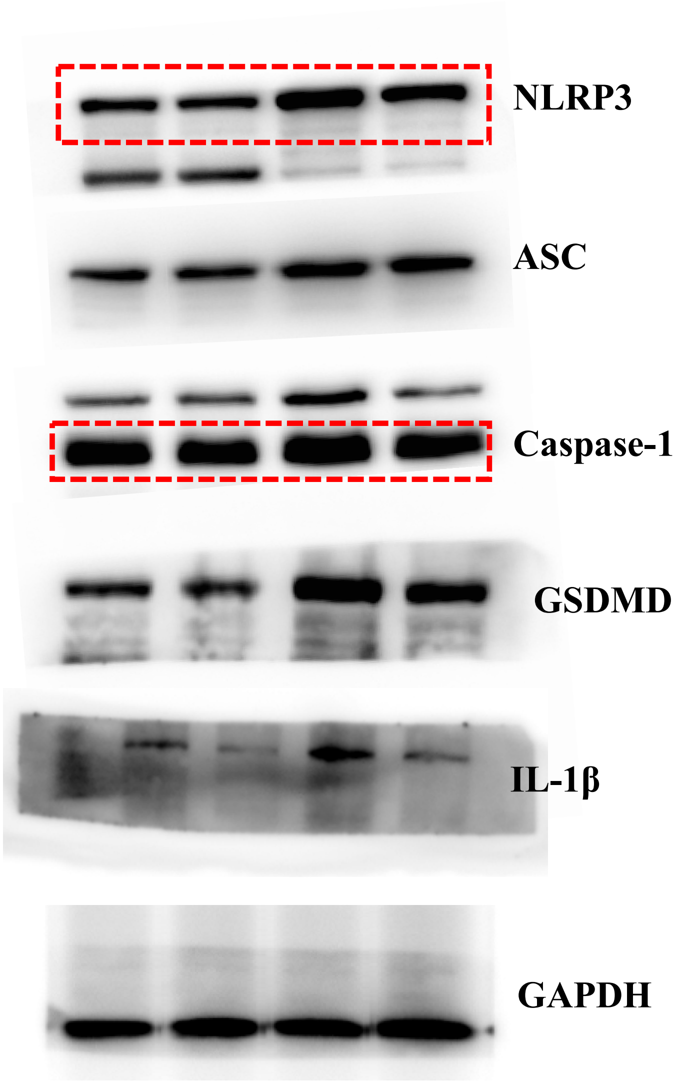

Fig.S3F

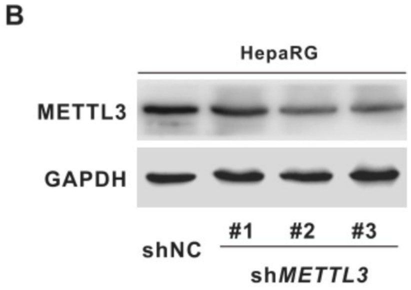

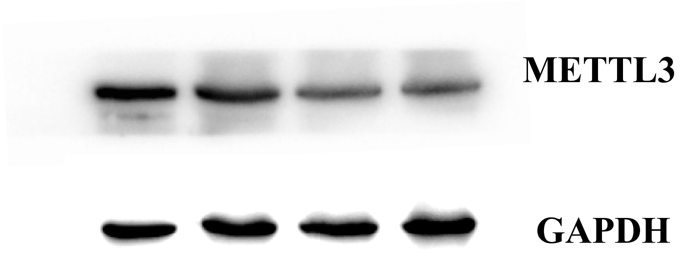

Fig.S3H

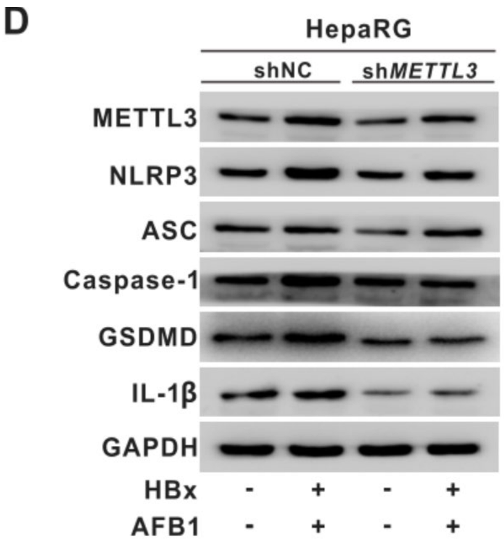

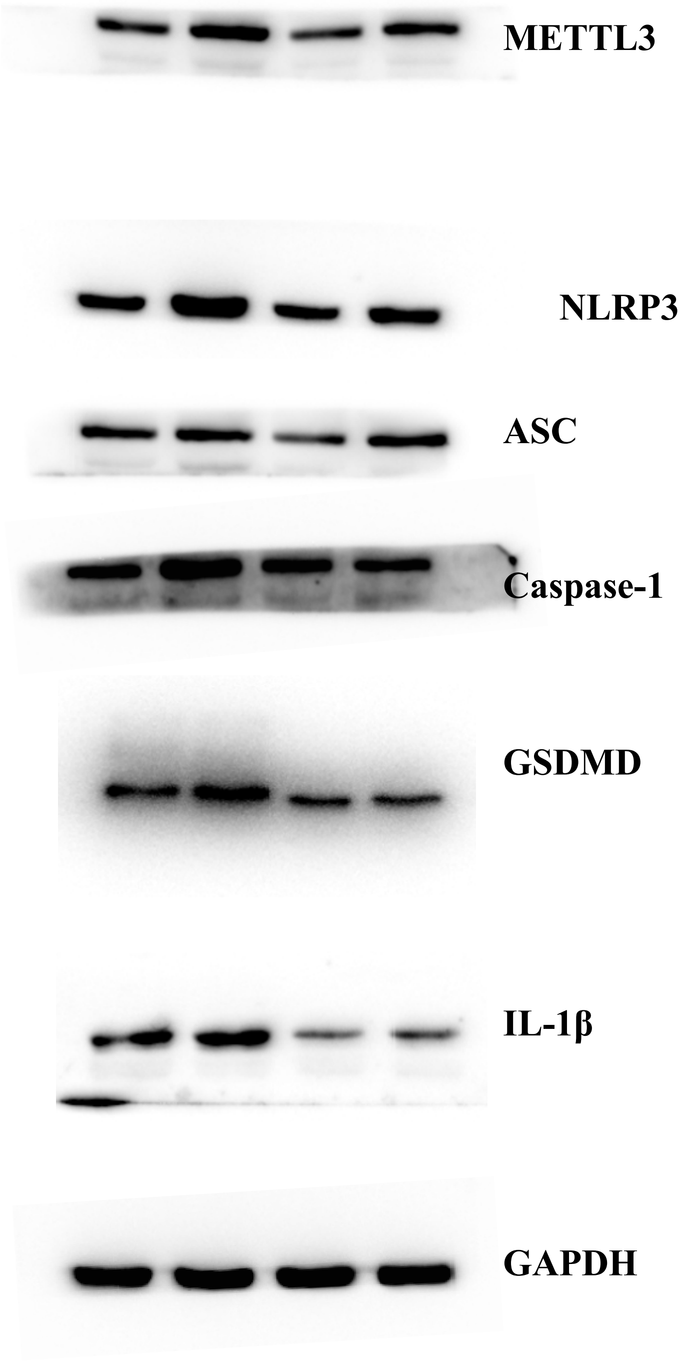

Fig.S3M

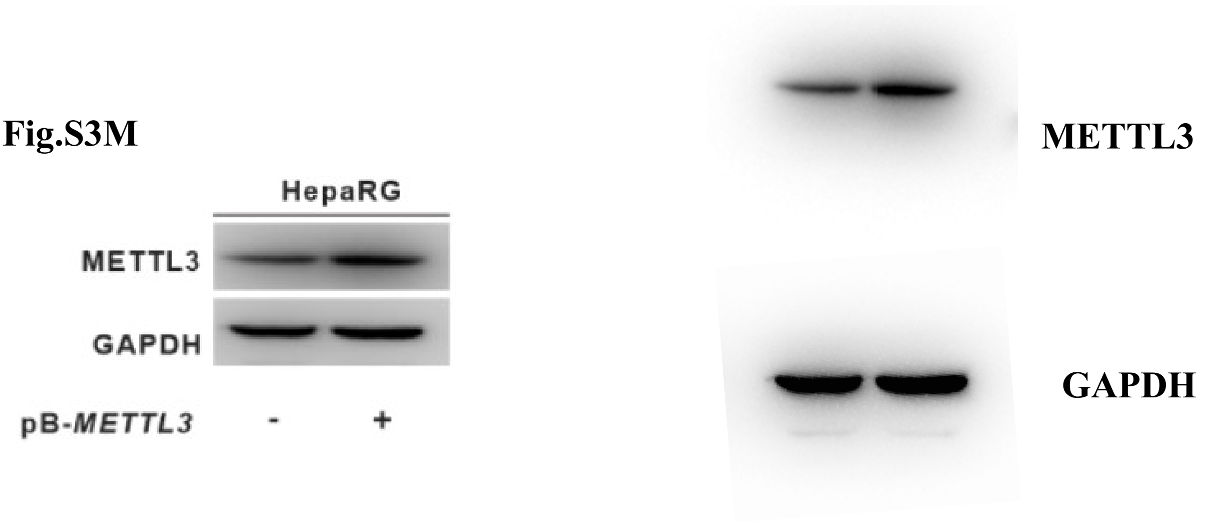

Fig.S3O

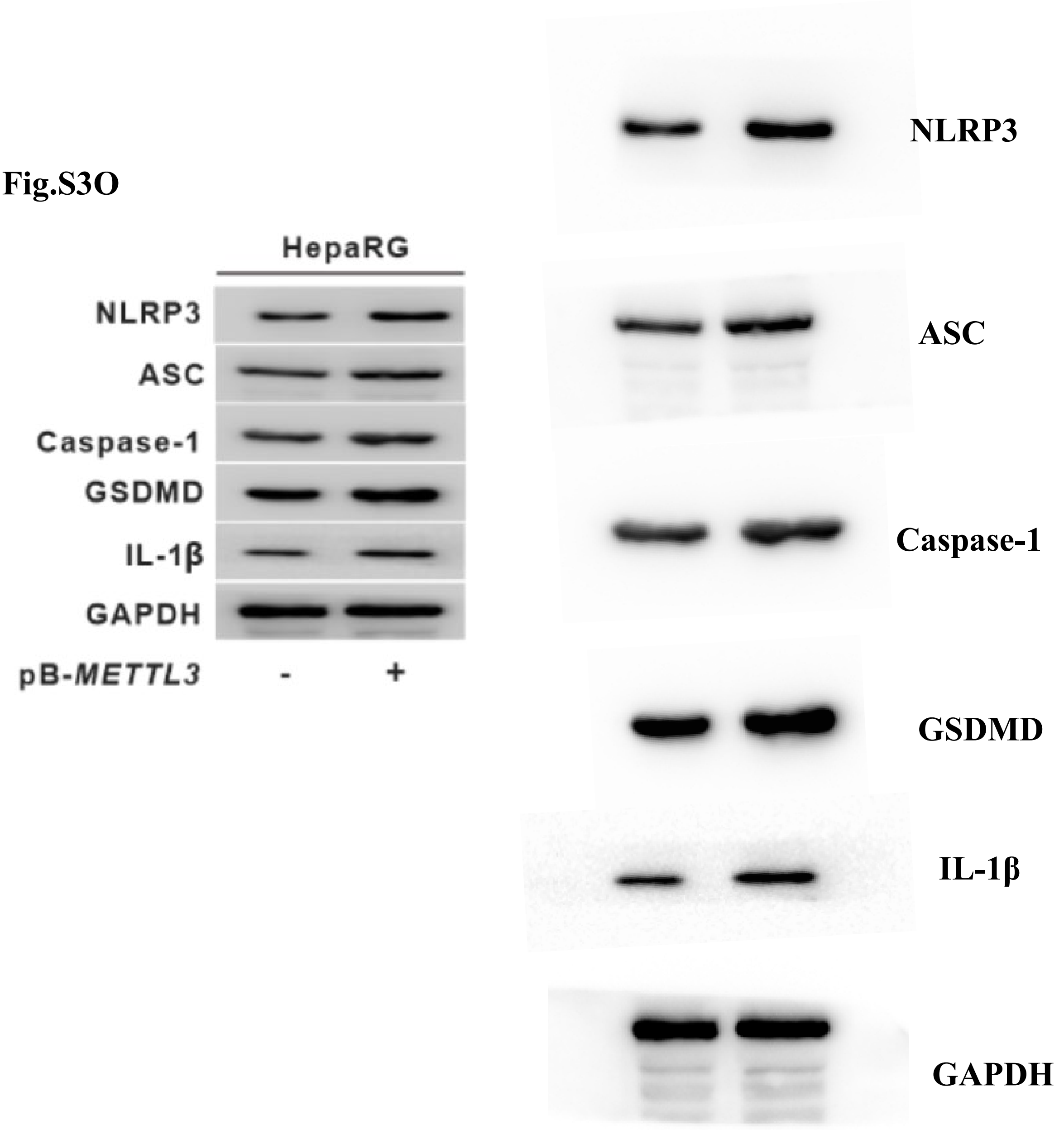

Fig.S3P

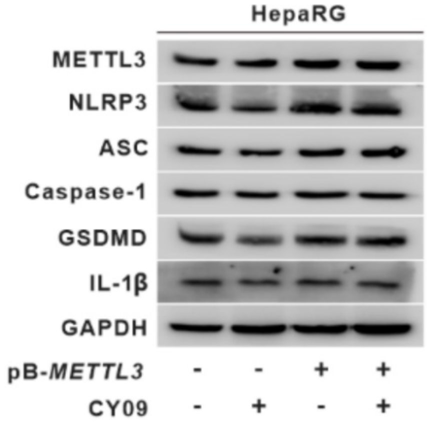
HepaRG
METTL3
NLRP3
ASC
Caspase-1
GSDMD
IL-1β
GAPDH
pB-*METTL3* - - + +
CY09 - + - +

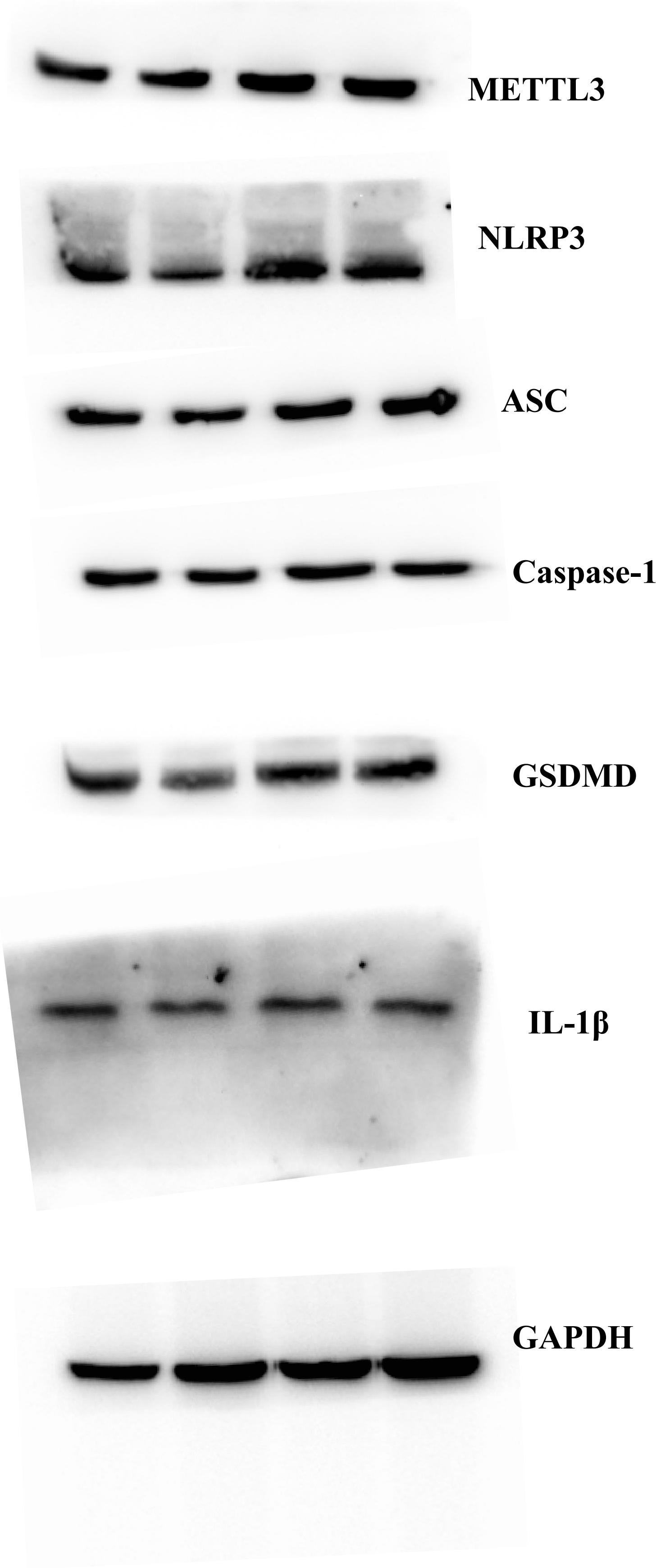
METTL3
NLRP3
ASC
Caspase-1
GSDMD
IL-1β
GAPDH

Fig.S4C

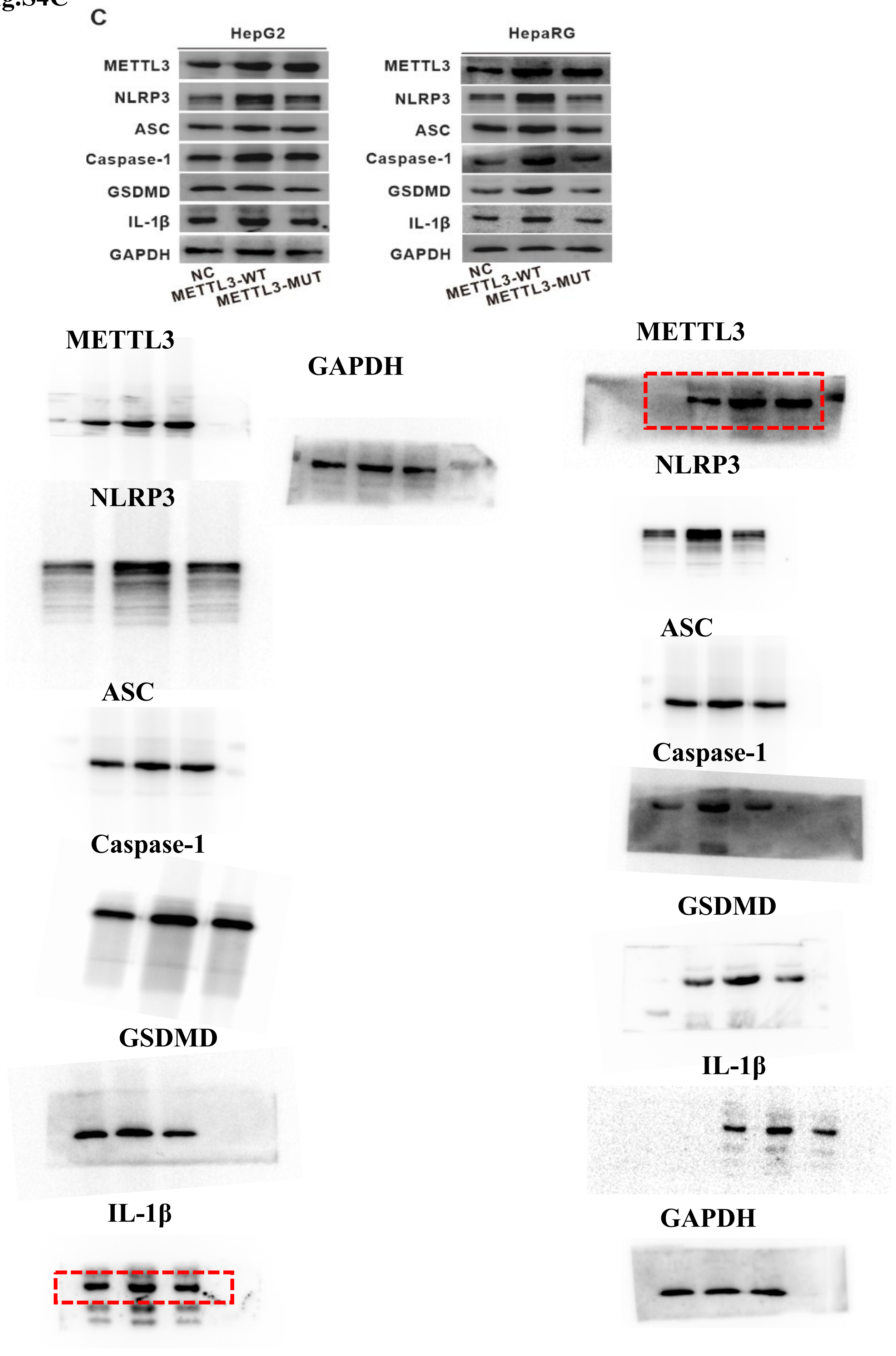
C
HepG2
HepaRG
METTL3
NLRP3
ASC
Caspase-1
GSDMD
IL-1β
GAPDH
NC
METTL3-WT
METTL3-MUT
METTL3
GAPDH
METTL3
NLRP3
NLRP3
ASC
ASC
Caspase-1
Caspase-1
GSDMD
GSDMD
IL-1β
IL-1β
GAPDH

Fig.S4E

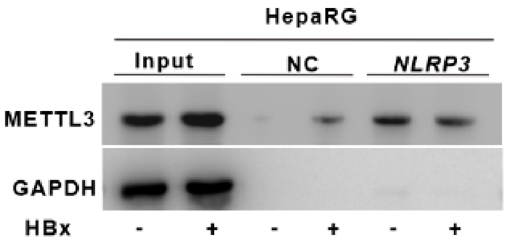

METTL3

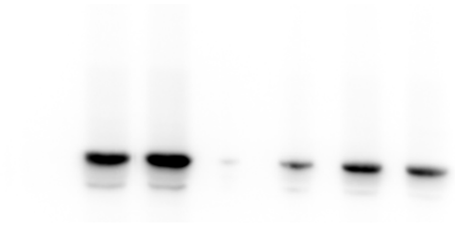

GAPDH

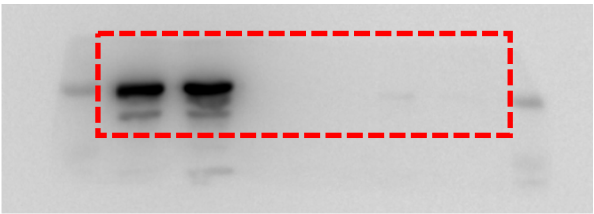

Fig.S5J

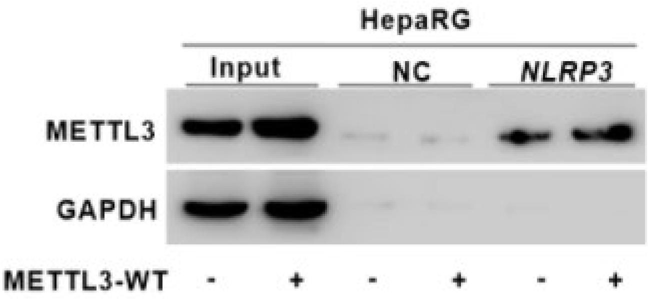

METTL3

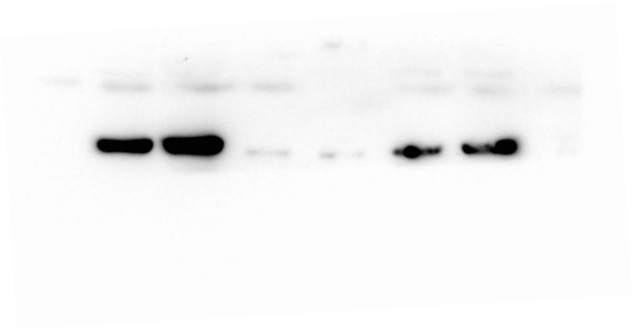

GAPDH

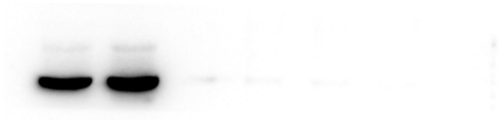

Fig.S6B

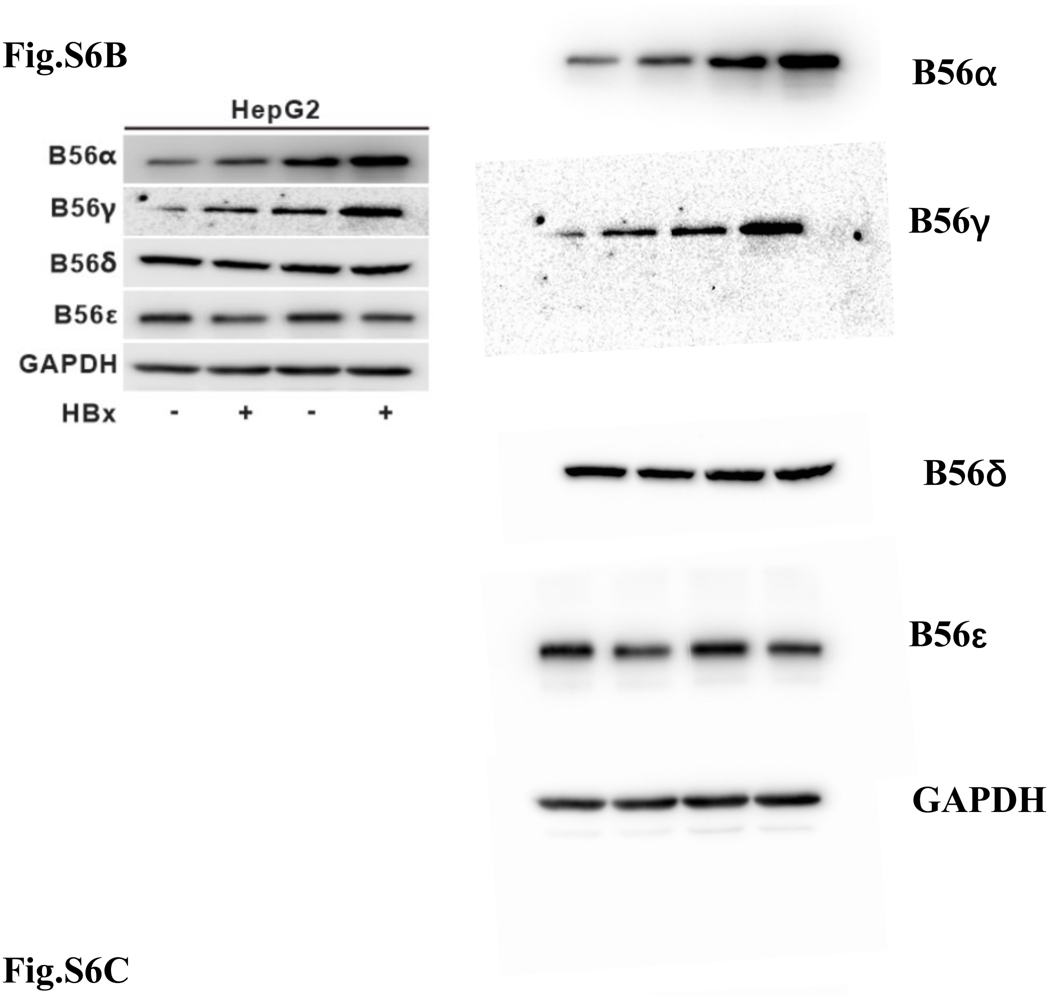

Fig.S6C

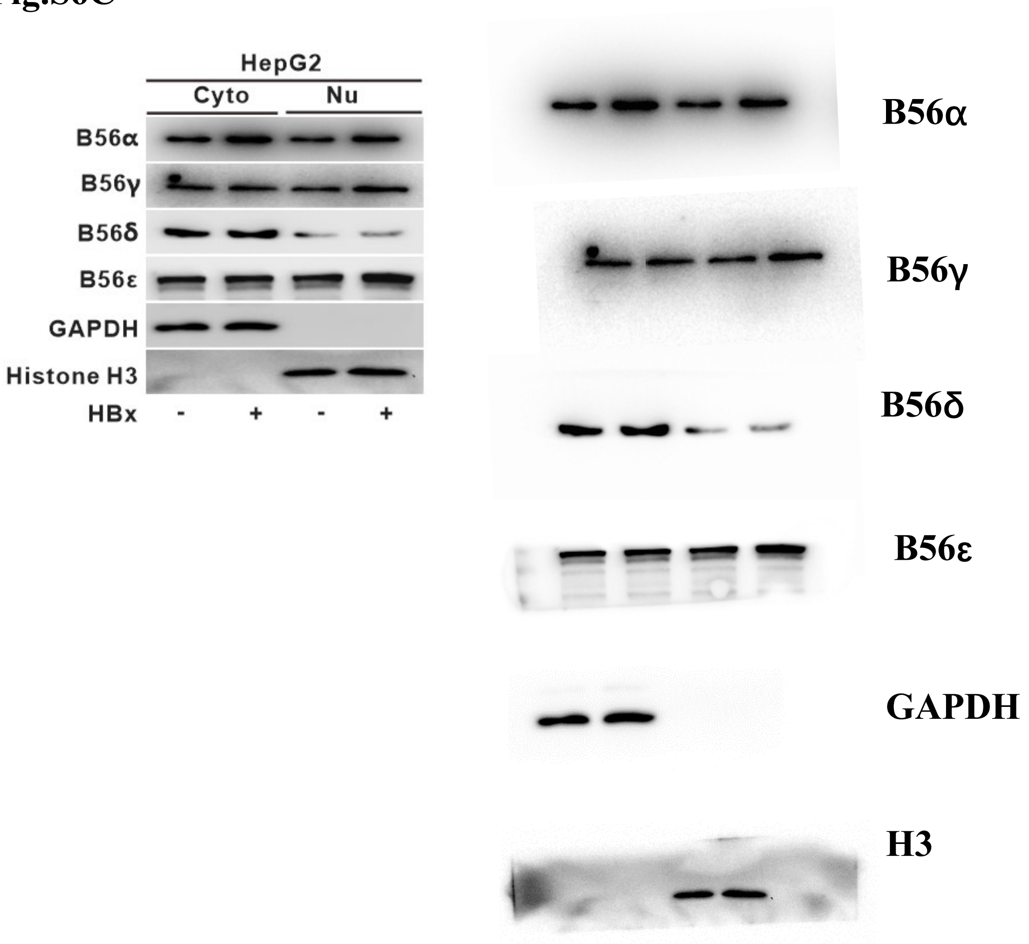

Fig.S6E

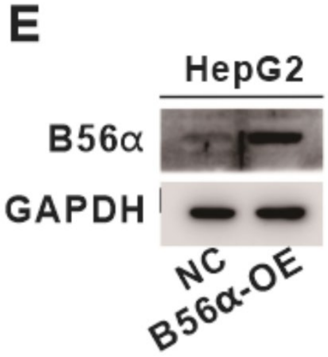

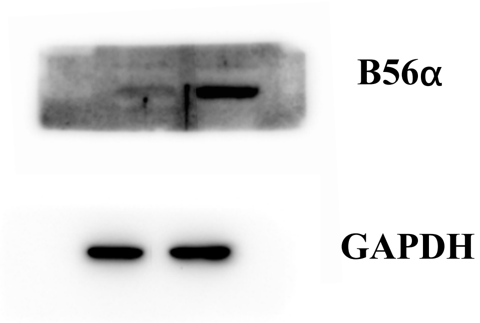

Fig.S6F

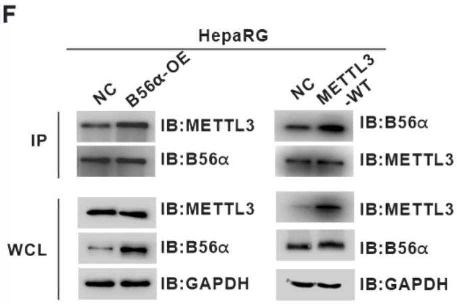

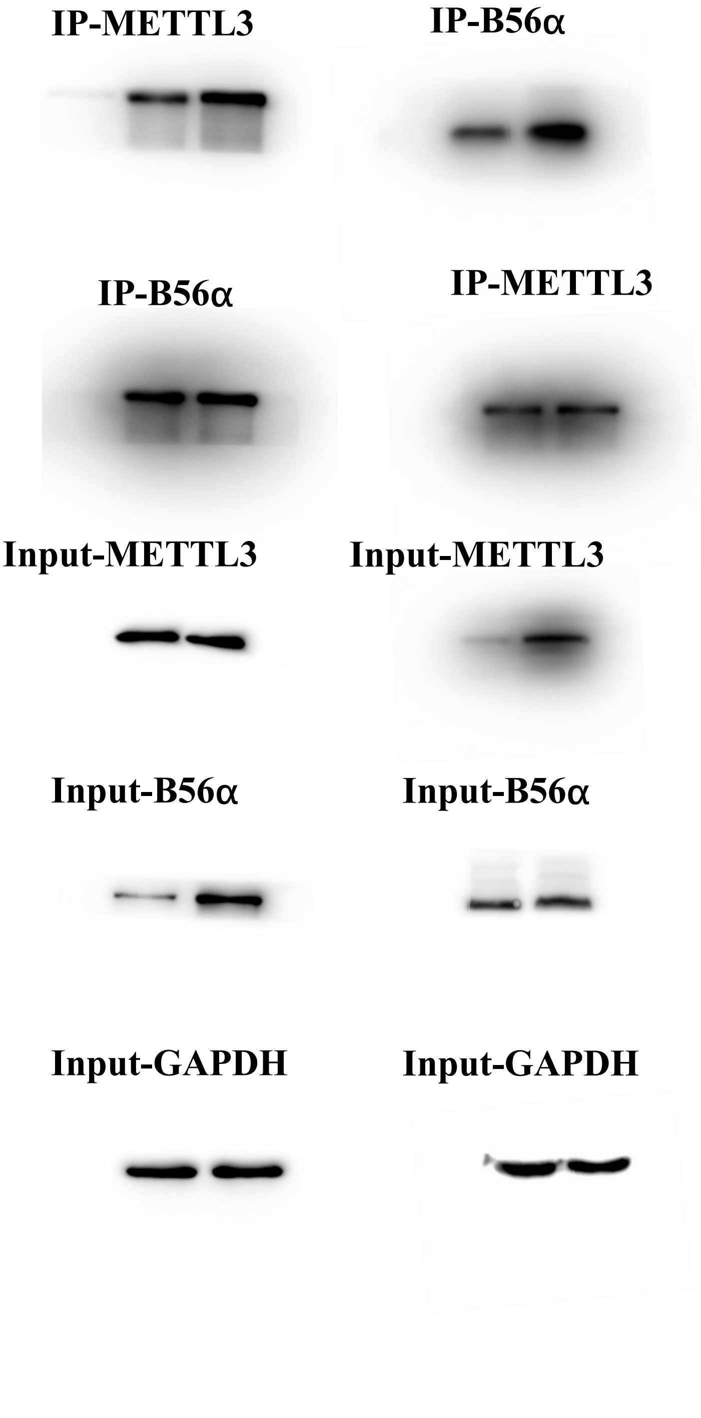

Fig.S6G

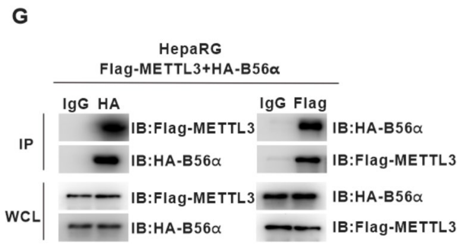

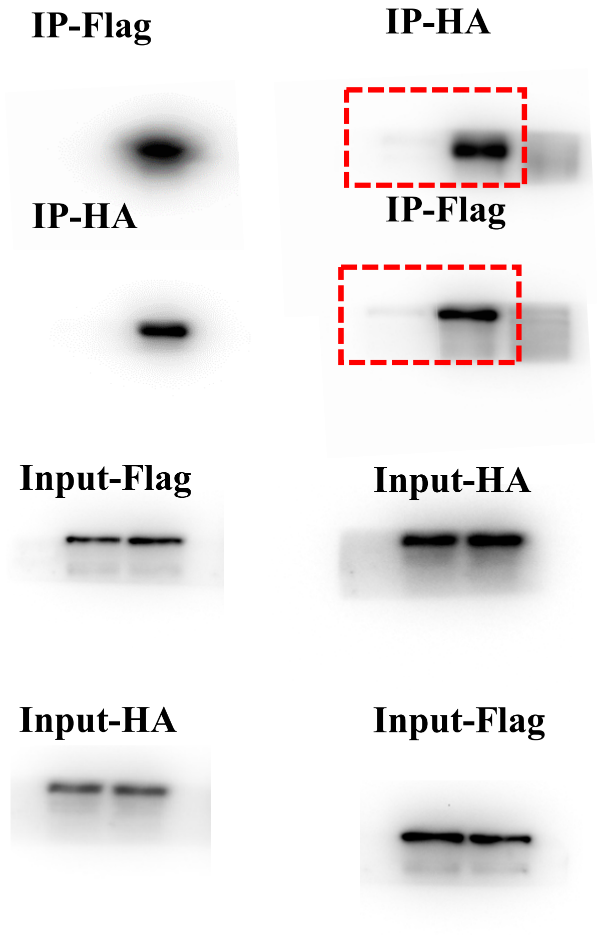

Fig.S6I

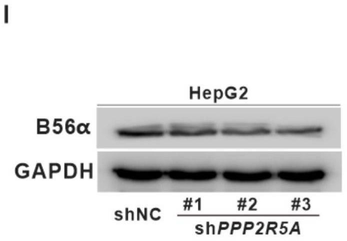

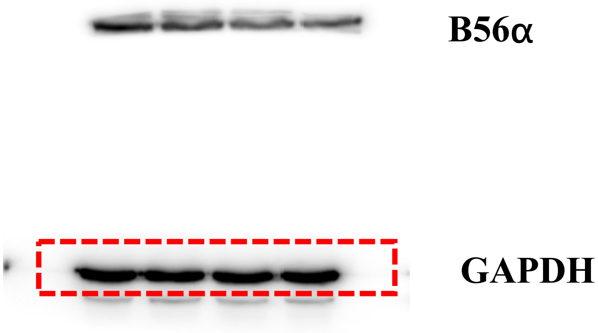

Fig.S6M

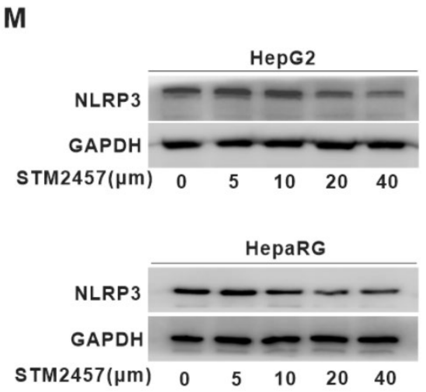

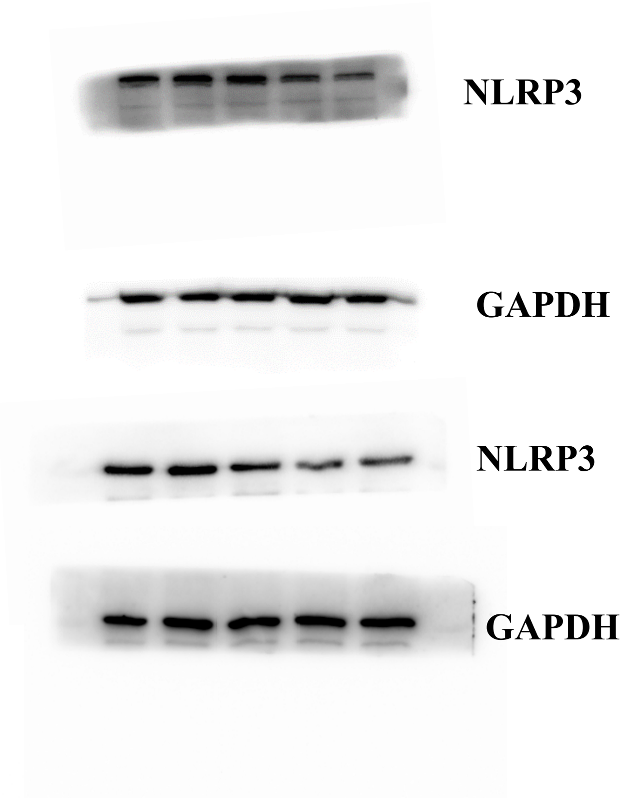

Fig.S7D

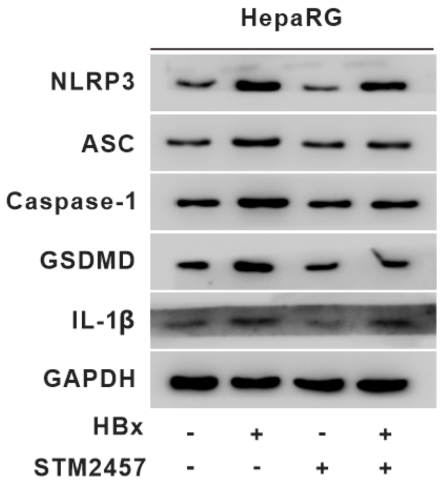

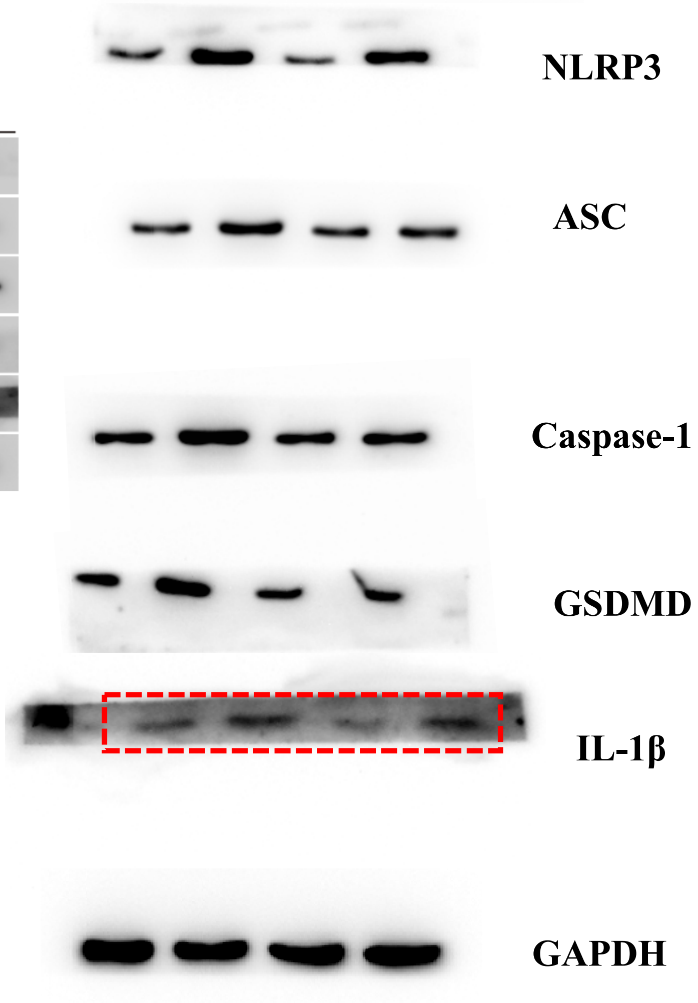

Fig.S8B

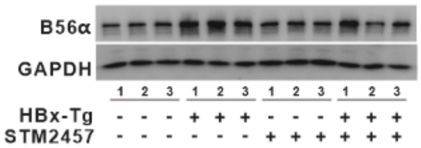

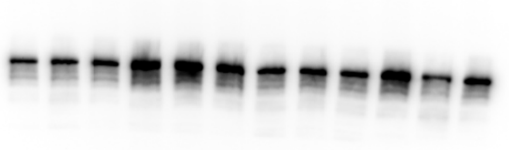

B56α

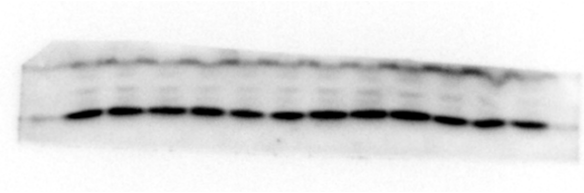

GAPDH

Fig.SC

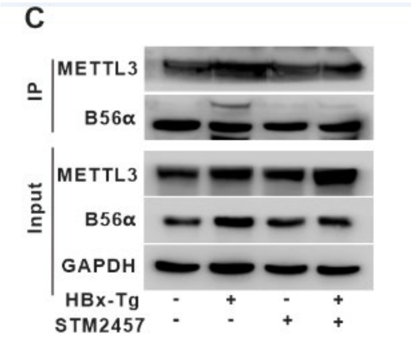

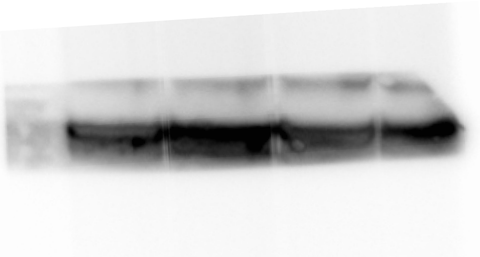

IP-METTL3

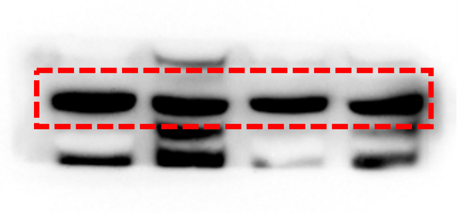

IP-B56α

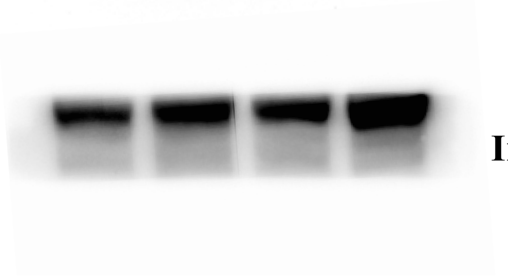

Input-METTL3

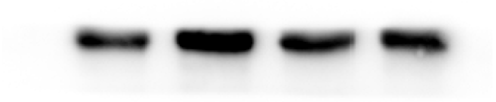

Input-B56α

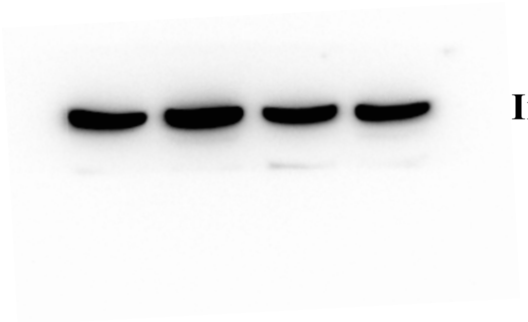

Input-GAPDH
